# Carborane Hydrophobic Tags Drive Selective Degradation of Endogenous KRAS^G12C^ via HSP70–Ubiquitin–Proteasome Pathway

**DOI:** 10.1021/acsbiomedchemau.6c00004

**Published:** 2026-03-10

**Authors:** Yujie Shao, Kazuki Miura, Hiroyuki Nakamura

**Affiliations:** † School of Life Science and Technology, 693022 Institute of Science Tokyo, 4259 Nagatsuta-cho Midori-ku, Yokohama 226-8501, Japan; ‡ Laboratory for Chemistry and Life Science, Institute of Integrated Research, 693022 Institute of Science Tokyo, 4259 Nagatsuta-cho Midori-ku, Yokohama 226-8501, Japan

**Keywords:** carborane, Kirsten rat sarcoma viral oncogene homologue (KRAS), hydrophobic tag (HyT), targeted protein degradation (TPD), heat shock protein 70-mediated degradation

## Abstract

Persistent activation of the Kirsten rat sarcoma viral oncogene homologue (KRAS)^G12C^ mutation sustains oncogenic signaling, and covalent inhibitors often yield incomplete pathway suppression or resistance. Herein, we developed hydrophobic-tag (HyT) degraders by introducing a carborane cluster into MRTX849, an KRAS^G12C^ covalent ligand, to promote selective elimination of endogenous KRAS^G12C^. The carborane-HyT conjugates (**HY8**) reduced KRAS^G12C^ abundance and ERK phosphorylation in mutant cancer cells while showing lower intrinsic cytotoxicity and activity equal or superior to the adamantane-HyT analogue (**HY5**). Mechanistic studies indicate that **HY8** acts in an E3 ligase-independent, chaperone-assisted pathway. KRAS degradation was rescued by the proteasome inhibitor MG132, but not by the lysosomal inhibitor bafilomycin A1, consistent with the increased level of KRAS ubiquitination and heat shock protein 70 engagement. Competition with MRTX849 blocked KRAS degradation, supporting on-target covalent engagement at Cys12. These findings establish carborane as a compact, functional HyT that drives proteasome-dependent degradation of endogenous KRAS^G12C^ and suppresses downstream signaling, broadening degrader design to include an E3-independent modality for the degradation of oncogenic proteins.

## Graphical abstract

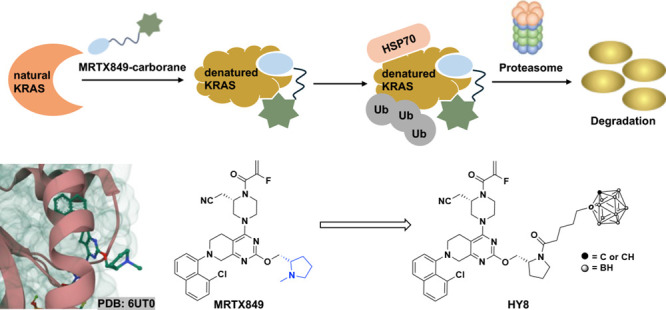

## Introduction

Targeted protein degradation (TPD) has emerged as a promising therapeutic paradigm for eliminating disease-related proteins that are otherwise considered to be undruggable. Among the various TPD technologies, proteolysis targeting chimeras (PROTACs) have attracted considerable attention. PROTACs are bifunctional molecules that recruit a protein of interest (POI) to an E3 ubiquitin ligase, promoting ubiquitination and proteasomal degradation. Since the first peptide PROTAC was introduced in 2001,^1^ numerous PROTACs have been developed for diverse therapeutic targets,^2–6^ and several candidates, such as ARV-110 (androgen receptor (AR)),^7,8^ ARV-471 (estrogen receptor (ER)),^9^ NX-2127 (Bruton’s tyrosine kinase (BTK)),^10,11^ DT2216 (B cell lymphoma extra large (BCL-X_L_)),^12^ have entered clinical trials. Despite their success, the clinical transformation of PROTACs remains challenging due to their large molecular weight, poor cell permeability, and unpredictable pharmacokinetics.

To overcome these limitations, the hydrophobic tagging (HyT) strategy was developed as an alternative E3-independent degradation approach. In this concept, a small hydrophobic tag is conjugated to a selective ligand of the POI. Upon binding, the exposed hydrophobic surface mimics a misfolded or denatured protein, triggering recognition by molecular chaperones and subsequent degradation through the proteasomes or lysosomes.^13,14^ The hydrophobic tag structures generally have lower molecular weight than E3 ligands and avoid potential teratogenicity associated with thalidomide-derived cereblon (CRBN) ligands. Although various hydrophobic tags, such as adamantane,^15^ boc_3_Arg,^16^ fluorene,^17^ norbornene,^18^ have been explored, the discovery of novel hydrophobic tags with improved efficiency and physicochemical diversity remains a key challenge.

Carborane, a three-dimensional (3D) *closo*-boron cluster consisting of ten boron and two carbon atoms,^19^ offers a unique chemical topology that combines high hydrophobicity, chemical robustness, and compactness. Owing to its exceptionally high hydrophobicity and compact 3D geometry, carborane represents a distinctive class of hydrophobic tags beyond conventional hydrocarbon scaffolds such as adamantane. We previously demonstrated that conjugation of *meta*- and *ortho*-carboranes to a HaloTag ligand induced efficient intracellular protein degradation through a proteasome-dependent mechanism.^20^ However, it remained unclear whether this system could be applied to endogenous proteins and which compound's structural features are critical for its activity. In this study, based on our previous studies, we selected *meta*- and *ortho*-carborane derivatives with various linker lengths as hydrophobic tags to develop a synthetically accessible carborane-based HyT system that retains endogenous protein degradation-inducing activity.

Building on this concept, we herein applied the carborane-based HyT strategy to the degradation of the Kirsten rat sarcoma viral oncogene homologue (KRAS), which is one of the most frequently mutated oncogenic derivers in human cancers. Activating mutations in KRAS cause persistent accumulation of the GTP-bound active form, thereby sustaining downstream proliferative signaling.^21–23^ Among them, KRAS^G12C^ mutation generates a unique cysteine residue that enables covalent ligand binding, overcoming the long-standing challenges of directly targeting KRAS. Several irreversible covalent KRAS^G12C^ inhibitors, including AMG-510 (sotorasib) and MRTX849 (adagrasib), have been clinically approved or advanced to late-stage trials.^24–26^ Moreover, the LC-2 degrader developed by Crews group^27^ validated that KRAS^G12C^ can also be eliminated through a PROTAC-based mechanism (Figure 1A). Encouraged by these advances, we constructed a series of MRTX849-HyT conjugates in which MRTX849 acts as the KRAS^G12C^-binding warhead and carborane functions as a compact hydrophobic tag. We systemically evaluated their degradation efficacy toward endogenous KRAS^G12C^ and investigated their underlying mechanism, focusing on the molecular chaperones and proteolytic pathways involved (Figure 1B,C).

**Figure 1:**
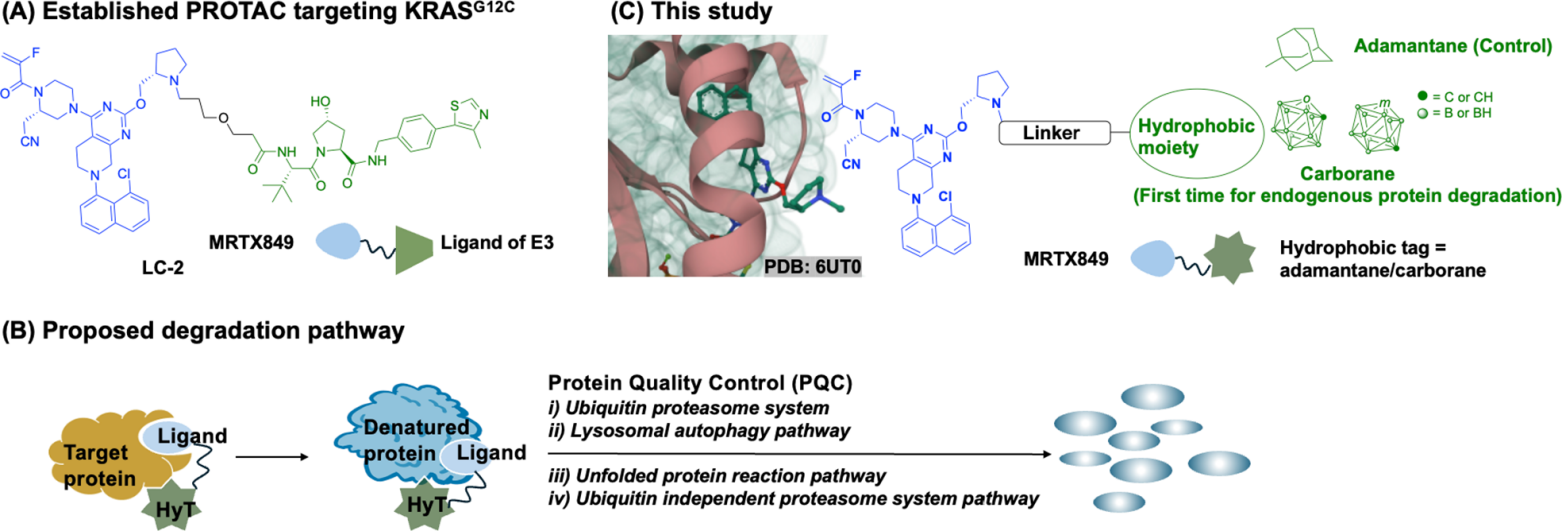
Overview of this study. (A) Chemical structure of the reported PROTAC (LC-2) that targets KRAS^G12C^ via an E3 ligase-dependent mechanism. (B) Schematic of hydrophobic tag (HyT)-induced degradation, highlighting E3-independent, chaperone-assisted recognition of misfolded proteins and routing to the ubiquitin-proteasome system. (C) Molecular design of the MRTX849-HyT conjugates developed in this study, in which the carborane cluster functions as a compact HyT for selective degradation of endogenous KRAS^G12C^.

## Results and Discusstion

### Synthesis of KRAS^G12C^ Degraders Based on Hydrophobic Tagging System

To design potent degraders targeting KRAS^G12C^, we focused on MRTX849, a clinically validated covalent inhibitor that reacts with Cys12 in their “switch II” pocket of KRAS^G12C^ through its acrylamide moiety (PDB: 6UT0, Figures 1C and S1).^24,27^ Structural analysis based on the reported crystal structure revealed that the C2 position of MRTX849 forms a salt bridge with Glu62, while the *N*-methyl group on the pyrrolidine ring is solvent-exposed and positioned away from the binding interface. Therefore, this position was selected as a suitable site for hydrophobic-tag conjugation, enabling the introduction of a bulky group without compromising covalent engagement at Cys12 (Figure 1C).

The synthetic route (Scheme 1) was adapted from the literature.^24,27^ Briefly, starting from compounds **1a** (R = *tert*-butoxycarbonyl (Boc)) and **1b** (R = CH_2_CH_2_NHBoc), prepared in six steps from 1-(*tert*-butyl) 4-ethyl 3-oxopiperidine-1,4-dicarboxylate, the benzyloxycarbonyl (Cbz) group on **1a** was removed by hydrogenolysis. The resulting amines were then acylated with 2-fluoroacrylic acid to afford compound **2a** (Method A). Subsequent Boc deprotection, followed by coupling with hydrophobic acids, 2-(admantan-1-yl)­acetic acid and adamantane carboxylic acid, provided the MRTX849-HyT conjugates **HY1** and **HY2**, respectively. Alternatively, Boc deprotection of compound **1** was first carried out, and the resulting intermediates were coupled with 2-(adamantan-1-yl)­acetic acid, adamantane carboxylic acid, or carborane-based acids with the coupling reagent COMU to give the corresponding amides **3a**–**f,** ^28,29^ and subsequent Cbz removal by hydrogenolysis furnished the MRTX849-HyT conjugates **HY3**–**HY8**. HATU/TEA-mediated coupling proceeded efficiently for the introduction of the adamantyl group, but it was ineffective for the incorporation of the carborane moiety. In this case, coupling using the COMU/DIPEA successfully afforded the desired product. Therefore, in the synthesis of **HY3**–**HY8**, the synthetic procedure was modified to introduce the hydrophobic tag prior to 2-fluoroacrylic constructing the 2-fluoroacrylic moiety. Moreover, the carborane derivatives were synthesized according to our previous studies,^20,30^ using *meta*- and *ortho*-carboranes as the starting materials. Reaction of the anion generated by *n*-BuLi treatment with carbon dioxide afforded derivative **19**; however, further synthesis of *meta*-carborane acetic acid was difficult. Therefore, the more reactive *ortho*-carborane, owing to differences in C–H bond acidity, was employed, and derivative **21** was obtained by reaction with benzyl bromoacetate under *n*-BuLi treatment conditions (Scheme S1). In addition, the coupling of acetylenes with decaborane were carried out under microwave condition to give derivatives **24** and **26** (Scheme S1).

**Scheme 1:**
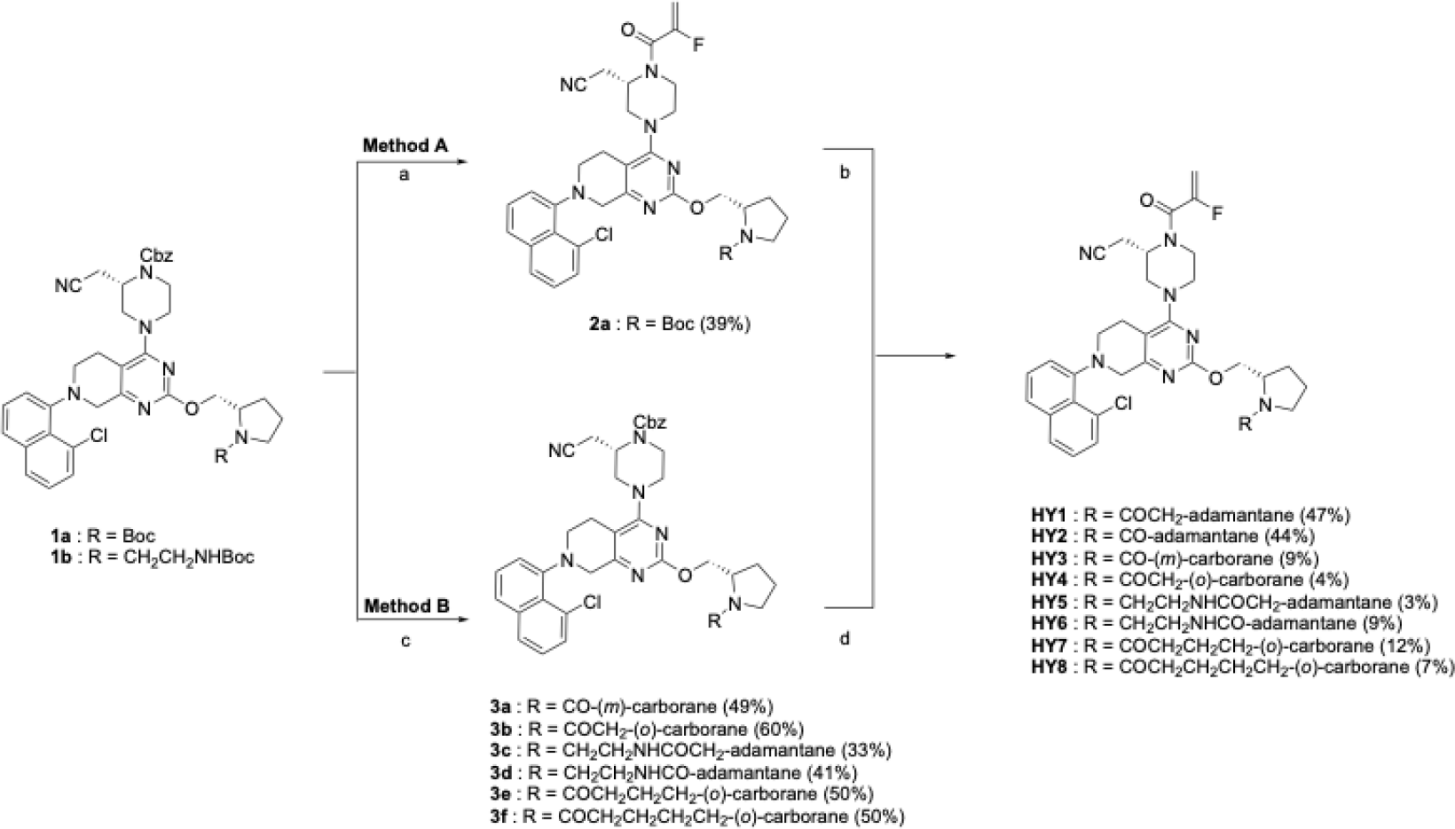
Reagents and Conditions^a^

a (a) (i) H_2_, Pd/C, MeOH, overnight, r.t.; (ii) 2-fluoroacrylic acid, HATU, TEA, DMF, r.t., 1 h; (b) TFA, DCM, r.t., 1 h; (ii) 2-adamantan-1-yl)­acetic acid or adamantane carboxylic acid, HATU, TEA, DMF, 1 h; (c) (i) TFA, DCM, r.t., 1 h; (ii) 2-(adamantan-1-yl)­acetic acid, adamantane carboxylic acid, or carborane-based acids, COMU, DIPEA, 1 h; (d) (i) H_2_, Pd/C, MeOH, overnight, r.t.; (ii) 2-fluoroacrylic acid, HATU, TEA, DMF, r.t., 1 h.

### Biological Evaluation of MRTX849-HyT Conjugates on Selective KRAS^G12C^ Degradation

We first evaluated the cytotoxicity of MRTX849-HyT conjugates (**HY1**, **HY2**, **HY5**, and **HY6** bearing an adamantane tag; **HY3**, **HY4**, **HY7**, and **HY8** bearing a carborane tag) using 3-(4,5-dimethylthiazol-2-yl)-2,5-diphenyltetrazolium bromide (MTT) assays against three representative cancer cell lines: NCI-H23 (KRAS^G12C^-positive), MIA Paca-2 (KRAS^G12C^-positive) and HCT116 (KRAS^G13D^-negative). The results are summarized in Table 1. As expected, parent compound MRTX849 exhibited selectively high cytotoxicity against NCI-H23 cells carrying the KRAS^G12C^ mutation, consistent with its covalent binding to Cys12. Among the HyT conjugates, the adamantane-tagged derivatives (**HY1**, **HY2**, **HY5**, and **HY6**) remained moderately cytotoxic compared to MRTX849 (IC_50_ = 3.3–18.4 μM) to NCI-H23 cells while maintaining partial mutation selectivity on cytotoxicity. In contrast, the carborane-tagged analogues (**HY3**, **HY4**, **HY7**, and **HY8**) showed markedly lower cytotoxicity (IC_50_ > 50 μM) to these three cell lines. All conjugates exhibited minimal cytotoxic effects on the KRAS^G13D^ cell line HCT116, except **HY5** and **HY6**, which exhibited weak nonselective toxicity at higher concentrations (IC_50_ = 16.2 and 8.0 μM, respectively).

**Table 1:** Cell Cytotoxicity of MRTX849-HyT Conjugates (**HY1**–**HY8**) against NCI-H23, MIA Paca-1, and HCT116 Cells^a^ ^,^ ^b^

comp.	IC_50_ (μM, NCI-H23)	IC_50_ (μM, MIA Paca-2)	IC_50_ (μM, HCT116)
**MRXT849**	1.5 ± 0.13	8.0 ± 0.71	8.0 ± 0.31
**HY1**	18.4 ± 0.94	27.1 ± 3.74	>50
**HY2**	3.6 ± 0.08	6.2 ± 0.62	>50
**HY3**	>50	>50	>50
**HY4**	>50	>50	>50
**HY5**	14.6 ± 1.94	8.4 ± 0.94	16.2 ± 1.91
**HY6**	3.3 ± 0.03	5.7 ± 0.32	8.0 ± 0.84
**HY7**	>50	>50	>50
**HY8**	>50	>50	>50

a Half maximal inhibitory concentration (IC_50_) is mean ± SD of a single experiment conducted in triplicate.

b Cytotoxicity was performed using MTT assay against NCI-H23, MIA Paca-2, and HCT116 cells incubated with compounds for 72 h. NCI-H23 and MIA Paca-2: KRAS^G12C^; HCT116: KRAS^G13D^.

Subsequently, all of the conjugates were examined by Western blotting to evaluate their ability to induce KRAS^G12C^ degradation in cells (Figure 2). Among the degraders bearing the classical adamantane HyT, **HY5** exhibited the highest efficacy in reducing the endogenous KRAS^G12C^ level, whereas **HY1** exhibited minimal degradative activity. Because **HY2** and **HY6** caused substantial cytotoxicity, their degradation profiles of these compounds could not be reliably assessed due to loss of total protein, including the internal control α-tubulin. In contrast, the carborane-tagged degraders (**HY3**, **HY4**, **HY7**, and **HY8**) produced moderate to high KRAS^G12C^ degradation, with **HY8** showing the most pronounced effect. Direct comparison of the representative conjugates, **HY5** and **HY8**, revealed that the carborane-based degrader achieved a greater degradation efficacy with lower intrinsic cytotoxicity. Analysis of the correlation between cytotoxicity and KRAS^G12C^ degradation indicates that target degradation does not necessarily result in cytotoxicity. Partial degradation of the target protein (approximately 60% degradation of KRAS^G12C^ upon **HY8** treatment) may be insufficient to induce cytotoxic effects, particularly in KRAS-driven cancer cells that exhibit strong pathway redundancy. Furthermore, the moderate potency of the compounds may limit their functional impact regardless of degradation efficiency or effects on cell viability. The overall results suggested **HY5** and **HY8** were selected as representative compounds for subsequent mechanistic investigation.

**Figure 2:**
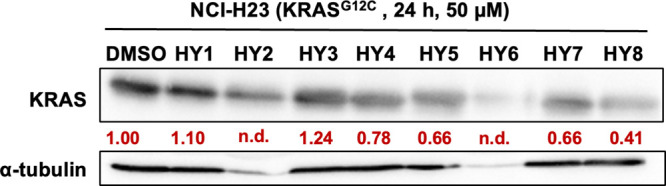
KRAS levels of NCI-H23 cells treated with **HY1**–**HY8** (50 μM) for 24 h. Cell lysates were separated by SDS-PAGE and immunoblotting was performed using anti-KRAS and α-tubulin antibodies. α-Tubulin was used as internal control. Ratios were represented the quantification of KRAS/α-tubulin compared to dimethyl sulfoxide (DMSO) group. n.d.; not detect.

Further analysis demonstrated that both **HY5** and **HY8** degraded endogenous KRAS^G12C^ in a dose- and time-dependent manner in KRAS^G12C^ mutant NCI-H23 and MIA Paca-2 cells (**HY5**: Figures 3A,B and S3A; **HY8**: Figures 3D–F and S3B). This degradation was accompanied by a reduction in the level of phosphorylated-ERK (*p*-ERK), a key downstream effector in the KRAS signaling cascade, which became evident as early as 4 h post-treatment. These results suggested that KRAS^G12C^ depletion efficiently suppressed downstream signaling pathways. In contrast, neither conjugate affected KRAS expression in HCT116 (KRAS^G13D^) cells, verifying that hydrophobic tag conjugation did not abolish the mutation selectivity of MRTX849 (**HY5**: Figure 3C; **HY8**: Figure 3F). Evaluation of MRTX849 alone (Figure S4) showed inhibition of *p*-ERK phosphorylation without affecting the total protein levels of KRAS^G12C^, consistent with its role as a covalent inhibitor rather than a degrader. Together, these results demonstrated the successful development of MRTX849-HyT conjugates as effective KRAS^G12C^ degraders. A slightly extended linker may further improve the exposure of the hydrophobic tag on the protein surface and enhance degradation potency. However, both **HY5** and **HY8** exhibited comparable activity within a similar concentration range (Figure S2), suggesting that the lower cytotoxicity and higher stability imparted by the carborane moiety may indicate broader opportunities for further optimization and applications.

**Figure 3:**
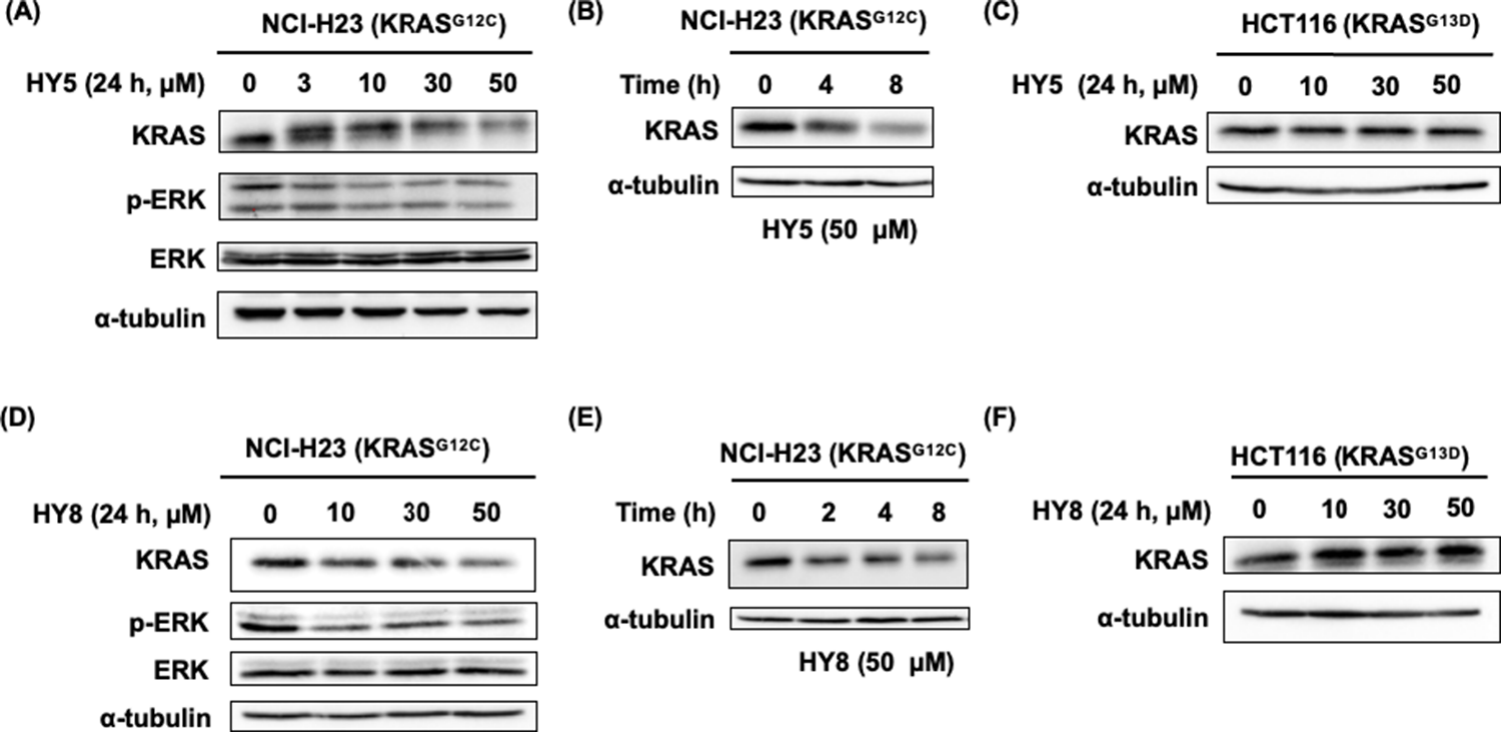
Effects of **HY5** or **HY8** on KRAS protein levels in NCI-H23 (KRAS^G12C^) and HCT116 (KRAS^G13D^) cells. NCI-H23 or HCT116 cells were treated with various concentrations of **HY5** (A–C) or **HY8** (D–F) at the indicated concentrations and times. Cell lysates were separated by SDS-PAGE, and immunoblotting was performed using anti-KRAS, ERK, phospho-ERK, and α-tubulin antibodies. α-Tubulin was used as an internal control.

### Elucidation of the Degradation Mechanism of KRAS by the HyT Degradation System

Inspired by the HyT-induced degradation of KRAS^G12C^, we next investigated the underlying mechanism of action. A competition assay was first performed to verify whether the MRTX849-HyT conjugates occupy the same binding pocket as MRTX849. NCI-H23 cells were pretreated with MRTX849 for 2 h, followed by incubation with **HY5** or **HY8** for an additional 8 h. The abrogation of KRAS degradation upon cotreatment with MRTX849 (Figure 4A,B) supports that both **HY5** and **HY8** covalently engage Cys12, the same site targeted by MRTX849. We then performed immunoprecipitation (IP) experiments to identify the proteins involved in the degradation process. Since HyT degraders can expose a hydrophobic surface on the target proteins and mimic misfolded conformations, they are often recognized by the cellular protein quality control system, including heat shock proteins (HSPs) and the ubiquitin-proteasome machinery. Therefore, the levels of HSP70 and ubiquitin in the complexes were evaluated. As shown in Figure 4C, immunoblotting analysis showed increased levels of HSP70 and ubiquitin after MRTX849-HyTs incubation, suggesting an enhanced interaction of KRAS with HSP70 or ubiquitin. Encouraged by the obvious increase in the ubiquitin level in the **HY8**-treated group, we further performed rescue assays (Figures 4D and S5). Cotreatment with the proteasome inhibitor MG132 blocked KRAS degradation, indicating that the engagement of proteasome pathway is required. In contrast, cotreatment with the lysosomal inhibitor bafilomycin A1 (Baf A1) had no effect on KRAS degradation, suggesting the autophagy/lysosome pathway is not involved in carborane-induced KRAS degradation.

**Figure 4:**
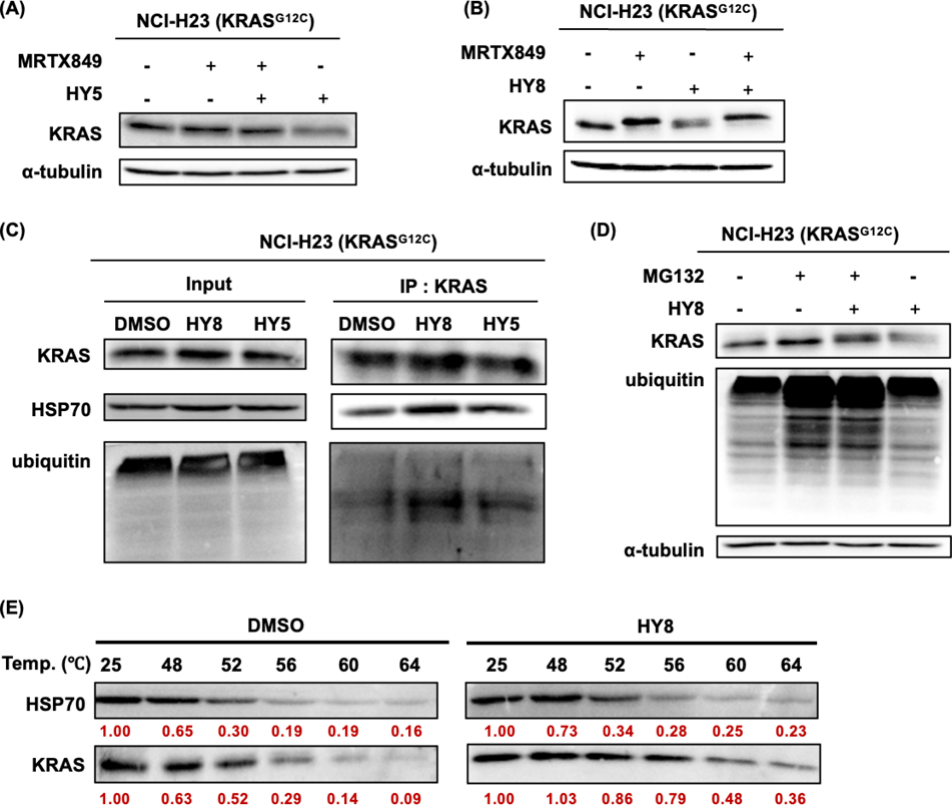
Degradation of endogenous KRAS^G12C^ is via a proteasome pathway. (A and B) NCI-H23 were treated with **HY5** or **HY8** (50 μM) for 8 h after preincubation with MRTX849 (5 μM) for 2 h. (C) NCI-H23 cell lysate was used for immunoprecipitation. After cell lysate was incubated with or without **HY5** or **HY8** (50 μM), the protein complexes were pulled down using an anti-KRAS antibody. (D) NCI-H23 cells were coincubated with **HY8** (50 μM) and MG132 for 8 h. (E) Thermal shift binding assay was used to evaluate the interaction between **HY8** and KRAS, and **HY8** and HSP70. The cell lysate was treated with dimethyl sulfoxide (DMSO) vehicle and **HY8** (50 μM) for 1 h and heated at 25–64 °C for 3 min and kept at 25 °C for a further 3 min. The level of soluble proteins is measured by immunoblotting. The ratio represented the quantification of the treated (48, 52, 56, 60 or 64 °C, heat-increased) group/untreated (room temperature, 25 °C) group.

Finally, to further explore the potential direct interaction between carborane and HSP70, we performed a thermal shift binding assay.^18,31^ This assay assesses ligand binding by monitoring changes in the thermal stability of target proteins as ligand binding can protect target proteins from heat-induced aggregation and/or precipitation. As shown in Figure 4E, treatment of **HY8** did not significantly alter the thermal stability of HSP70, indicating that **HY8** does not directly bind to this chaperone. In contrast, the thermal stability of KRAS increased markedly after incubation with **HY8**, confirming a direct interaction between the degrader and the target protein. Taken together, these findings support a mechanism in which carborane-based HyT degrader **HY8** covalently binds to KRAS, recruits HSP70, and promotes its ubiquitin-proteosome-mediated degradation. Other proteins involved in the degradation process are still under investigation.

### Evaluation of MRTX849-HyTs on Apoptosis

Since persistent KRAS activation promotes uncontrolled cell proliferation, the degradation of KRAS by MRTX849-HyT conjugates was expected to suppress downstream ERK signaling and induce apoptotic responses. We performed Western blotting analyses to monitor apoptosis-related proteins. Active caspase 3 is a widely used biological marker for detecting early apoptosis.^32^ As shown in Figure 5, the appearance of cleaved PARP and caspase-3 indicated that both **HY5** and **HY8** triggered apoptotic signaling. Moreover, the decreased levels of CDK4 and cyclin D1 suggested possible cell-cycle arrest at the G1/S transition, particularly in **HY5**-treated cells. To further validate these observations, we performed flow cytometry analysis using Annexin V-FITC/PI staining in NCI-H23 cells and camptothecin (CPT) was used as a positive control. After 12 h of treatment, **HY5** induced early apoptosis comparable to that in the CPT group (Figure S6A), and apoptotic bodies became evident as early as 4 h after treatment (Figure S6B). These results are consistent with the cytotoxicity data, demonstrating that **HY5** induced a stronger apoptotic response than **HY8**.

**Figure 5:**
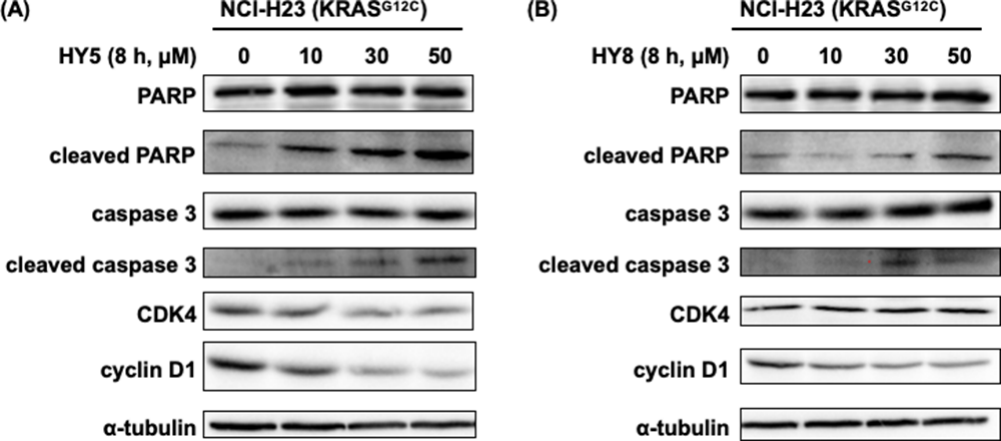
Effects of **HY5**- and **HY8**-induced apoptosis. NCI-H23 (KRAS^G12C^) cells were treated with increasing concentrations of **HY5** (A) or **HY8** (B) for 8 h. Cell lysates were separated by SDS-PAGE, and immunoblotting was performed using antibodies against anti-PARP, cleaved PARP, caspase 3, cleaved caspase 3, CDK4, cyclin D1, and α-tubulin. α-Tubulin was used as an internal control.

## Conclusions

TPD continues to evolve as a powerful strategy to eliminate disease-related proteins previously considered to be undruggable. The most established class of TPD agents, PROTACs, relies on the recruitment of E3 ubiquitin ligase to induce proteasome-mediated degradation through a catalytic-type and recyclable mechanism.^33^ In contrast, the HyT approach employs the bulky hydrophobic tags that mimic the exposed hydrophobic residues on misfolded proteins, thereby activating the cellular proteins' quality-control machinery.^13,14^ Unlike PROTACs, HyT degraders can operate in an E3-ligase-independent manner and engage both ubiquitin-proteasome-dependent and auxiliary degradation pathways. In this study, we developed novel KRAS^G12C^ degraders based on the HyT concept. Inspired by previous studies demonstrating that the conjugation of carborane to BSA induces the exposure of lysine residues and that the conjugation of carborane to a Halo-tag ligand induces intracellular protein degradation, in this study, we synthesized a series of MRTX849-HyT conjugates incorporating adamantane^15,34^ or carborane as a hydrophobic tag. While adamantane is a well-known HyT motif, this represents the first application of carborane in endogenous protein degradation. The resulting conjugates selectively degraded KRAS^G12C^ in a time- and dose-dependent manner, with **HY8** (carborane-tagged) showing potent degradation efficacy and lower cytotoxicity than its adamantane analogue. Mechanistic studies revealed that these degraders covalently bind KRAS, recruit HSP70, and promote polyubiquitination of KRAS followed by proteasomal degradation, leading to suppression of downstream ERK signaling and proapoptotic effects in cells. Carboranes, which occupy a unique chemical space, are characterized by high chemical stability and metabolic resistance, and have recently attracted attention as scaffolds for drug candidates and molecular probes.^35^ In fact, the carborane-based compounds developed in this study indicated no typical aggregation pattern formed in a serum-containing medium (Figure S7), chemical stability during the treatment time (Figures S8 and S9), and exhibited sustained HyT-mediated protein degradation activity (Figures 2 and 3). Although further detailed investigations are required, these initial findings are consistent with previous reports and indicate that carborane derivatives, including the compounds developed here, possess notable biological stability. Overall, these findings establish carborane as a compact and functional hydrophobic tag for targeted protein degradation, expanding the chemical space and mechanistic diversity of TPD beyond classical hydrocarbon-based systems. Although further optimization is required to enhance potency and enable *in vivo* validation, these findings lay the foundation of the future development of boron cluster-based degraders applicable to a broader range of protein targets.

## Experimental Section

### Cell Culture

NCI-H23 cells were cultured in RPMI-1640 medium (FUJIFLIM Wako Pure Chemical Corporation, Japan), and MIA Paca-2 and HCT116 cells were cultured in Dulbecco’s modified Eagle’s medium (DMEM; FUJIFILM Wako Pure Chemical Corporation, Japan). All cells were cultured using each medium supplemented with 10% (v/v) heat-inactivated fetal bovine serum, 100 units/mL penicillin G potassium, and 100 μg/mL streptomycin sulfate (Thermo Fisher Scientific, Inc., USA) at 37 °C in 5% CO_2_.

### MTT Assay

The cells were seeded in a 96-well plate at a density of 3 × 10^3^ cells/well and cultured for 12 h. The cells were treated with each concentration of the compounds. After incubation for 72 h, 0.5 mg/mL MTT was added to each well and incubated for 3 h at 37 °C in 5% CO_2_. Subsequently, the medium was removed, and the formazan product was dissolved in 150 μL of DMSO. The absorbance at 590 nm was recorded using a microplate reader (Infinite F200, Tecan group ltd., Switzerland).

### Western Blot

The cells were seeded in a 6-well plate at a density of 2 × 10^5^ cells/well and cultured overnight. The cells were treated with each concentration of compounds and cultured under the indicated conditions. Subsequently, the cells were washed with cold phosphate-buffered saline (PBS) and lysed using RIPA buffer containing 1% protease inhibitor (FUJIFILM Wako Pure Chemical Corporation, Japan). The cell lysate was centrifuged at 13,000 × *g* for 10 min at 4 °C, and the obtained cell extract was diluted in sample buffer (1 M Tris-HCl (pH = 6.8), 10%(w/w) sodium dodecyl sulfate (SDS), 500 mM dithiothreitol, 50%(v/w) glycerol, 0.5%(w/w) bromophenol blue dye) and boiled for 5 min at 98 °C. The obtained sample was electrophoresed on SDS-polyacrylamide gel and transferred to polyvinylidene difluoride membrane. The membrane was blocked with 5% skim milk in TTBS buffer (Tris-buffered saline containing 0.1% Tween-20) and incubated with each primary antibody (Antibody: TTBS = 1:1000) for 12 h at 4 °C, followed by incubation with each corresponding secondary antibody conjugated with horseradish peroxidase (Antibody: TTBS = 1:5000). Signals were detected with ECL using ImmunoStar LD (FUJIFILM Wako Pure Chemical Corporation, Japan) or Immobilon Forte Western HRP substrates (Merck KGaA, USA). Primary antibodies were listed as follows: anti-KRAS (12063-1-AP, ProteinTech Group Inc., USA), anti-α-tubulin (#013-25033, FUJIFILM Wako Pure Chemical Corporation, Japan), anti-phospho-ERK (#sc-7383, Santa Cruz Biotechnology, Inc., USA), anti-ERK (KAP-MA001, Enzo Life Sciences Inc., USA), anti-ubiquitin (10201-2-AP, ProteinTech Group Inc., USA), anti-HSP70 (#sc-1060-R, Santa Cruz Biotechnology, Inc., USA), anti-PARP (#9542P, Cell Signaling Technology Inc., USA), anti-cleaved PARP (#5625P, Cell Signaling Technology Inc., USA), anti-caspase 3 (#9662P, Cell Signaling Technology Inc., USA), anti-cleaved caspase 3 (#9664P, Cell Signaling Technology Inc., USA), anti-CDK4 (#2906, Cell Signaling Technology Inc., USA), and anti-cyclin D1 (#2926, Cell Signaling Technology Inc., USA) antibodies. Secondary antibodies were listed as follows: anti-rabbit IgG, HRP-linked antibody (SA00001-2, ProteinTech Group Inc., USA) and anti-mouse IgG, HRP-linked antibody (SA00001-1, ProteinTech Group Inc., USA).

### Immunoprecipitation

NCI-H23 cells were seeded in a 60 mm plate at a density of 3 × 10^5^ cells/mL and incubated for 12 h. Cell lysate was extracted using IP lysis buffer (25 mM Tris-HCl (pH = 7.4), 150 mM NaCl, 1 mM EDTA, 1% NP-40, and 5% glycerol) and centrifuged for 15 min at 11,000 × *g* at 4 °C. The obtained cell extract was incubated with each compound at 4 °C for 3 h and anti-KRAS antibody (10 μL/sample) at 4 °C for 4 h, respectively. Then, Protein A/G Plus-agarose (20 μL/sample, #sc-2003, Santa Cruz Biotechnology, Inc., USA) was added to each sample and incubated at 4 °C for 2 h. The agarose beads with immunoprecipitants were collected by centrifugation at 2,500 rpm for 5 min at 4 °C and washed five times with IP lysis buffer. Next, the supernatant was removed, and the obtained pellet was resuspended in sample buffer. After boiling for 5 min at 98 °C, the amount of each protein was analyzed by Western blotting.

### Thermal Shift Binding Assay

NCI-H23 cells were lysed using PBS by seven repeated freeze–thaw cycles under liquid nitrogen conditions, and protein concentrations were determined using the BCA assay. Cell lysates were adjusted to 1 mg/mL protein concentration and treated with either DMSO vehicle or 50 μM of **HY8** for 1 h at 37 °C. These cell lysates were then separated for thermal profiling. Fractions were heated at the indicated temperatures for 3 min and then at 25 °C for 3 min using a thermal heater (T100, Bio-Rad). Samples were centrifuged at 20,000 × *g* for 15 min to remove protein aggregates. The supernatants were collected for Western blotting.

### Cell Apoptosis

NCI-H23 cells were seeded in 35 mm dishes and incubated overnight. Then, cells were treated with indicated compounds for different time. Then, the cells were collected and stained by Annexin V-FITC/PI kit (#ab14085, Abcam Ltd., UK) according to the manufacturer’s instructions. The fluorescence signals of FITC and PI were recorded by flow cytometer (BD FACSMelody, BD Biosciences).

### General Information: Compounds Synthesis

NMR spectra were recorded on a BrukerBioSpin AVANCE III (500 and 400 MHz for ^1^H, 100 and 125 MHz for ^13^C) instrument in the solvent indicated as following. Chemical shifts are reported in parts per million (ppm) relative to the CDCl_3_ or DMSO-*d*_6_ of the standard. Multiplicities are reported using the following abbreviations: s, singlet; d, doublet; dd, doublet of doublets; t, triplet; q, quartet; m, multiplet; brs, broad; *J*, coupling constants in Hertz (Hz). High-resolution mass spectra (HRMS) were recorded on a Bruker electrospray ionization (ESI)–time-of-flight (TOF)–mass spectrometer (micrOTOF II, Bruker). Analytical thin-layer chromatography (TLC) was performed on a glass plate of silica gel 60 GF254 (Merck). Silica gel (Fuji Silysia, CHROMATOREX PSQ 60B, 50–200 μm) was used for column chromatography. All chemicals and reagents for biological experiments were obtained from commercial sources and used without further purification. Purity of all assayed compounds was determined by a reverse-phase high performance liquid chromatography (HPLC) system (JASCO Corporation, Japan) equipped with a C18 column (Finepak SIL C18S (4.6 × 150 mm), JASCO Corporation, Japan). All compounds were detected at 254 nm using isocratic solvent (50% H_2_O: 50% acetonitrile (MeCN) followed by a linear gradient to 100% MeCN in 20 min or 90% H_2_O: 10% MeCN followed by a linear gradient to 100% MeCN in 20 min, flow rate 1.0 mL/min). No unexpected or unusually high safety hazards were encountered in all organic synthesis experiments.

### Synthesis of **HY1**

To a solution of compound **1a** (390.0 mg, 0.52 mmol) in methanol (MeOH; 10.0 mL), Pd/C (150.0 mg, 30 wt %) was added under an argon atmosphere. The suspension was exchanged from argon to H_2_ atmosphere. After being stirred at room temperature overnight, the Pd/C catalyst was filtered off and the resulting solution was concentrated under vacuo to give the desired intermediate (167.0 mg, 0.27 mmol, yield 52%), which was used in the next step without further purification.

To a solution of above intermediate (167.0 mg, 0.27 mmol, 1.0 equiv), 2-fluoroacrylic acid (49.0 mg, 0.54 mmol, 2.0 equiv) and 1-((dimethylamino)­(dimethyliminio)­methyl)-1*H*-[1,2,3]­triazolo­[4,5-*b*]­pyridine 3-oxide hexafluorophosphate (HATU; 154.0 mg, 0.41 mmol, 1.5 equiv) in *N*,*N*-dimethylformamide (DMF; 5.0 mL), triethylamine (TEA; 150.0 μL, 1.1 mmol, 4.0 equiv) were added slowly. After being stirred for 1 h at room temperature, the reaction mixture was extracted with ethyl acetate (EtOAc), washed with brine, dried over MgSO_4_, and concentrated under vacuo. The obtained residue was purified by column chromatography on silica gel (EtOAc: hexane = 60:40) to give the second intermediate **2a** (141.0 mg, 0.2 mmol, yield 75%).

To a solution of the second intermediate (15.0 mg, 0.036 mmol, 1.0 equiv) in dichloromethane (DCM; 2.0 mL), trifluoracetic acid (TFA; 0.1 mL, 0.05 v/v) was added under an argon atmosphere at room temperature. After being stirred for 1 h at room temperature, the reaction mixture was extracted with EtOAc, washed with NaHCO_3_ and brine, dried over MgSO_4_, and concentrated under vacuo. The obtained residue was used for the next step without further purification. To a solution of the above crude intermediate dissolved in DMF (1.5 mL), adamantaneacetic acid (25.0 mg), HATU (60.0 mg), and TEA (0.08 mL) were added at room temperature. After being stirred for 1 h, the reaction mixture was extracted with EtOAc, washed with brine, dried over MgSO_4_, and concentrated under vacuum. The obtained residue was purified by column chromatography on silica gel (EtOAc: hexane = 80:20) to give the desired compound **HY1** (13.0 mg, yield 47% for two steps). Purity: 98% (retention time: 15.85 min). ^1^H NMR (500 MHz, CDCl_3_) δ 7.76–7.74 (1H, m), 7.62–7.59 (1H, m), 7.53–7.50 (1H, m), 7.46–7.41 (1H, q, *J* = 7.8 Hz), 7.35–7.31 (1H, q, *J* = 5.7 Hz), 7.23–7.19 (1H, m), 5.45–5.35 (1H, m), 5.25–5.21 (1H, m), 4.53–4.37 (3H, m), 4.30–4.16 (1H, m), 4.10–4.06 (1H, m), 3.90–3.80 (1H, q, *J* = 15 Hz), 3.56–3.55 (2H, m), 3.49–3.48 (1H, m), 3.24–3.21 (2H, m), 3.14–3.11 (2H, m), 2.88–2.86 (2H, m), 2.58–2.55 (1H, m), 2.13–2.00 (4H, m), 1.94–1.92 (5H, m), 1.66–1.57 (15H, m). ^13^C NMR (125 MHz, CDCl_3_) δ 170.5, 166.1, 162.7, 148.4, 137.3, 129.7, 128.2, 126.4, 125.9, 125.6, 125.0, 118.7, 118.5, 65.9, 58.9, 55.6, 50.5, 50.4, 48.4, 47.5, 45.4, 42.7, 42.6, 36.8, 33.7, 33.6, 31.6, 29.7, 28.7, 27.4, 26.0, 24.3, 22.6, 14.1. HRMS (ESI, positive) for C_43_H_49_O_3_ClFN_7_ (*m*/*z*): calculated 766.3642 (M + H)^+^, found 766.3644.

### Synthesis of **HY2**

Compound **HY2** was synthesized by the same method as that for compound **HY1**, with adamantaneacetic acid replaced by adamantane carboxylic acid. Compound **HY2** (12.0 mg, yield 44% for two steps) was obtained. Purity: 97% (Retention time: 16.06 min). ^1^H NMR (500 MHz, CDCl_3_) δ 7.61–7.59 (1H, d, *J* = 7.9 Hz), 7.52–7.50 (1H, q, *J* = 6.4 Hz), 7.46–7.40 (1H, m), 7.34–7.31 (1H, t, *J* = 7.9 Hz), 7.24–7.18 (1H, dd, *J* = 13.1 Hz, 7.4 Hz), 5.45–5.35 (1H, m), 5.26–5.22 (1H, m), 4.56 (2H, br), 4.43–4.38 (1H, q, *J* = 13.9 Hz), 4.28–4.26 (1H, m), 4.17 (1H, br), 4.07–4.06 (1H, m), 3.96–3.80 (3H, m), 3.65–3.54 (3H, m), 3.25 (2H, br), 3.17–3.08 (2H, m), 2.91–2.87 (2H, m), 2.59–2.54 (1H, m), 2.02–1.85 (15H, m), 1.71–1.69 (5H, m). ^13^C NMR (125 MHz, CDCl_3_) δ: 176.3, 166.4, 166.1, 162.7, 157.9, 155.7, 148.5, 148.1, 137.4, 137.3, 130.1, 130.0, 129.6, 128.2, 126.4, 126.0, 125.5, 124.8, 118.6, 118.5, 116.8, 66.3, 58.9, 57.6, 50.2, 47.9, 41.9, 38.3, 36.4, 29.7, 28.4, 26.2, 25.7. HRMS (ESI, positive) for C_42_H_47_O_3_ClFN_7_ (*m*/*z*): calculated 752.3486 (M + H)^+^, found 752.3481.

### Synthesis of **HY3**

To a solution of compound **1a** (207 mg, 0.27 mmol, 1.0 equiv) in DCM (2 mL), TFA (0.5 mL) was added under an argon atmosphere at room temperature. After being stirred for 1 h at room temperature, the reaction mixture was poured into NaHCO_3_, extracted with EtOAc, and washed with brine. The combined organic layer was dried using MgSO_4_ and concentrated under vacuum. The residue was used for the next step without further purification. To a solution of the above crude intermediate dissolved in DMF (2.0 mL), carboxyl *meta*-carborane (52.0 mg, 0.27 mmol, 1.2 equiv), ethyl 2-cyano-2-((dimethyliminio)­(morpholino)­methyloxyimino)­acetate hexafluorophosphate (COMU; 108.0 mg, 0.25 mmol, 1.1 equiv), and *N*,*N*-diisopropylethylamine (DIEA; 81.0 μL, 0.46 mmol, 2.0 equiv) were added at room temperature. After the reaction mixture was stirred for 30 min, it was extracted with EtOAc, washed with brine, dried with MgSO_4_, and concentrated under vacuum. The residue was purified by column chromatography on silica gel (acetone:hexane = 30:70) to give the desired intermediate **3a** (110.0 mg, 0.13 mmol, yield 49% for two steps).

To a solution of the above intermediate (110.0 mg, 0.13 mmol) in MeOH (4.0 mL), Pd/C (55.0 mg, 50 wt %) was added under an argon atmosphere. The suspension was exchanged from argon to H_2_ atmosphere. After completion, the Pd/C catalyst was filtered off, and the resulting solution was concentrated to give the desired intermediate, which was used in the next step without further purification. To a solution of the above intermediate mentioned above dissolved in DMF (2.0 mL) were added 2-fluoroacrylic acid (23.4 mg, 0.26 mmol), HATU (74.1 mg, 0.19 mmol), and TEA (72.0 μL, 0.52 mmol) under an argon atmosphere. After being stirred for 30 min under room temperature, the reaction mixture was extracted with EtOAc, washed with brine, dried over with MgSO_4_ and concentrated under vacuo. The obtained residue was purified by preparative TLC (EtOAc: hexane = 50:50) to give the desired compound **HY3** (9.0 mg, yield 9% for two steps). Purity: 95% (retention time: 19.5 min). ^1^H NMR (500 MHz, CDCl_3_) δ 7.74 (1H, d, *J* = 7.8 Hz), 7.60 (1H, d, *J* = 8.0 Hz), 7.52–7.50 (1H, m), 7.45–7.42 (1H, m), 7.34–7.30 (1H, t, *J* = 7.9 Hz), 7.22–7.21 (1H, m), 4.60–4.43 (2H, m), 4.42–4.37 (1H, m), 4.24–4.20 (2H, m), 4.05–4.02 (1H, m), 3.89–3.55 (8H, m), 3.39–3.17 (3H, m), 3.12–2.80 (8H, m), 2.63–2.04 (12H, m). ^13^C NMR (125 MHz, CDCl_3_) δ: 137.4, 130.1, 129.6, 128.2, 126.4, 125.9, 125.6, 124.9, 118.6, 70.0, 65.0, 59.6, 58.9, 54.6, 51.9, 51.3, 48.3, 45.2, 29.7. HRMS (ESI, positive) for C_34_H_43_B_10_O_3_ClFN_7_ (*m*/*z*): calculated 762.4103 (M + H)^+^, found 762.4102.

### Synthesis of **HY4**

Compound **HY4** was synthesized by the same method as that for compound **HY3**, with carboxyl *meta*-carborane replaced by *ortho*-carborane acetic acid. Compound **HY4** (5.5 mg, yield 4% for two steps). Purity: 99% (retention time: 18.82 min). ^1^H NMR (500 MHz, CDCl_3_) δ 7.77–7.74 (1H, m), 7.65–7.60 (1H, m), 7.54–7.51 (1H, t, *J* = 7.7 Hz), 7.47–7.41 (1H, m), 7.36–7.31 (1H, q, *J* = 4.3 Hz), 7.21–7.19 (1H, m), 5.46–5.24 (1H, m), 4.90–4.71 (1H, m), 4.43–4.29 (3H, m), 4.12–4.07 (1H, m), 3.98–3.59 (4H, m), 3.74 (1H, s), 3.47–3.31 (3H, m), 3.21–3.12 (4H, m), 2.98–1.98 (8H, m). ^13^C NMR (125 MHz, CDCl_3_) δ 166.0, 165.6, 162.4, 137.3, 130.0, 129.9, 129.7, 129.0, 128.3, 126.4, 125.9, 125.7, 125.6, 118.8, 118.6, 58.7, 58.4, 56.4, 56.3, 48.3, 45.9, 40.9, 40.2, 40.1, 31.9, 29.7, 27.5, 24.1, 24.0, 14.1. HRMS (ESI, positive) for C_35_H_45_B_10_O_3_ClFN_7_ (*m*/*z*): calculated 776.4260 (M + H)^+^, found 776.4262.

### Synthesis of **HY5**

To a solution of compound **1b** (215 mg, 0.27 mmol, 1.0 equiv) in DCM (2 mL), TFA (0.5 mL) was added under argon atmosphere at room temperature. After being stirred for 1 h, the reaction mixture was poured into NaHCO_3_, extracted with EtOAc, and washed with brine. The combined organic layer was dried using MgSO_4_ and concentrated under vacuum. The residue was used for the next step without further purification. To a solution of the above crude intermediate dissolved in DMF (2.0 mL), adamantaneacetic acid (52.0 mg, 0.27 mmol, 1.2 equiv), COMU (108.0 mg, 0.25 mmol, 1.1 equiv), and DIEA (81.0 μL, 0.46 mmol, 2.0 equiv) were added at room temperature. After the reaction mixture was stirred for 30 min, it was extracted with EtOAc, washed with brine, dried using MgSO_4_, and concentrated under vacuum. The residue was purified by column chromatography on silica gel (acetone: hexane = 30:70) to give the desired intermediate (100.0 mg, 0.11 mmol, 33% for two steps).

To a solution of the above intermediate (77.0 mg, 0.13 mmol) in MeOH (4.0 mL), Pd/C (39.0 mg, 50 wt %) was added under an argon atmosphere. The suspension was exchanged from argon to H_2_ atmosphere. After completion, the catalyst was filtered off, and the resulting solution was concentrated to give the desired intermediate, which was used for the next step without further purification. To a solution of the intermediate mentioned above dissolved in DMF (2.0 mL), 2-fluoroacrylic acid (23.4 mg, 0.26 mmol), HATU (74.1 mg, 0.19 mmol), and TEA (72.0 μL, 0.52 mmol) were added under argon atmosphere. After being stirred for 30 min under room temperature, the reaction mixture was extracted with EtOAc, washed with brine, dried using MgSO_4_ and concentrated under vacuum. The obtained residue was purified by preparative TLC (EtOAc: hexane = 50:50) to give the desired compound **HY5** (2.6 mg, yield 3% for two steps). Purity: 96% (Retention time: 15.3 min). ^1^H NMR (500 MHz, CDCl_3_) δ 7.75 (1H, d, *J* = 10.0 Hz), 7.63–7.60 (1H, t, *J* = 10.0 Hz), 7.53–7.51 (1H, m), 7.35–7.31 (1H, t, *J* = 7.7 Hz), 7.21–7.20 (1H, m), 6.13 (1H, brs), 5.48–5.36 (1H, m), 5–27–5.22 (1H, m), 4.44–4.38 (1H, dd, *J* = 12.2 Hz, 20.0 Hz), 4.28–4.21 (2H, m), 4.17–4.01 (3H, m), 3.92–3.37 (3H, m), 3.64 (3H, m), 3.49–3.44 (4H, m), 3.23–3.11 (5H, m), 1.99–2.80 (5H, 2.61–2.56 (2H, m), 2.32–2.38 (2H, m), 1.60 (15H, m). ^13^C NMR (125 MHz, CDCl_3_) δ: 171.0, 137.2, 129.8, 128.2, 126.4, 125.6, 101.3, 62.1, 59.3, 54.2, 51.7, 50.4, 42.6, 38.1, 34.7, 32.6, 31.6, 29.7, 28.6, 28.4, 22.7, 14.1. HRMS (ESI, positive) for C_45_H_54_O_3_ClFN_8_ (*m*/*z*): calculated 809.4064 (M + H)^+^, found 809.4063.

### Synthesis of **HY6**

Compound **HY6** was synthesized by the same method as that for compound **HY5**, with admantaneacetic acid replaced by adamantane carboxylic acid. **HY6** (6.7 mg, yield 9% for two steps). Purity: 96% (retention time: 15.28 min). ^1^H NMR (500 MHz, DMSO-*d*_6_) δ 7. (1H, m), 7.63–7.60 (1H, t, *J* = 7.9 Hz), 7.51 (1H, d, *J* = 7.4 Hz), 7.47–7.41 (1H, m), 7.35–7.31 (1H, t, *J* = 7.6 Hz), 7.23–7.19 (1H, m), 6.22 (1H, brs), 5.46–5.36 (1H, m), 5.27–5.23 (1H, dd, *J* = 3.4 Hz, 16.9 Hz), 4.42–4.36 (1H, m), 4.25–4.00 (5H, m), 3.89–3.85 (1H, m), 3.79–3.74 (1H, m), 3.17–3.09 (5H, m), 2.98–2.95 (3H, m), 2.87–2.84 (2H, m), 2.60–2.58 (2H, m), 2.32 (1H, brs), 1.91–1.91 (3H, m), 1.67–1.60 (15H, m). ^13^C NMR (125 MHz, DMSO-*d*_6_) δ 181.9, 172.2, 171.3, 169.4, 167.3, 161.7, 159.7, 153.4, 142.3, 136.9, 134.7, 133.9, 131.9, 131.2, 130.3, 124.1, 123.5, 113.8, 105.2, 77.6, 74.9, 74.3, 72.6, 68.1, 66.9, 65.4, 60.0, 59.2, 58.8, 55.0, 47.0, 41.3, 36.6, 34.9, 34.4, 33.5, 32.8, 28.3, 27.7, 27.3. HRMS (ESI, positive) for C_44_H_52_O_3_ClFN_8_ (*m*/*z*): calculated 795.3908 (M + H)^+^, found 795.3908.

### Synthesis of **HY7**

Compound **HY7** was synthesized by the same method as that for compound **HY3**, with carboxyl *meta*-carborane replaced by 1-(3-carboxypropyl)-*o*-carborane. Compound **HY7** (6.7 mg, yield 12% for two steps). Purity: 97% (retention time: 18.05 min). ^1^H NMR (400 MHz, CDCl_3_) δ 7.75 (1H, d, *J* = 8.5 Hz), 7.62–7.60 (1H, m), 7.52–7.50 (1H, m), 7.47–7.43 (1H, m), 7.36–7.31 (1H, m), 7.23–7.19 (1H, m), 5.47–5.35 (1H, m), 5.25 (1H, m), 4.84 (1H, brs), 4.56–4.38 (3H, m), 4.21–4.01 (3H, m), 3.89–3.79 (1H, m), 3.66–3.39 (6H, m), 3.24–3.13 (4H, m), 2.85 (2H, brs), 2.74–1.66 (21H, m). ^13^C NMR (100 MHz, CDCl_3_) δ: 171.5, 171.2, 166.2, 162.6, 162.2, 157.7, 155.6, 148.4, 137.3, 129.8, 128.4, 126.4, 125.6, 125.0, 118.7, 108.6, 75.1, 66.9, 65.9, 61.1, 59.0, 56.4, 55.8, 50.3, 47.3, 45.9, 37.9, 34.3, 33.3, 31.8, 29.6, 28.9, 27.4, 26.3, 24.1, 23.8, 21.6, 14.2. HRMS (ESI, positive) for C_37_H_49_B_10_O_3_ClFN_7_ (*m*/*z*): calculated 804.4573 (M + H)^+^, found 804.4572.

### Synthesis of **HY8**

Compound **HY8** was synthesized by the same method as that for compound **HY3**, with carboxyl *meta*-carborane replaced by 1-(4-carboxyburyl)-*o*-carborane. **HY8** (18.0 mg, yield 7% for two steps). Purity: 98% (retention time: 26 min). ^1^H NMR (500 MHz, CDCl_3_) δ 7.77 (1H, d, *J* = 10.1 Hz), 7.64–7.62 (1H, m), 7.54–7.52 (1H, m), 7.49–7.45 (1H, q, *J* = 10.0 Hz), 7.39–7.33 (1H, q, *J* = 9.4 Hz), 7.25–7.21 (1H, m), 5.49–5.37 (1H, m), 5.29–5.25 (1H, m), 4.86 (1H, brs), 4.56–4.38 (3H, m), 4.25–4.05 (3H, m), 3.91–3.82 (1H, m), 3.65–3.40 (6H, m), 3.26–3.14 (4H, m), 2.90–2.85 (1H, m), 2.82 (1H, s), 2.26–1.50 (23H, m, containing H on carborane). ^13^C NMR (125 MHz, CDCl_3_) δ 171.1, 166.5, 166.3, 162.6, 148.4, 148.1, 137.4, 129.7, 128.2, 126.4, 125.6, 125.1, 125.0, 118.7, 116.8, 108.6, 101,4, 75.2, 66.0, 61.1, 59.0, 58.7, 56.2, 55.9, 50.4, 47.4, 45.7, 38.6, 37.9, 34.1, 28.9, 27,4, 26,1, 25.9, 24.1, 23.9, 21.7. HRMS (ESI, positive) for C_38_H_51_B_10_O_3_ClFN_7_ (*m*/*z*): 817.4797 calculated (M + H)^+^, found 817.4799.

## Supplementary Material



## Notes

X-ray crystallography analysis; inhibitory effects on KRAS protein level; effects of cotreatment of **HY8** and bafilomycin A1; effects of **HY5** and **HY8** on apoptosis pathway and particle size distribution; ESI-MS for HY8; HPLC for **HY8**, as well as chemical methods and compound characterization (^1^H NMR, ^13^C NMR, and purity) (PDF)

## ABBREVIATIONS

AR: androgen receptor
Baf A1: bafilomycin A1
Cbz: benzyloxycarbonyl
BCL-xL: B cell lymphoma extra large
Boc: *tert*-butoxycarbonyl
BTK: Bruton’s tyrosine kinases
CPT: camptothecin
CRBN: cereblon
COMU: ethyl 2-cyano-2-((dimethyliminio)­(morpholino)­methyloxyimino)­acetate hexafluorophosphate
DCM: dichloromethane
DIEA: *N*,*N*-diisopropylethylamine
DMF: *N*,*N*-dimethylformamide
DMSO: dimethyl sulfoxide
ER: estrogen receptor
EtOAc: ethyl acetate
HATU: 1-((dimethylamino)­(dimethyliminio)­methyl)-1*H*-[1,2,3]­triazolo­[4,5-*b*]­pyridine 3-oxide hexafluorophosphate
HPLC: high performance liquid chromatography
HRMS: high-resolution mass spectra
HSP: heat shock protein
HyT: hydrophobic tag
IC_50_: half maximal inhibitory concentration
IP: immunoprecipitation
KRAS: the Kirsten rat sarcoma viral oncogene homologue
MeCN: acetonitrile
MeOH: methanol
MTT: (3-(4,5-dimethylthiazol-2-yl)-2,5-diphenyltetrazolium bromide)
PBS: phosphate buffered saline
POI: protein of interest
PROTAC: proteolysis targeting chimera
SDS: sodium dodecyl sulfate
TEA: triethylamine
TFA: trifluoracetic acid
TLC: thin-layer chromatography
TPD: targeted protein degradation
3D: three-dimensional
