# Supplementary material for: Carborane Hydrophobic Tags Drive Selective Degradation of Endogenous KRASG12C via HSP70–Ubiquitin–Proteasome Pathway
Source: ACS Bio Med Chem Au. 2026 Mar 10;6(3):272–81. doi: 10.1021/acsbiomedchemau.6c00004 (PMC13281000; doi:10.1021/acsbiomedchemau.6c00004)
Supplement: Supplementary file 1 [file bg6c00004_si_001.pdf]

Carborane Hydrophobic Tags Drive Selective Degradation of Endogenous KRAS^{G12C} *via* HSP70–Ubiquitin–Proteasome Pathway

Yujie Shao [a], *Kazuki Miura* [a,b], *Hiroyuki Nakamura* [a,b,*]

[a] School of Life Science and Technology, Institute of Science Tokyo, 4259 Nagatsuta-cho Midori-ku, Yokohama, 226-8501, Japan.

[b] Laboratory for Chemistry and Life Science, Institute of Integrated Research, Institute of Science Tokyo, 4259 Nagatsuta-cho Midori-ku, Yokohama, 226-8501, Japan.

*** Corresponding Author**

Hiroyuki Nakamura; Laboratory for Chemistry and Life Science, Institute of Integrated Research, Institute of Science Tokyo, Yokohama 226-8501, Japan; Phone: +81-(0)45-924-5244; E-mail: hiro@cls.iir.isct.ac.jp

List of contents

1. Supporting Data

Figure S1. The X-ray crystallography analysis of MRTX849 bound to KRAS^{G12C}. S2

Figure S2. Inhibitory effects on KRAS protein level induced by **HY5** and **HY8**. S3

Figure S3. Inhibitory effects on KRAS signaling pathway induced by **HY5** and **HY8**. S4

Figure S4. Inhibitory effects on KRAS signaling pathway induced by MRTX849. S4

Figure S5. Effects of co-treatment of **HY8** and bafilomycin A1 on KRAS protein level. S5

Figure S6. Effects of **HY5** and **HY8** on apoptosis pathway. S5

Figure S7. Effect of **HY8** on particle size distribution. S6

Figure S8. Representative ESI-MS for **HY8** incubated in PBS solution. S7

Figure S9. Representative HPLC for **HY8** incubated in PBS solution. S10

Scheme S1. Synthetic schemes of the intermediates. S11

2. Synthetic protocol S12

3. ^{1}H, ^{13}C NMR and high-performance liquid chromatography (HPLC) spectra S21

4. References S42

1. Supporting Data

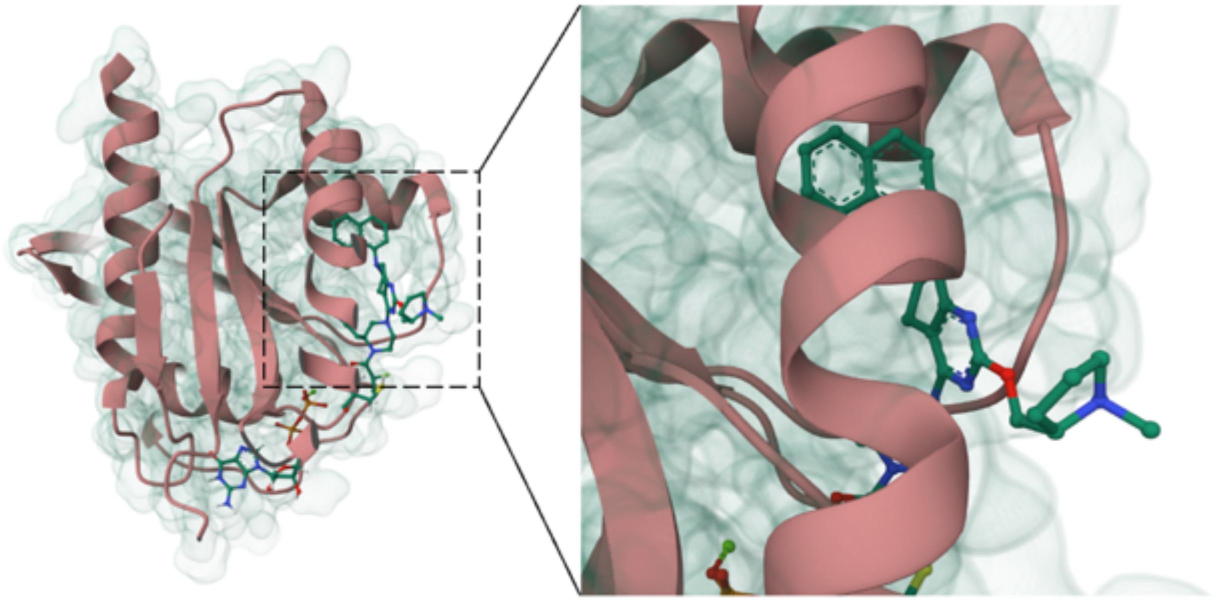

Figure S1. The X-ray crystallography analysis of MRTX849 bound to $KRAS^{G12C}$ (PDB ID: 6TU0).

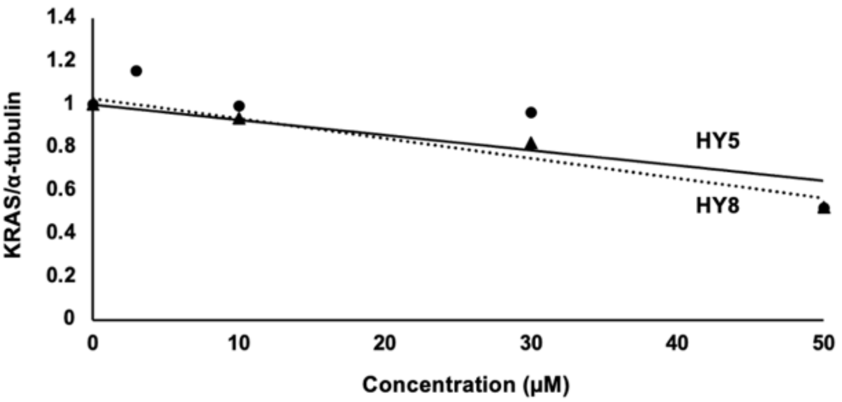

Figure S2. Inhibitory effects on KRAS protein level induced by **HY5** and **HY8**. NCI-H23 cells were treated with various concentration of **HY5** or **HY8** for 24 h. Cell lysates were separated by SDS-PAGE and immunoblotting was performed. Each control was defined as 1.00.

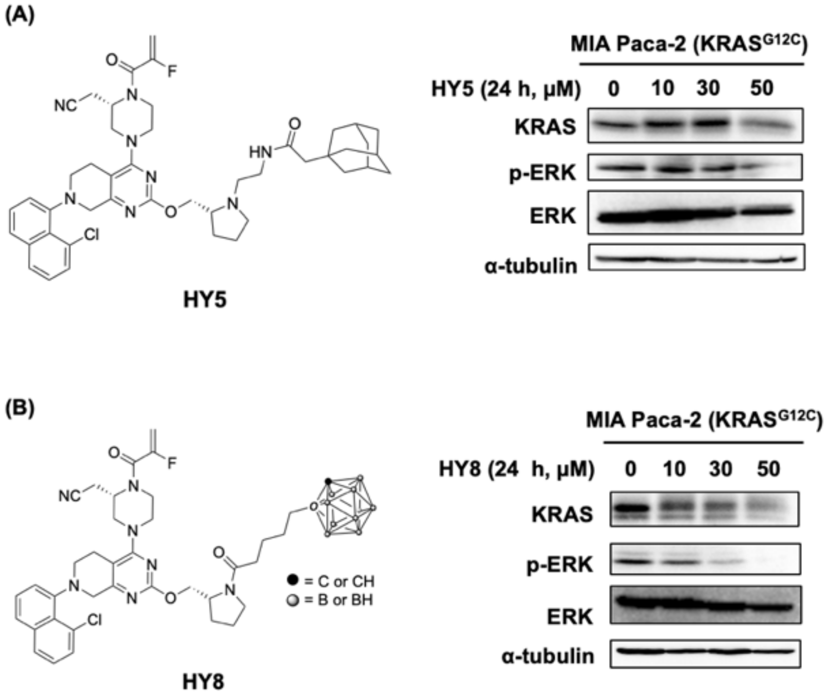

Figure S3. Effects of **HY5** or **HY8** on KRAS protein levels in MIA paca-2 ($KRAS^{G12C}$) cells. MIA Paca-2 cells were incubated with various concentrations of **HY5** or of **HY8** for 24 h. Cell lysates were separated by SDS-PAGE and immunoblotting was performed using anti-KRAS, ERK, phospho-ERK (Tyr 204) and α-tubulin antibodies. α-Tubulin was used as internal control.

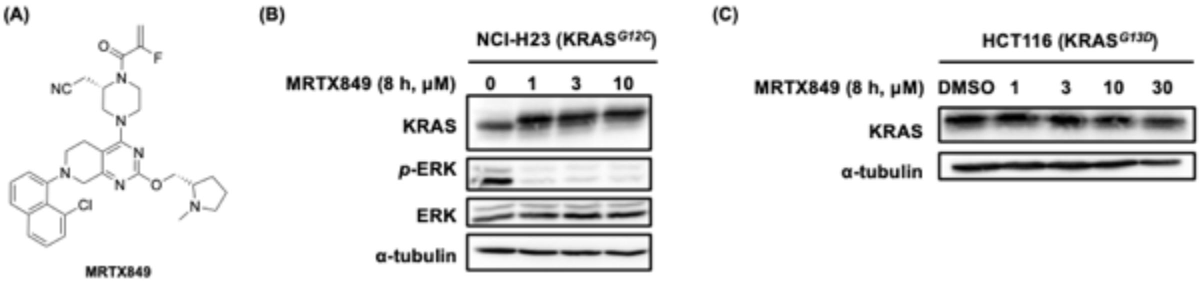

Figure S4. Effects of MRTX849 on KRAS signaling pathway. NCI-H23 and HCT116 cells were incubated with MRTX849 as indicated treatment conditions. Cell lysates were separated by SDS-PAGE and immunoblotting was performed using anti-KRAS, ERK, phospho-ERK (Tyr 204) and α-tubulin antibodies. α-Tubulin was used as internal control.

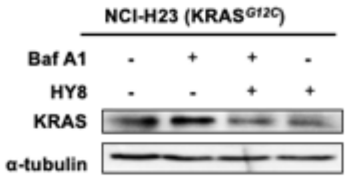

Figure S5. Effects of co-treatment of **HY8** and bafilomycin A1 on KRAS protein level. NCI-H23 cells were co-incubated with **HY8** (50 μM) and bafilomycin A1 (5 μM) for 8 h. Cell lysates were separated by SDS-PAGE and immunoblotting was performed using anti-KRAS and α-tubulin antibodies. α-Tubulin was used as internal control.

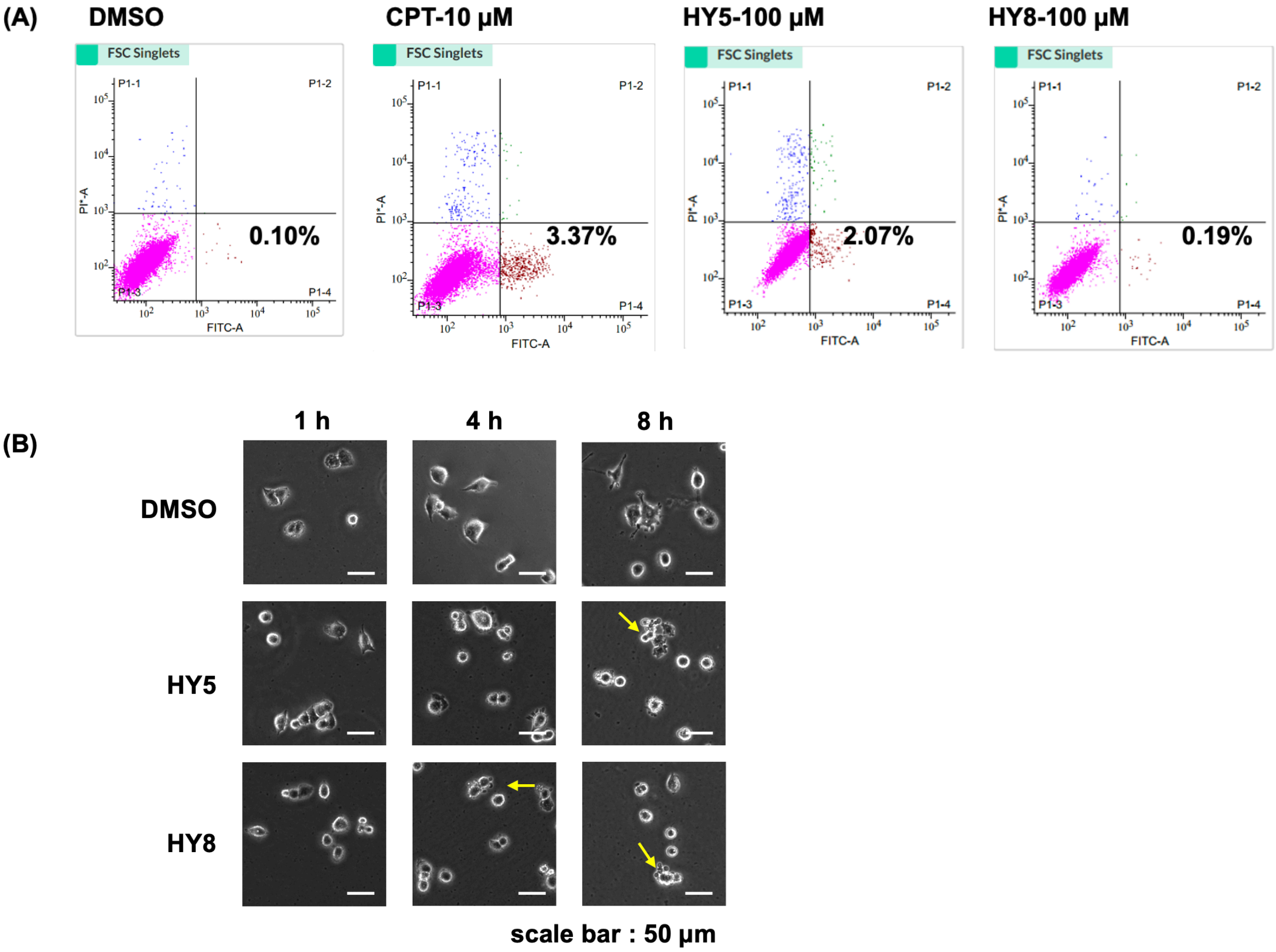

Figure S6. Effects of **HY5** and **HY8** on apoptosis pathway. (A) NCI-H23 cells were treated with **HY5** (100 μM), **HY8** (100 μM) and camptothecin (CPT, 10 μM) for 12 h. Apoptotic cells were stained by Annexin V-Fluorescein isothiocyanate (FITC)/propidium iodide (PI) kit as instructions. Then, the stained cells were detected using flow cytometry. (B) NCI-H23 cells were treated with **HY5** (50 μM) and **HY8** (50 μM) for indicated time and the induced apoptotic bodies were imaged by phase contrast microscope.

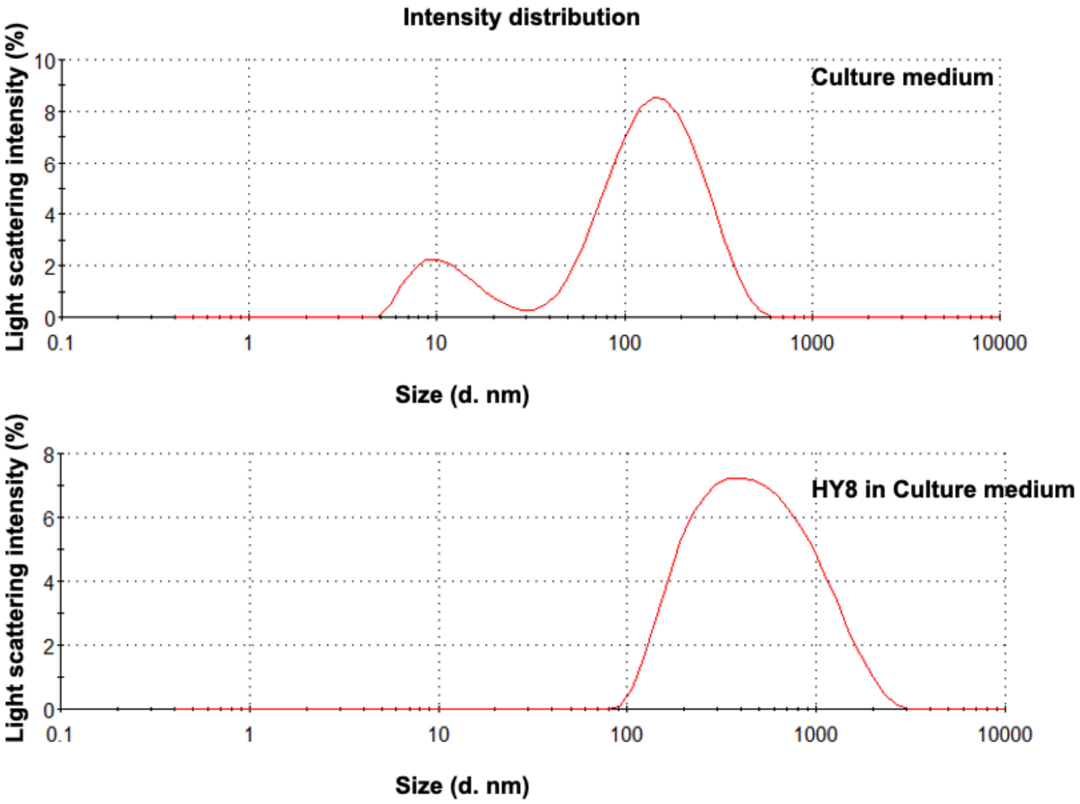

Figure S7. Effect of **HY8** on particle size distribution. The particle size distribution was measured by dynamic light scattering (DLS) analysis. **HY8** (50 μM) was dissolved in 1 mL of PBS. After reacting at room temperature for 1 h, the sample was loaded in borosilicate glass tubes with a height of 7.5 cm and an external diameter of 8 mm. The sample tubes were inserted in the sample compartment of the apparatus, using water (n = 0.890) as index matching fluid. Then, DLS measurements were performed at 25 °C.

(A) HY8 (50 µM) in PBS (containing 10% FBS)

0 h

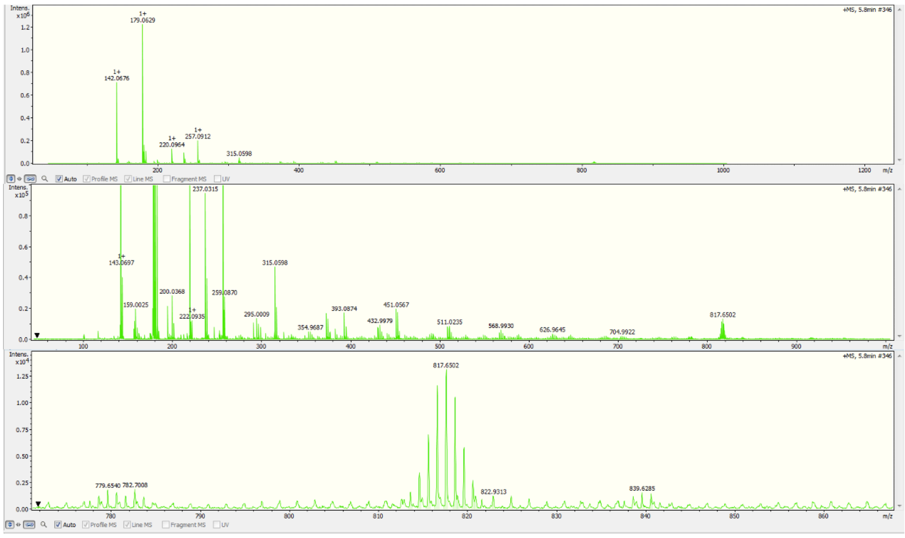

12 h

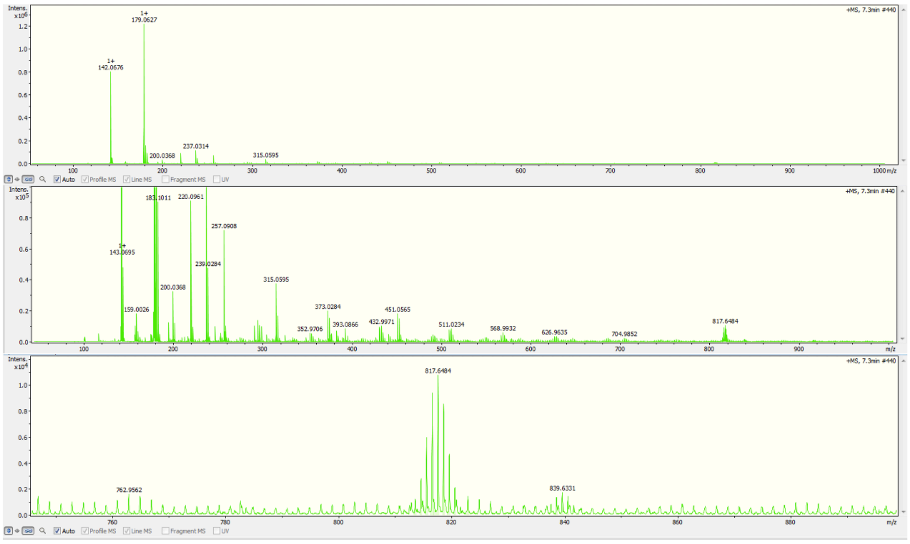

24 h

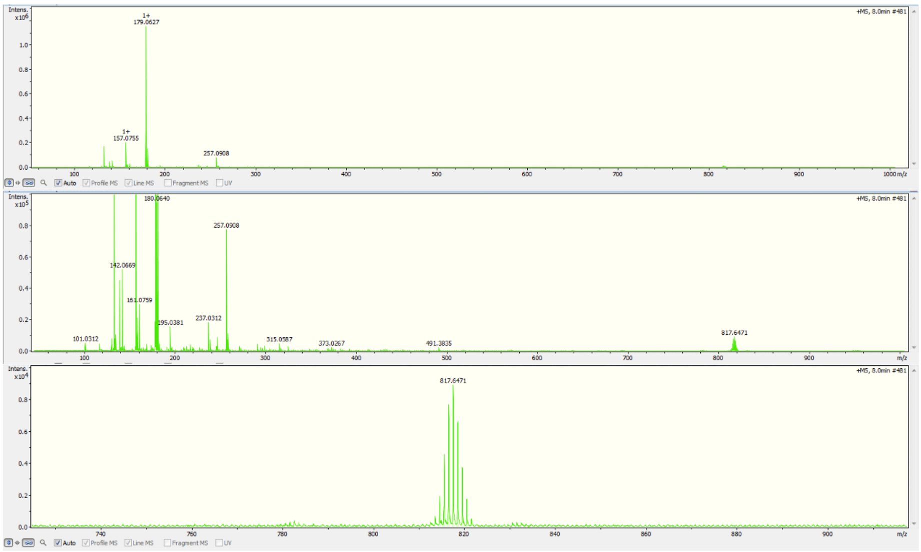

48 h

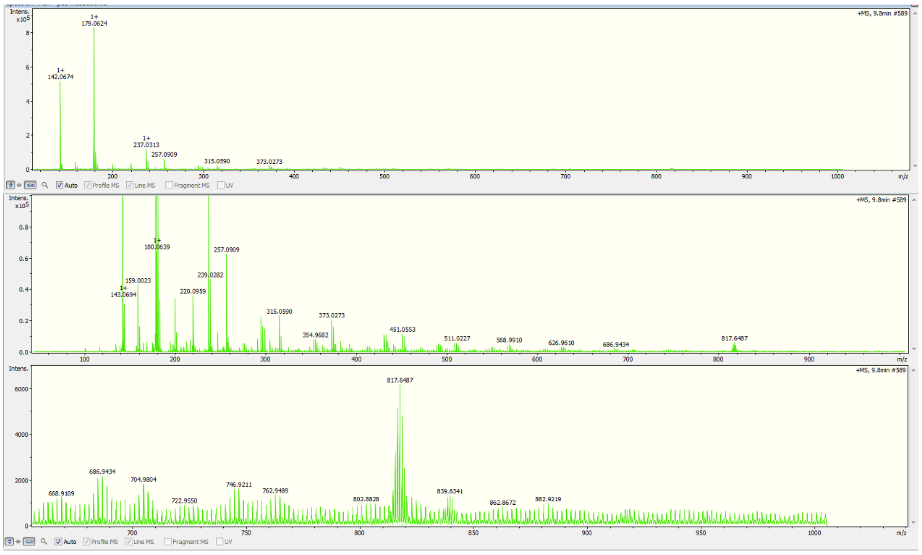

72 h

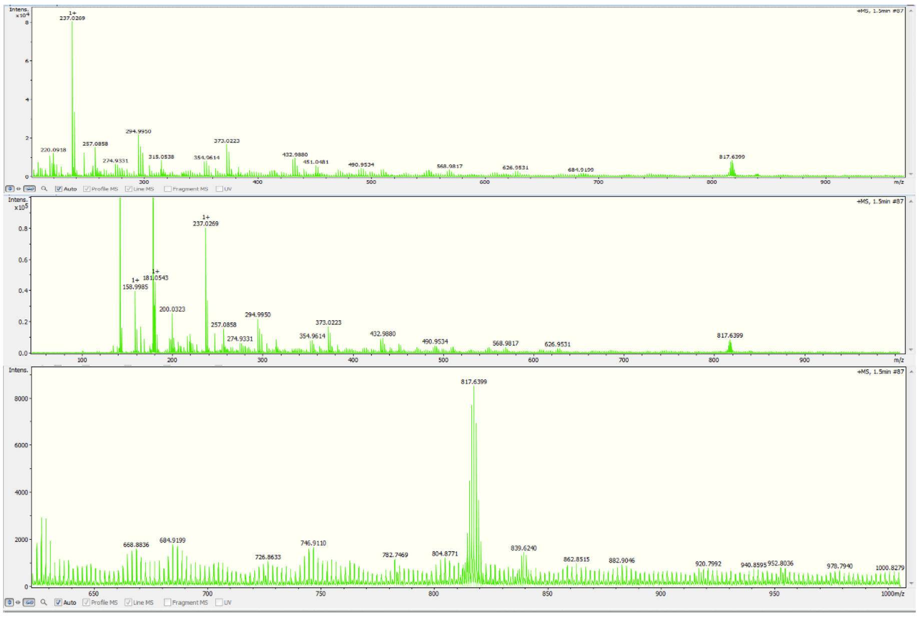

(B) HY8 (50 μM) in PBS

0 h

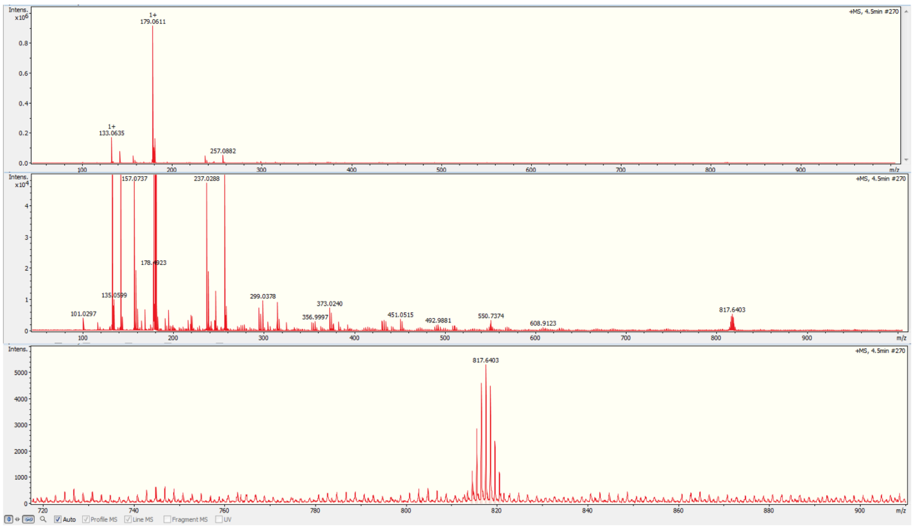

12 h

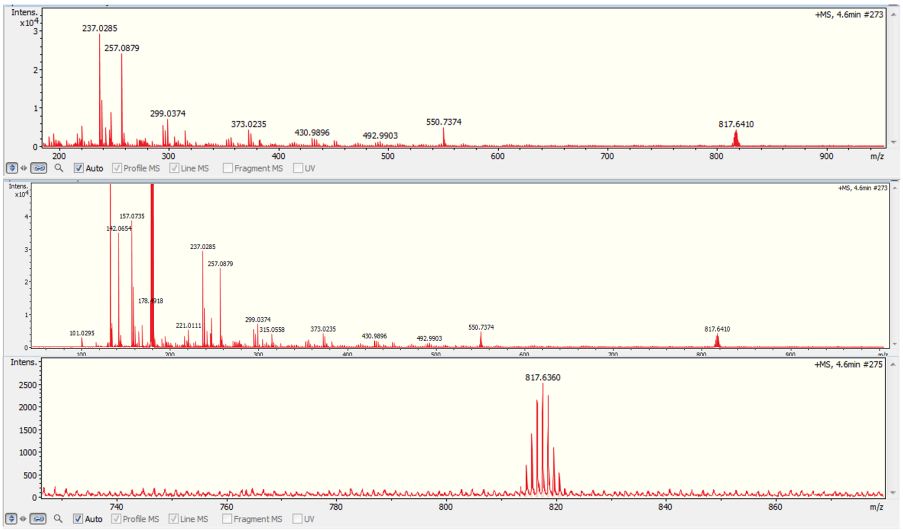

Intens.
x10^4
+MS, 4.6min #273
237.0285
257.0879
299.0374
373.0235
430.9896
492.9903
550.7374
817.6410
m/z
+MS, 4.6min #275
817.6360

24 h

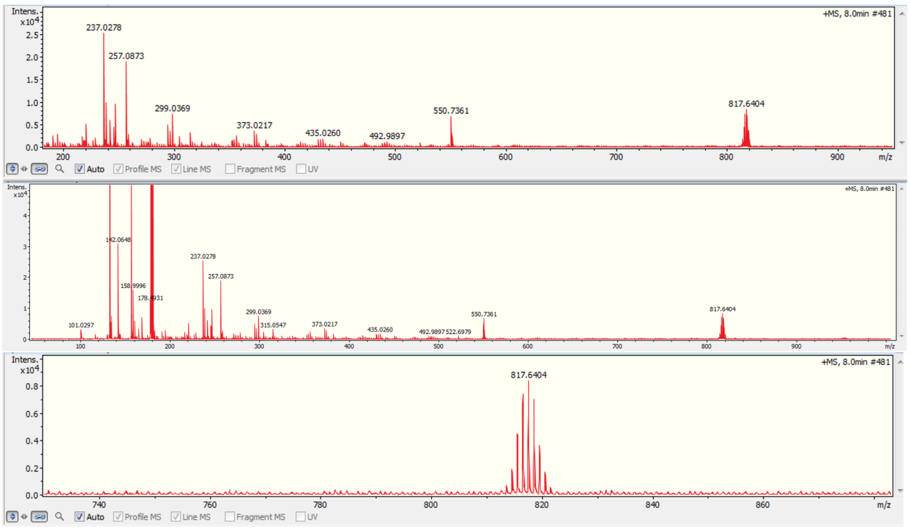

Intens.
x10^4
+MS, 8.0min #481
237.0278
257.0873
299.0369
373.0217
435.0260
492.9897
550.7361
817.6404
m/z

48 h

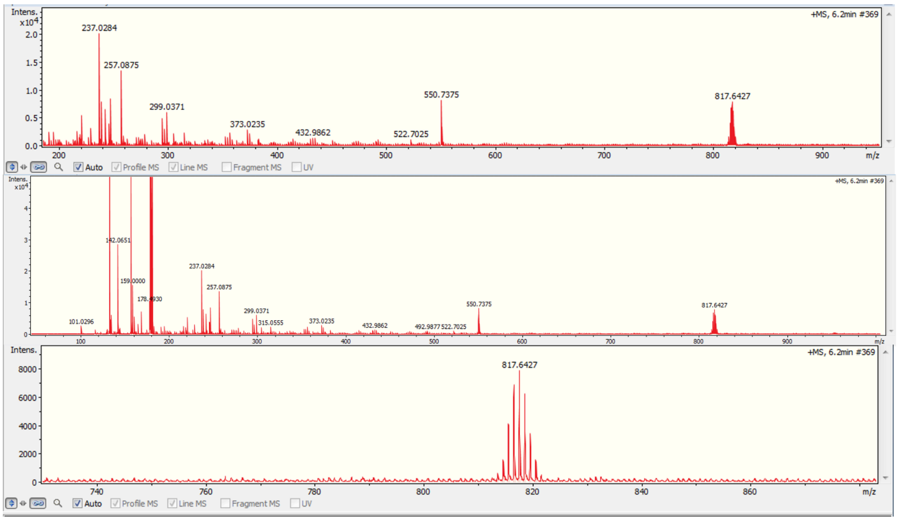

Intens.
x10^4
+MS, 6.2min #369
237.0284
257.0875
299.0371
373.0235
432.9862
522.7025
550.7375
817.6427
m/z

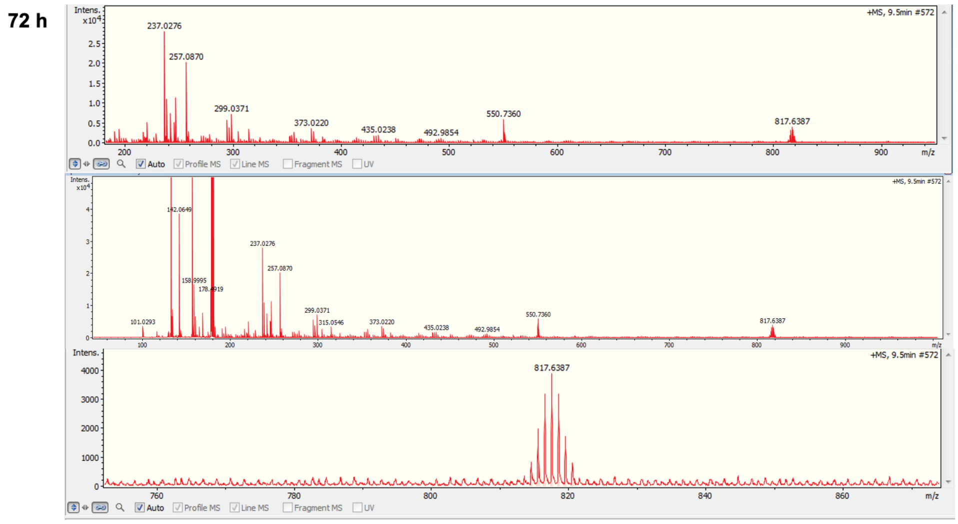

Figure S8. Representative ESI-TOF-MS of **HY8** (50 μM) incubated at 37℃ for different times in (A) PBS containing 10% FBS and (B) PBS.

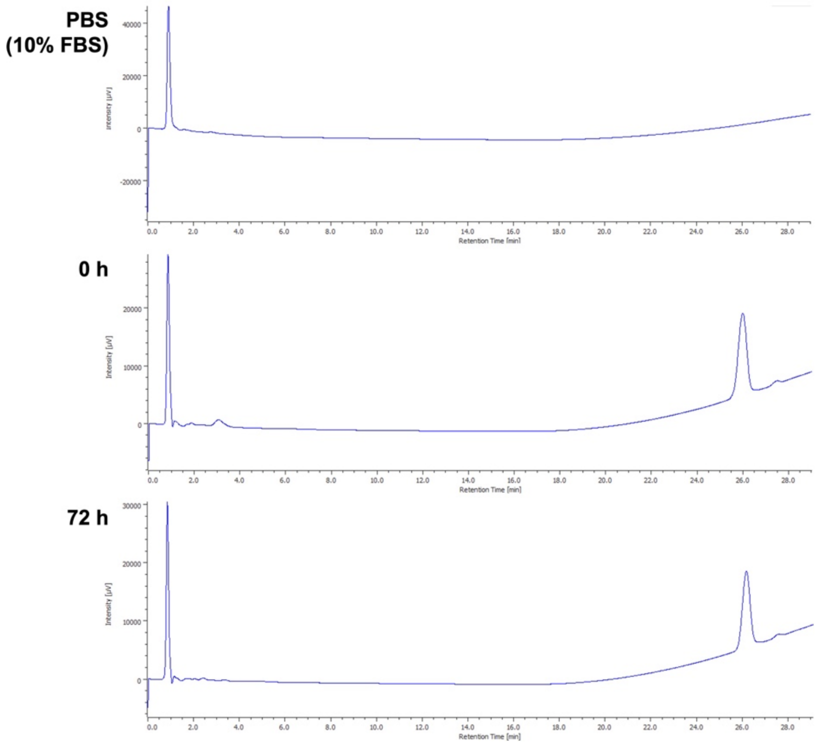

Figure S9. Representative HPLC chromatograms of **HY8** (50 μM) incubated in PBS containing 10% FBS at 37℃ for different time. HPLC analysis was performed under the same conditions as those used for the purity determination of the final compounds (**HY**s).

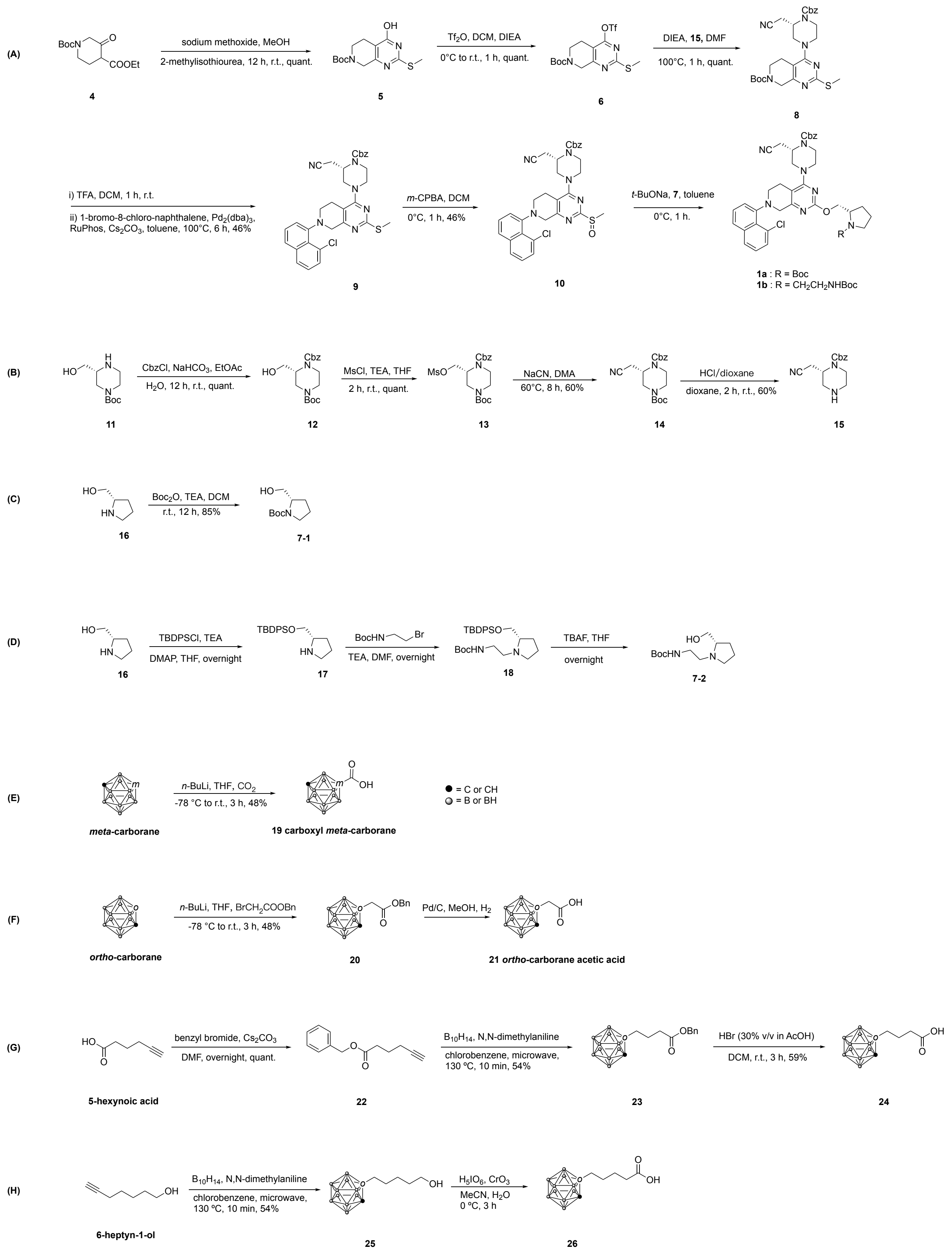

Scheme S1. Synthetic schemes of the intermediates.

2. Synthetic protocols

Synthesis of compound 5

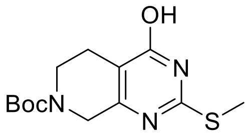

The synthetic route was followed the reported lectures[1,2]. To a solution of 1-*tert*-butyl 4-ethyl 3-oxopiperidine-1,4-dicarboxylate (compound **4**, 1.4 g, 5.0 mmol. 1.0 equiv.) in methanol (MeOH; 15.0 mL), sodium methoxide (1.35 g, 25.0 mmol, 5.0 equiv.) and 2-methylisothiourea (1.7 g, 9.0 mmol, 1.8 equiv.) were added at an argon atmosphere. After being stirred at room temperature for 12 h, the reaction mixture was concentrated under *vacuo*. Then obtained residue was dissolved in H_2O and acidified with 1 M HCl until pH~5. The mixture was extracted with ethyl acetate (EtOAc), wash with brine, dried over with magnesium sulfate ($MgSO_4$)and concentrated to give the desired compound **5** (1.5 g, 5.0 mmol, quant.). ^{1}H NMR (400 MHz, $CDCl_3$) δ 4.33 (2H, s), 3.60 (2H, m), 2.57 (5H, m), 1.50 (9H, s). HRMS (ESI, positive) for $C_{13}H_{19}O_3N_3S$ (*m/z*): calculated 320.1039 $(M+Na)^+$, found 320.1038.

Synthesis of compound 6

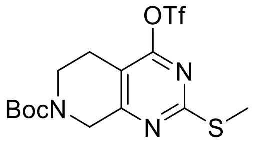

The synthetic route was followed the reported lectures[1,2]. To a solution of compound **5** (100.0 mg, 0.34 mmol, 1.0 equiv.) in dehydrated dichloromethane (DCM; 2.0 mL), *N,N*-diisopropylethylamine (DIEA; 118.0 µL, 0.68 mmol, 2.0 equiv.) and trifluoromethanesulfonic anhydride (Tf_2O; 85.0 µL, 0.51 mmol, 1.5 equiv.) was added at 0°C at an argon atmosphere. After being stirred at room temperature for 1 h, the reaction mixture was quenched by H_2O, extracted with EtOAc, dried over with $MgSO_4$ and concentrated under *vacuo*. The obtained residue was purified by column chromatography on silica gel (EtOAc: hexane = 80: 20) to give desired compound **6** (261.0 mg, 0.6 mmol, quant.) as a white solid. ^{1}H NMR (400 MHz, $CDCl_3$) δ 4.60 (s, 2H), 3.72-3.69 (t, 2H, J = 6.0 Hz), 2.76-2.73 (t, 2H, J = 5.6 Hz)), 2.53 (s, 3H), 1.48 (s, 9H). HRMS (ESI, positive) for $C_{14}H_{18}O_5N_3S_2F_3$ (*m/z*): calculated 452.0532 $(M+Na)^+$, found 450.0535.

Synthesis of compound 8

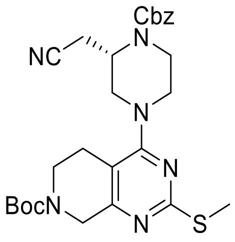

The synthetic route was followed the reported lectures[1,2]. To a solution of compound **6** (94.0 mg, 0.22

mmol, 1.0 equiv.) and benzyl (S)-2-(cyanomethyl)piperazine-1-carboxylate **15** (60.0 mg, 0.23 mmol, 1.05 equiv.) in *N,N*-dimethylformamide (DMF; 2.0 mL), DIEA (114.0 μL, 0.66 mmol, 3.0 equiv.) was added. After being stirring at 100 °C under an argon atmosphere, the reaction mixture was extracted with EtOAc, washed with brine, dried over with $MgSO_4$ and concentrated under *vacuo*. The obtained residue was purified by column chromatography on silica gel (EtOAc : hexane = 30 : 70) to give compound **8** (118 mg, yield quant.). ^{1}H NMR (400 MHz, $CDCl_3$) δ 7.38-7.33 (5H, m), 5.19 (2H, s), 4.66-4.59 (2H, m), 4.39-4.34 (1H, m), 4.11 (1H, s), 4.01-3.95 (1H, m), 3.92-3.77 (2H, m), 3.31-3.28 (3H, m), 3.03-2.99 (1H, m), 2.78-2.57 (4H, m), 2.50 (3H, s), 1.49 (9H, s).

Synthesis of compound 9

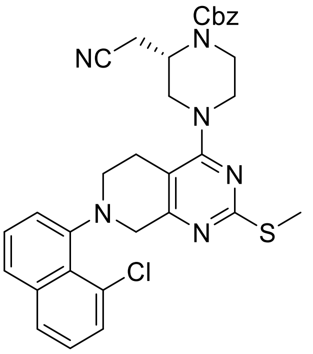

The synthetic route was followed the reported lectures[1,2]. To a solution of compound **8** (100.0 mg, 0.18 mmol, 1.0 equiv.) in DCM (2.0 mL), trifluoroacetic acid (TFA; 500.0 μL) was added. After being stirring at room temperature for 1 h, the reaction mixture was extracted with DCM, washed with $NaHCO_3$ aq., dried over with $MgSO_4$, and concentrated under *vacuo*. The obtained residue was used for next step without further purification.

To a solution of the intermediate (40.0 mg, 0.09 mmol, 1.0 equiv.), 1-bromo-8-chloro-naphthalene (52.0 mg, 0.22 mmol, 1.8 equiv.), Tris(dibenzylideneacetone)dipalladium(0) ($Pd_2(dba)_3$; 22.0 mg, 0.024 mmol, 0.2 equiv.), 2-dicyclohexylphosphino-2',6'-diisopropoxybiphenyl (RuPhos; 17.0 mg, 0.036 mmol, 0.3 equiv.) and cesium carbonate(Cs_2CO_3, 140.0 mg, 0.432 mmol, 3.6 equiv.) in toluene (2.0 mL) was stirred at 100°C under argon atmosphere for 6 h. Then, the reaction mixture was filtered. The organic solvent was removed under vacuum to give an oil residue. The obtained residue was purified by column chromatography on silica gel (EtOAc : hexane = 60 : 40) to give the desired compound **9** (25.0 mg, 0.042 mmol, yield 46% for two steps). ^{1}H NMR (400 MHz, $CDCl_3$) δ 7.74 (1H, d, J = 8.2 Hz), 7.62-7.59 (1H, t, J = 7.4 Hz), 7.53-7.50 (1H, m), 7.47-7.41 (1H, m), 7.39-7.31 (5H, m), 7.34-7.31 (1H, m), 7.19 (1H, d, J = 7.5 Hz), 5.20 (2H, s), 4.71-4.68 (1H, brs), 4.47-4,40 (1H, m), 4.15-4.04 (1H, m), 3.96-3.89 (1H, m), 3.84-3.75 (1H, m), 3.58-3.48 (1H, m), 3.40 (1H, d, J = 10.7 Hz), 3.29-3.08 (4H, m), 3.01-2.92 (1H, m), 2.85-2.66 (2H, m), 2.60-2.57 (1H, m), 2.50 (3H, d, J = 3.3 Hz). HRMS (ESI, positive) for $C_{32}H_{31}O_2N_6SCl$ (*m/z*): calculated 599.1990 $(M+H)^+$, found 599.1994.

Synthesis of compound 10

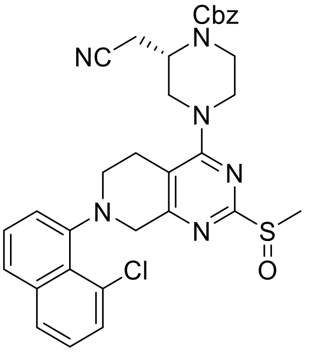

The synthetic route was followed the reported lectures[1,2]. To a solution of compound **9** (360.0 mg, 0.51 mmol, 1.0 equiv.) in DCM (8.0 mL), *m*-chloroperoxybenzoic acid *(m*-CPBA; 197.0 mg, 0.72 mmol, 1.2 equiv.) was added under 0°C. After being stirring for 1 h, the reaction mixture was extracted with DCM, washed with $NaHCO_3$ aq., dried over with $MgSO_4$ and concentrated under *vacuo*. The obtained residue was purified by column chromatography on silica gel (EtOAc : hexane = 80 : 20) to give the desired compound **10** (170.0 mg, 0.27 mmol, yield 46%). The reported literature states, "The products were obtained as a mixture that was not separable by column chromatography" [1,2]. We confirmed the purification of the compound **10** by HRMS in accordance with the previous reports. HRMS (ESI, positive) for $C_{32}H_{31}O_3N_6ClS$ (*m/z*): calculated 616. 1925 $(M+H)^+$, found 616.1921.

Synthesis of compound 1a

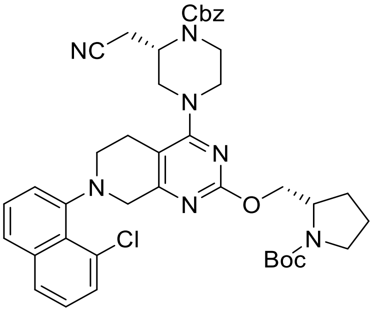

To a solution of compound **10** (170.0 mg, 0.28 mmol, 1.0 equiv.) and compound **7-1** (169.0 mg, 0.84 mmol, 3.0 equiv.) in toluene (4.0 mL), sodium *tert*-butoxide (*t*-BuONa; 81.0 mg, 0.84 mmol, 3.0 equiv.) was added under 0°C at argon atmosphere. After being stirring at 0℃ for 1 h, the reaction mixture was extracted with EtOAc, washed with water and brine, dried over with $MgSO_4$, and concentrated under *vacuo*. The obtained residue was purified by column chromatography on silica gel (EtOAc : hexane = 80 : 20) to give the desired compound **1a** (100.0 mg, 0.13 mmol, yield 48%). The products were obtained as a mixture that was not separable by column chromatography. Therefore, we confirmed the purification of the compound by HRMS. HRMS (ESI, positive) for $C_{41}H_{46}O_5N_7Cl$ (*m/z*): calculated 752.3322 $(M+H)^+$, found 752.3318.

Synthesis of 1b

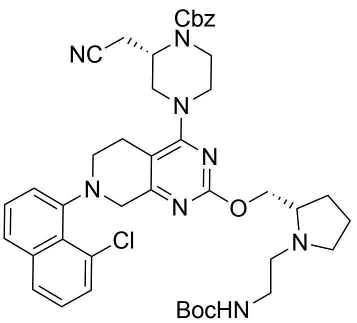

To a solution of compound **10** (180.0 mg, 0.29 mmol) in toluene (4.0 mL), compound **7-2** (214.0 mg, 0.87 mmol) and sodium *tert*-butoxide(80.0 mg, 0.87 mmol, 3.0 equiv.) were added under argon atmosphere at 0 °C. After being stirred for 1 h at 0℃, the reaction was quenched by NH_4Cl aq. The reaction mixture was extracted with EtOAc for three times. The organic layer was washed with brine, dried over with $MgSO_4$, and concentrated under *vacuo*. The obtained residue was purified by column chromatography on silica (MeOH : DCM = 10 : 90) to give the desired compound **1b** (71.0 mg, 0.09 mmol, yield 33%). The products were obtained as a mixture that was not separable by column chromatography. Therefore, we confirmed the purification of the compound by HRMS. HRMS (ESI, positive) for $C_{43}H_{51}O_5N_8Cl$ (*m/z*): calculated 795.3744 $(M+H)^+$, found 795.3740.

Synthesis of compound 14

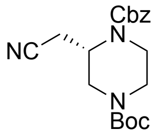

The synthetic route was followed the reported lectures[1,2]. To a stirred solution of (R)-*tert*-butyl 3-(hydroxymethyl)piperazine-1-carboxylate **11** (commercially available, 1.0 g, 4.6 mmol, 1.0 equiv.) and sodium bicarbonate ($NaHCO_3$; 1.2 g, 13.9 mmol, 3.0 equiv.) in EtOAc (10.0 mL) and H_2O (5.0 mL), benzyl chloroformate (850.0 μL, 6.0 mmol, 1.3 equiv.) was added dropwise under an argon atmosphere. After being stirred at room temperature for 12 h, the reaction mixture was extracted with EtOAc, washed with brine. The combined organic layer was dried over with $MgSO_4$ and concentrated under *vacuo* to give the desired intermediate **12** (1.6 g, 4.6 mmol, quant.) without purification for next step. ^1H NMR (400 MHz, $CDCl_3$) δ 7.38-7.32 (5H, m), 5.18-5.11 (2H, m), 4.26 (1H, brs), 3.94 (2H, brs), 3.63 (2H, brs), 3.04-2.83 (4H, m), 1.47 (9H, s). HRMS (ESI, positive) for $C_{18}H_{26}O_5N_2$ (*m/z*): calculated 373.1734 $(M+Na)^+$, found 373.1731.

To a solution of intermediate **12** (130.0 mg, 0.37 mmol, 1.0 equiv.) in dehydrated tetrahydrofuran (THF; 2.5 mL), TEA (158.0 μL, 1.14 mmol, 3.0 equiv.) and methanesulfonyl chloride (MsCl; 34.0 μL, 0.44 mmol, 1.2 equiv.) was added under an argon atmosphere. After being stirred at room temperature for 2 h, the reaction mixture was quenched by H_2O, extracted with EtOAc, washed with brine. The combined organic layer was dried over with $MgSO_4$ and concentrated under *vacuo* to give the crude product intermediate **13** (202.0 mg, 0.47 mmol, quant.) without purification for next step.

To a solution of crude product **13** (250.0 mg, 0.58 mmol, 1.0 equiv.) in dehydrated dimethylacetamide (DMA; 2.0 mL), sodium cyanide (NaCN; 114.0 mg, 2.33 mmol, 4.0 equiv.) was added under an argon

atmosphere at 0°C. After being stirred at 60°C for 8 h, the reaction mixture was diluted with EtOAc and water. The residue was extracted with EtOAc and washed with brine. The combined organic layer was dried over with $MgSO_4$ and concentrated under *vacuo.* The residue was purified by column chromatography on silica gel (EtOAc : hexane = 50 : 50) to give desired compound **14** (125.0 mg, 0.35 mmol, yield 60% for 3 steps). ^{1}H NMR (400 MHz, $CDCl_3$) δ 7.37-7.35 (5H, m), 4.55 (2H, s), 4.03 (1H, brs), 4.03 (2H, brs), 3.20-3.04 (2H, m), 3.01-2.94 (2H, m), 2.88-2.48 (2H, m), 1.48 (9H, s). HRMS (ESI, positive) for $C_{19}H_{25}O_4N_3$ (*m/z*): calculated 382.1737 $(M+Na)^+$, found 382.1741.

Synthesis of compound 15

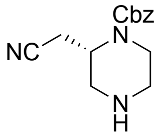

The synthetic route was followed the reported literature[1,2]. To a solution of compound **14** (125.0 mg, 0.35 mmol, 1.0 equiv.) in dehydrated dioxane (3.0 mL), HCl solution (dissolved in dioxane, 4 M) was added under an argon atmosphere. After being stirred at room temperature for 2 h, the reaction was quenched with saturated $NaHCO_3$ solution and extracted with EtOAc. The combined organic layer was dried over with $MgSO_4$ and concentrated under *vacuo*. The obtained residue was purified by column chromatography on silica gel (EtOAc) to give desired compound **15** (61.0 mg, 0.23 mmol, yield 68%). ^{1}H NMR (400 MHz, $CDCl_3$) δ 7.36-7.33 (m, 5H), 5.16 (s, 2H), 4.51 (brs, 1H), 3.97 (brs, 1H), 3.04-2.87 (m, 5H), 2.77-2.66 (m, 2H). HRMS (ESI, positive) for $C_{14}H_{17}O_2N_3$ (*m/z*): calculated 282.1213 $(M+Na)^+$, found 282.1214.

Synthesis of compound 7-1

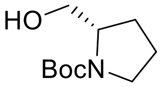

To a stirred solution of L-prolinol (200.0 mg, 1.98 mmol, 1.0 equiv.) in DCM (5.0 mL), TEA (1.38 mL, 9.9 mmol, 5.0 equiv.) and di-*t*-butyl dicarboante (Boc_2O; 518.0 mg, 2.4 mmol, 1.2 equiv.) were added at room temperature. After being stirred at room temperature for 24 h under open to air condition, the reaction mixture was extracted with DCM and brine, dried over with $MgSO_4$ and concentrated under *vacuo* to give the desired compound **7-1** (339.0 mg, 1.68 mmol, yield 85%) without purification for next step. ^{1}H NMR (400 MHz, $CDCl_3$) δ 4.69 (brs, 1H), 3.97 (brs, 1H), 3.65-3.56 (m, 2H), 3.49-3.43 (m, 1H), 3.35-3.29 (m, 1H), 2.04-1.98 (m, 1H), 1.85-1.78 (m, 2H), 1.49 (s, 9H).

Synthesis of compound 17

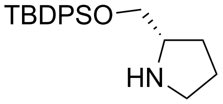

The synthetic route was followed the reported literatures[1,2]. To a stirred solution of L-prolinol (1.0 g, 9.9 mmol, 1.0 equiv.) in THF (20.0 mL), TEA (4.1 mL, 29.7 mmol, 3.0 equiv.), DMAP (120.0 mg, 1.0 mmol,

0.1 equiv.) and *tert*-butyldiphenylchlorosilane (TBDPSCl; 3.0 mL, 11.8 mmol, 1.2 equiv.) were added at room temperature. After being stirred at room temperature for overnight, the reaction mixture was extracted with DCM and brine, dried over with $MgSO_4$ and concentrated under *vacuo* to obtain the resulting mixture. The obtained residue was purified by column chromatography on silica gel (MeOH : DCM = 20 : 80) to give desired compound **17** as yellow oil (3.1 g, 98%). ^{1}H NMR (400 MHz, $CDCl_3$) δ 7.68-7.65 (4H, m), 7.42-7.36 (6H, m), 3.65-3.56 (2H, m), 3.23-3.20 (1H, m), 2.99-2.94 (1H, m), 2.87-2.80 (1H, m), 1.75-1.70 (3H, m), 1.49-1.45 (1H, m), 1.05 (9H, s).

Synthesis of compound 18

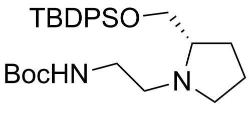

The synthetic route was followed the reported literatures[3].To a stirred solution of compound **17** (3.1 g, 9.1 mmol, 1.0 equiv.) in DMF (10.0 mL), TEA (3.8 mL, 27.3 mmol, 3.0 equiv.) and 2-(tert-butoxycarbonylamino)ethyl bromide (2.0 g, 9.1 mmol, 1.0 equiv.) were added. After being stirred at room temperature for overnight, the reaction mixture was extracted with EtOAc, washed with brine, dried over with $MgSO_4$, and concentrated under *vacuo*. The obtained residue was purified by column chromatography on silica gel (EtOAc: hexane = 40: 60) to give the desired compound **18** (1.89 g, yield 43%). ^{1}H NMR (400 MHz, $CDCl_3$) δ 7.68-7.66 (4H, m), 7.40-7.38 (6H, m), 4.99 (1H, brs), 3.63-3.46 (2H, m), 3.22-3.04 (2H, m), 2.93-2.88 (1H, s), 2.64-2.60 (1H, m), 2.42 (1H, brs), 2.23-2.17 (1H, m), 1.88-1.83 (1H, m), 1.71-1.67 (2H, m), 1.60-1.58 (2H, m), 1.41 (9H, s), 1.05 (9H, s).

Synthesis of compound 7-2

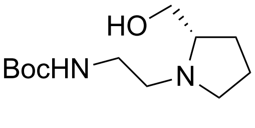

To a stirred solution of compound **18** (1.8 g, 3.7 mmol, 1.0 equiv.) in THF (10.0 mL), tetrabutylammonium fluoride (TBAF; 7.4 mL, 7.4 mmol, 2.0 equiv.) was added slowly under room temperature. After being stirred at room temperature for overnight, the reaction mixture was extracted with DCM, washed with brine, dried over with $MgSO_4$, and concentrated under *vacuo*. The obtained residue was purified by column chromatography on silica gel (MeOH: DCM = 10: 90) to give desired compound **7-2** (1.4 g, yield quant.) as yellow oil. ^{1}H NMR (400 MHz, $CDCl_3$) δ 5.14 (1H, brs), 3.70-3.67 (1H, m), 3.54-3.51 (1H, m), 3.37-3.26 (2H, m), 3.03-2.88 (2H, m), 2.64- 2.48 (2H, m), 1.93 (1H, brs), 1.82 (2H, brs), 1.42 (9H, s).

Synthesis of compound 19

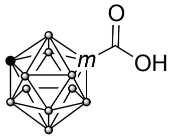

The synthetic route was followed the reported literature[4]. To a stirred solution of *meta*-carborane (288.0 mg, 2.0 mmol, 1.0 equiv.) in THF (4.0 mL) at -78°C under argon atmosphere, *n*-BuLi (1.6M in hexane, 1.4 mL, 2.2 mmol, 1.1 equiv.) was added dropwise. After being stirred at room temperature for 1 h, the balloon of carbon dioxide was attached and degassed for three times. Then, the resulting solution was stirred under room temperature for further 2 h. The residue was extracted with EtOAc and brine, dried over with $MgSO_4$, and concentrated under *vacuo* to obtain desired compound **19** (173.0 mg, 48%) as white solid without further purification.

Synthesis of compound 20

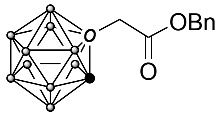

The synthetic route was followed the reported literature[4]. To a stirred solution of *ortho*-carborane (72.0 mg, 0.5 mmol, 1.0 equiv.) in THF (4.0 mL) at 0°C under argon atmosphere, *n*-BuLi (1.6M in hexane, 0.35 mL, 0.53 mmol, 1.05 equiv.) was added dropwise. After being stirred for 1 h at room temperature, benzyl bromoacetate (0.085 mL, 0.55 mmol, 1.1 equiv.) was added dropwise. Then, the resulting solution was stirred under room temperature for further 2 h. The residue was extracted with EtOAc and brine, dried over with $MgSO_4$ and concentrated. The residue was purified by column chromatography on silica gel (EtOAc : hexane = 20 : 80) to give desired compound **20** (120.0 mg, yield 82%) as yellow oil. ^{1}H NMR (400 MHz, $CDCl_3$) δ 7.40-7.34 (5H, m), 5.14 (2H, s), 3.56 (1H, s), 3.28 (2H, s), 2.80-1.45 (10H, m).

Synthesis of compound 21

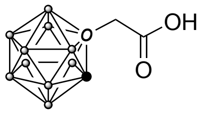

The synthetic route was followed the reported literature[4]. To a stirred solution of compound **20** (120.0 mg, 0.4 mmol, 1.0 equiv.) in MeOH (4.0 mL) under argon atmosphere, Pd/C (60.0 mg, 50%wt/wt) was added. Then, the hydrogen balloon was attached and degassed for three times. The resulting mixture was stirred at room temperature for overnight. After filtration, the residue was concentrated to give the desired compound **21** (70.0 mg, yield 84%) as yellow solid used for next step without further purification.

Synthesis of compound 22

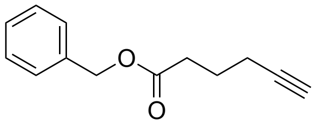

The synthetic route was followed the reported literature[6]. To a stirred solution of 5-hexynoic acid (1.0 g, 10 mmol, 1.0 equiv.) and cesium carbonate (812.0 mg, 2.5 mmol, 0.25 equiv.) in DMF (20.0 mmol), benzyl bromide (1.3 mL, 10.5 mmol, 1.05 equiv.) was added under argon atmosphere. After being stirred at room temperature for overnight, the reaction mixture was extracted with EtOAc, washed with brine, dried over with $MgSO_4$ and concentrated under *vacuo* to give the desired compound **22** (800.0 mg, yield 40%) used for next step without further purification. ^{1}H NMR (400 MHz, $CDCl_3$) δ 7.37-7.34 (5H, m), 5.12 (2H, s), 2.53-2.49 (2H, t, J = 7.6 Hz), 2.29-2.25 (2H, m), 1.96-1.95 (1H, t, J = 2.64 Hz), 1.91-1.84 (2H, m).

Synthesis of compound 23

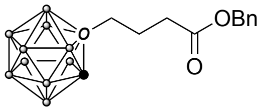

To a solution of compound **22** (800.0 mg, 4.0 mmol, 1.0 equiv.) and *N,N*-dimethylaniline (0.76 mL, 6.0 mmol, 1.5 equiv.) in chlorobenzene (8.0 mL), decarborane (587.0 mg, 4.8 mmol 1.2 equiv.) was added. After being stirred at 130 °C under argon atmosphere for 10 min with microwave synthesizer, the reaction mixture was extracted with Et_2O, washed with H_2O. The combined organic layer was dried over $MgSO_4$ and concentrated under *vacuo*. The obtained residue was purified by column chromatography on silica gel (EtOAc: hexane = 10: 90) to give desired compound **23** (700.0 mg, 54%) as a white solid. ^{1}H NMR (400 MHz, $CDCl_3$) δ 7.34-7.33 (5H, m), 5.11 (2H, s), 3.49 (1H, s), 3.05-1.37 (10H, m), 2.37-2.33 (2H, t, J = 6.9 Hz), 2.23-2.19 (2H, m), 1.84-1.76 (2H, m).

Synthesis of compound 24

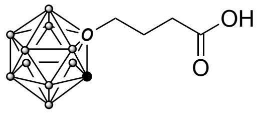

The synthetic route was followed the reported literature[4]. To a stirred solution of compound **23** (700.0 mg, 2.2 mmol, 1.0 equiv.) in DCM (6.0 mL), HBr (30% in AcOH, 4.0 mL) was added under argon atmosphere. After being stirred at room temperature for 3 h, the reaction mixture was quenched with brine and extracted with DCM, dried over $MgSO_4$, and concentrated under *vacuo*. The obtained residue was purified by column chromatography on silica gel (EtOAc : hexane = 40 : 60) to give desired compound **24** (300.0 mg, 59%). ^{1}H NMR (400 MHz, DMSO-d_6) δ 12.15 (1H, s), 5.19 (1H, s), 2.77-1.42 (10H, m), 2.31-2.21 (4H, m), 1.66-1.58 (2H, m).

Synthesis of compound 26

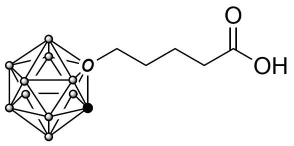

The synthetic protocol is followed the above condition. To a solution of 6-heptyn-1-ol (500.0 mg, 4.4 mmol, 1.0 equiv.) and *N,N*-dimethylaniline (0.84 mL, 6.7 mmol, 1.5 equiv.) in chlorobenzene (8.0 mL), decarborane

(652.0 mg, 5.3 mmol 1.2 equiv.) was added. After being stirred at 130 °C under argon atmosphere for 10 min with microwave synthesizer, the reaction mixture was extracted with Et_2O and washed with H_2O. The combined organic layer was dried over $MgSO_4$ and concentrated under *vacuo*. The obtained residue was purified by column chromatography on silica gel (EtOAc: hexane = 10: 90) to give desired compound **25** (800.0 mg, 77%) as a intermediate. To a stirred solution of compound **25** (800.0 mg, 3.4 mmol, 1.0 equiv.) in MeCN (11.0 mL), the mixture of cesium carbonate (1.2 mol% to orthoperiodic acid) and orthoperiodic acid (1.9 g, 8.3 mmol) in MeCN (20.0 mL) was added under argon atmosphere at 0°C. After being stirred at room temperature for 3 h, the reaction mixture was quenched with $NaHCO_3$ and extracted with EtOAc. The combined organic layer was dried over $MgSO_4$ and concentrated under *vacuo* to give the desired compound **26** (380.0 mg, yield 46%) as a white solid. ^{1}H NMR (400 MHz, DMSO-d_6) δ 12.05 (1H, s), 5.16 (1H, s), 2.28-2.42 (2H, t, J = 12 Hz), 2.22-2.18 (2H, t, J = 6.8 Hz), 1.44-1.43 (4H, m), 1.20-2.50 (10H, m).

3. ^{1}H, ^{13}C and HPLC Spectra

Compound 5

^{1}H NMR ($CDCl_3$, 400 MHz)

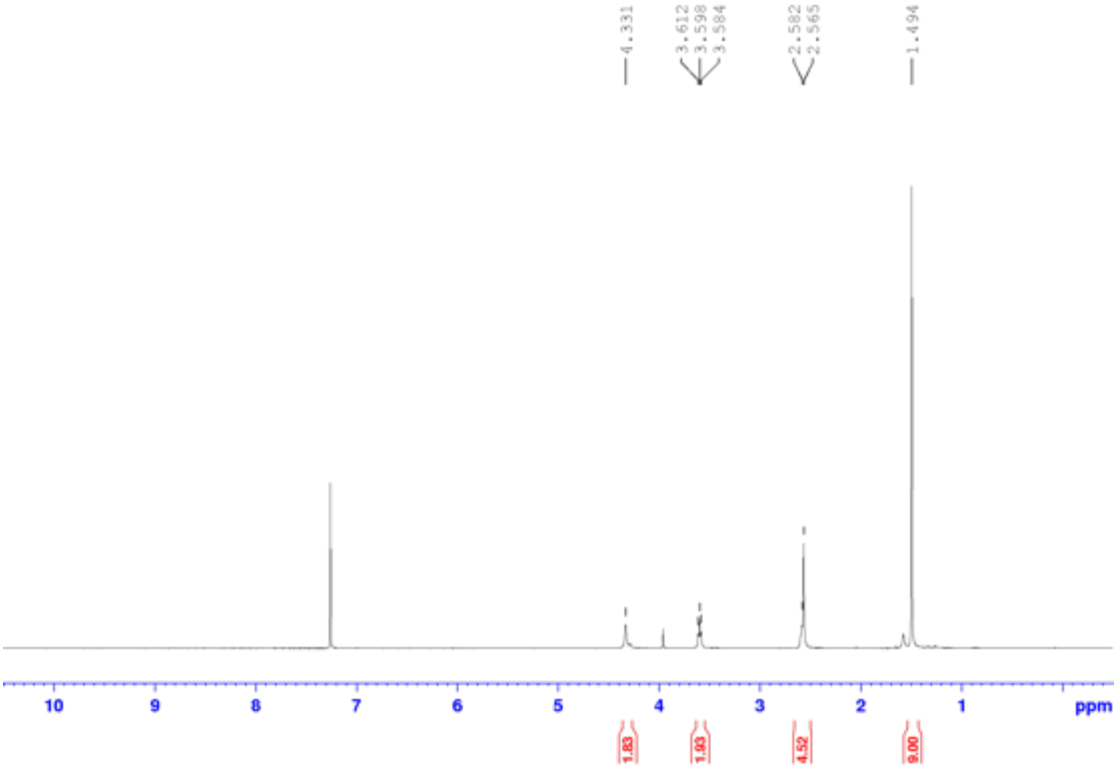

Compound 6

^{1}H NMR ($CDCl_3$, 400 MHz)

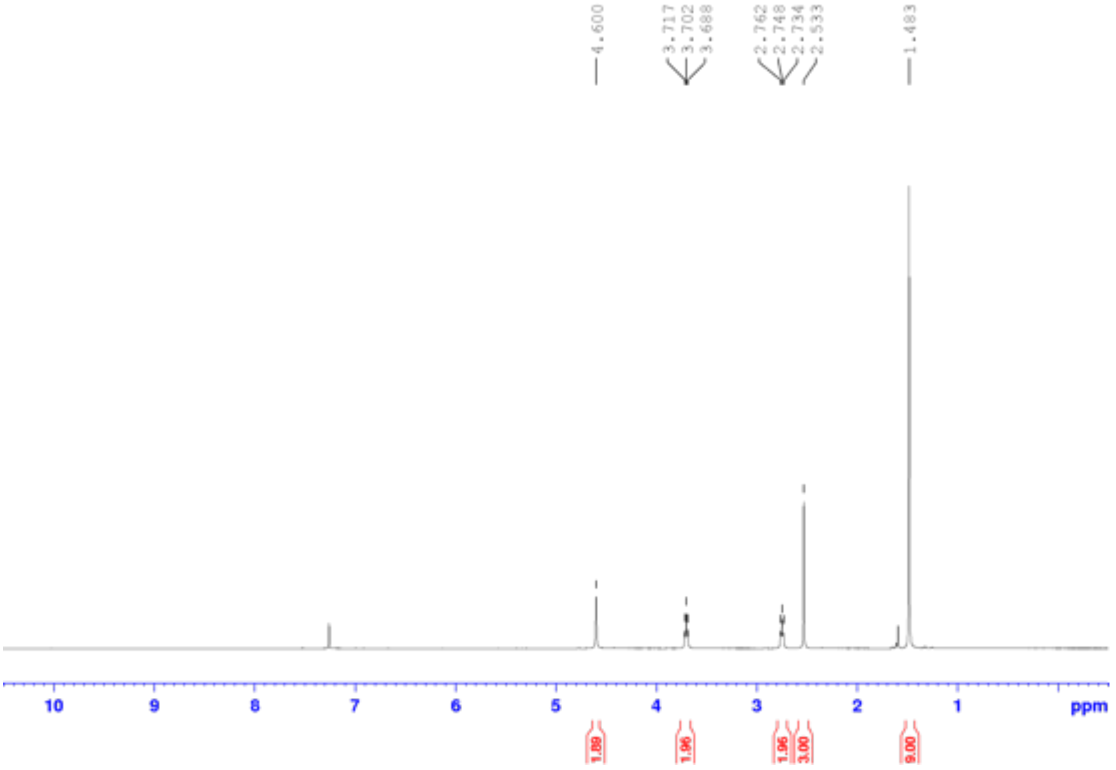

Compound 8 intermediate

^{1}H NMR ($CDCl_3$, 400 MHz)

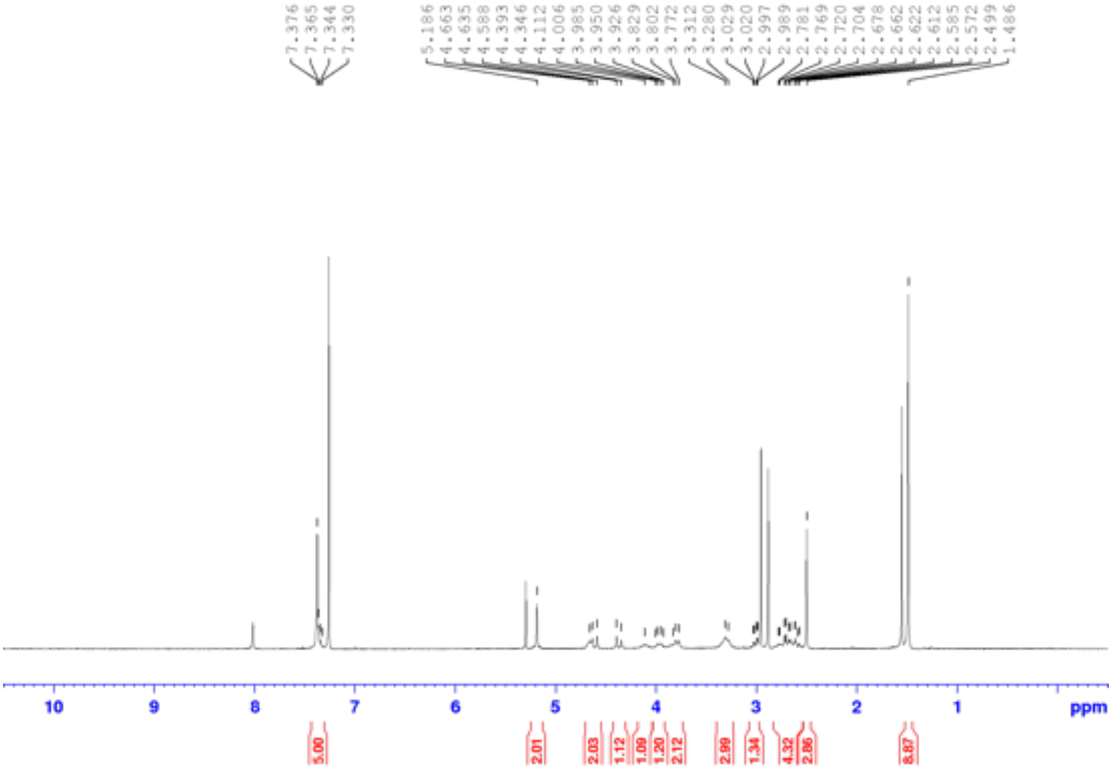

Compound 8

^{1}H NMR ($CDCl_3$, 400 MHz)

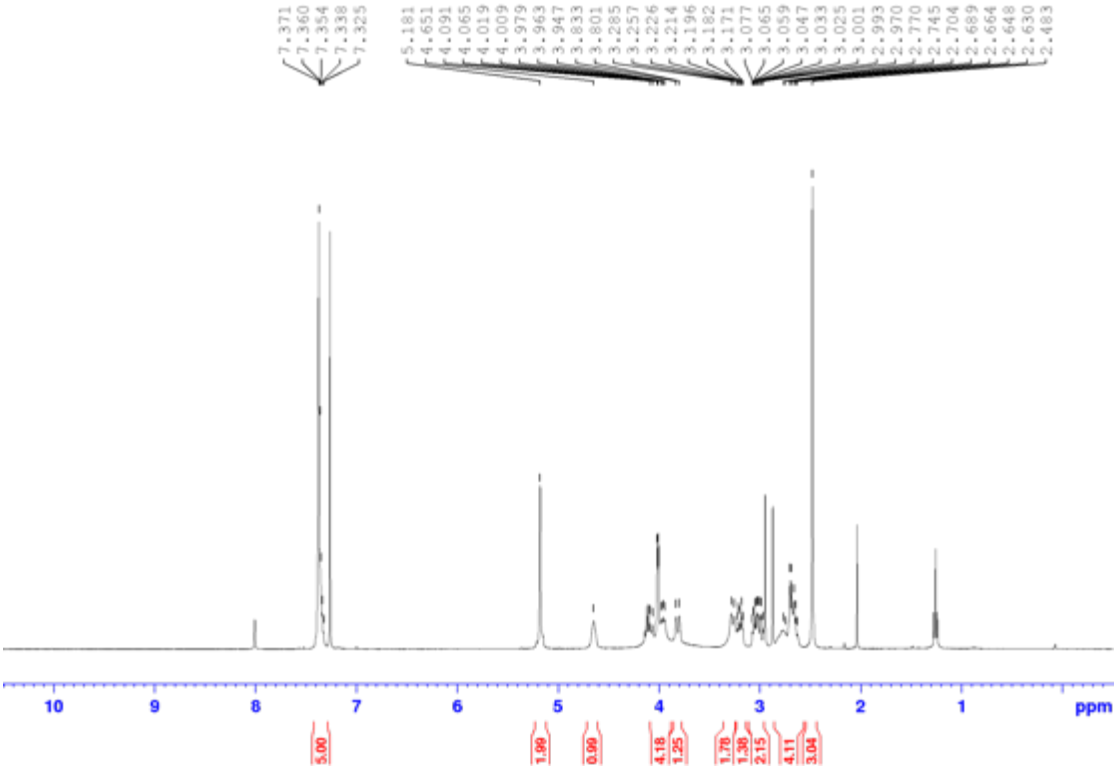

Compound 9

^1H NMR ($CDCl_3$, 400 MHz)

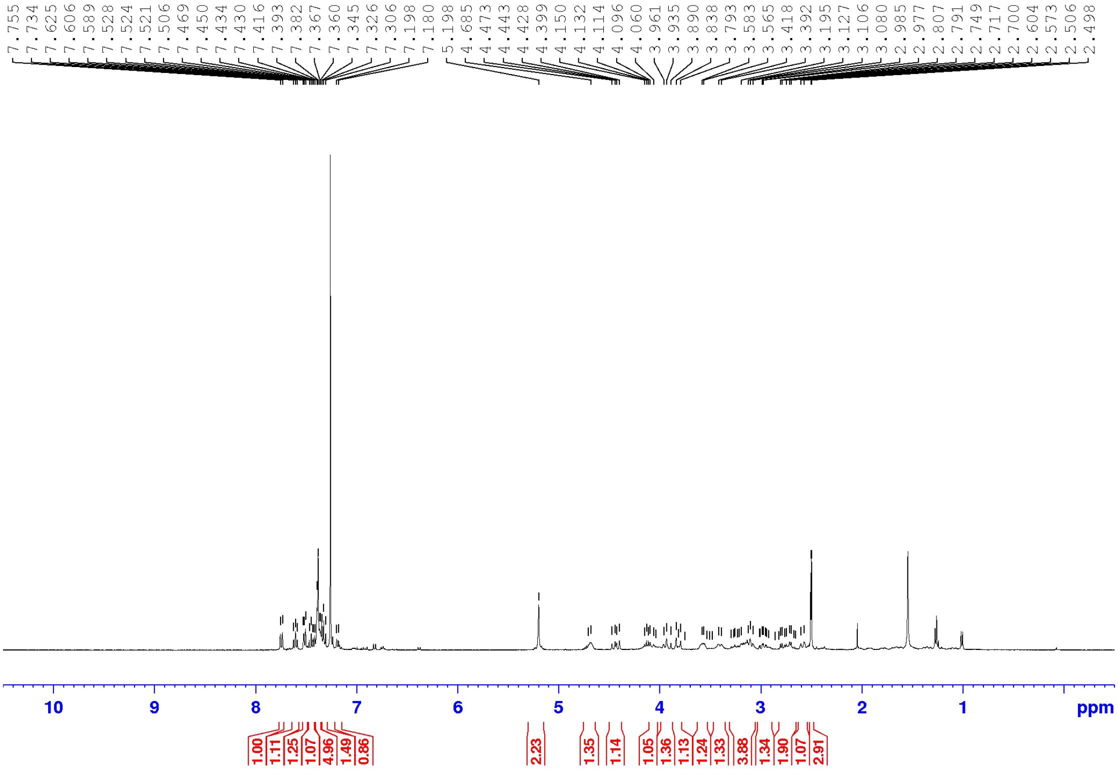

Compound HY1

^1H NMR ($CDCl_3$, 500 MHz)

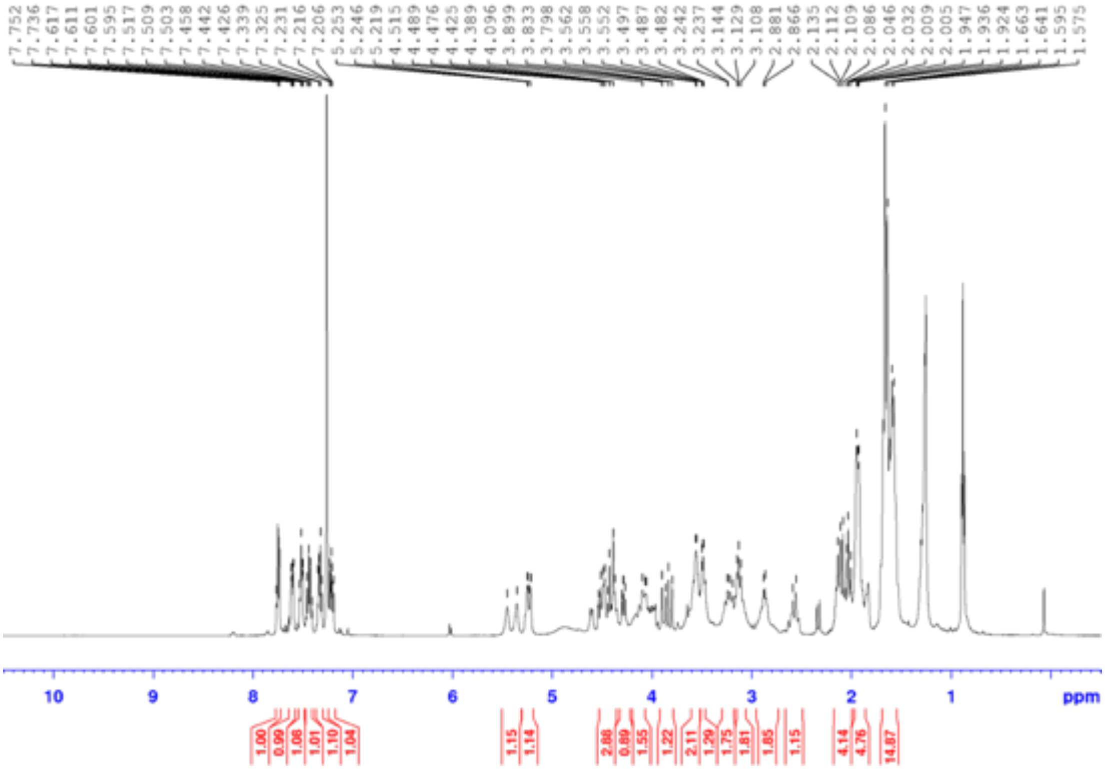

^{13}C NMR ($CDCl_3$, 125 MHz)

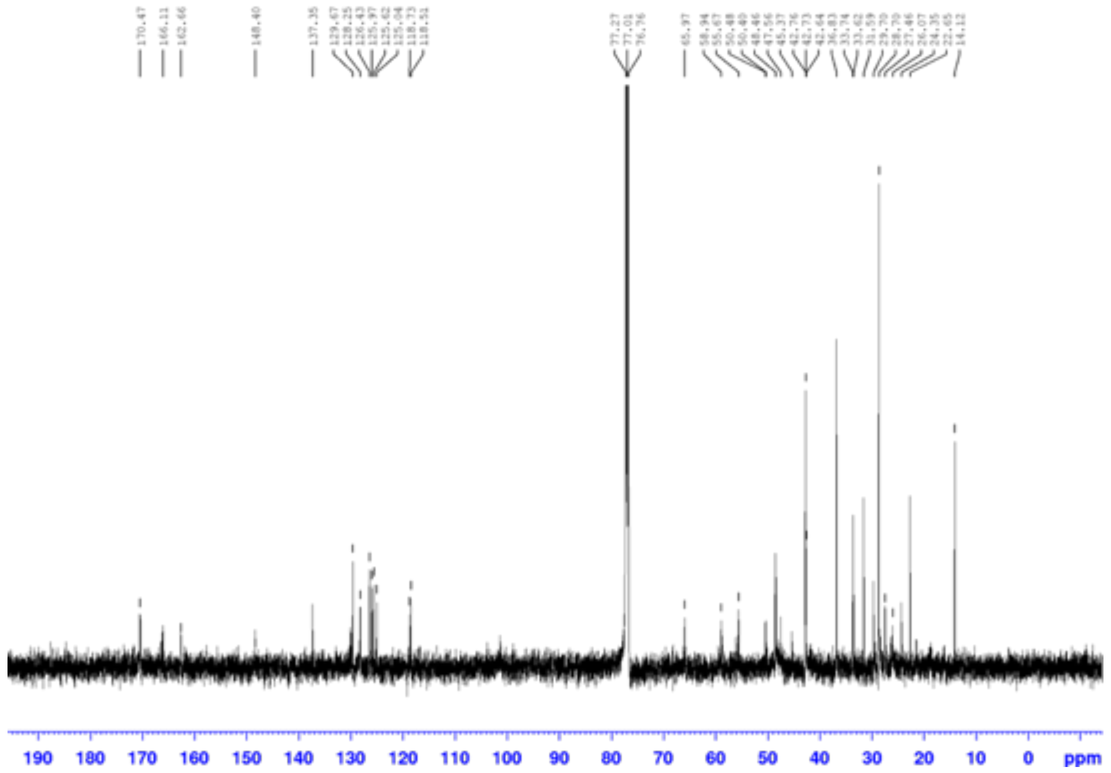

Compound HY2

^{1}H NMR ($CDCl_3$, 500 MHz)

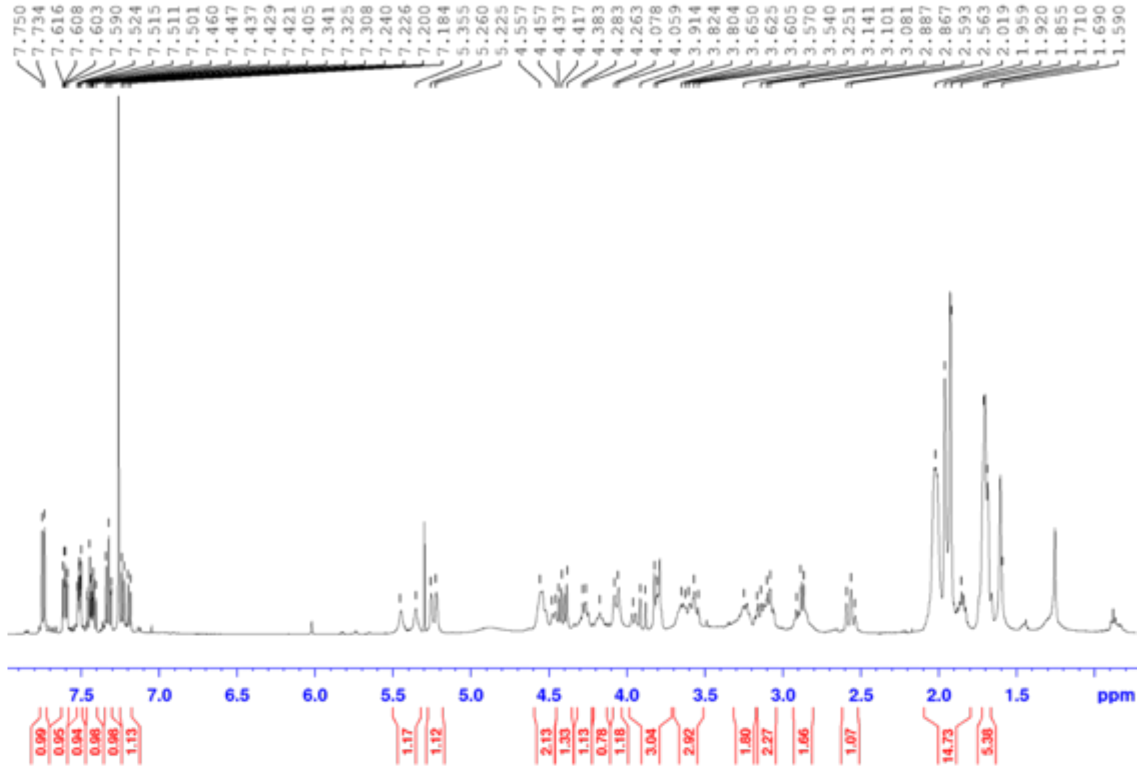

^{13}C NMR ($CDCl_3$, 125 MHz)

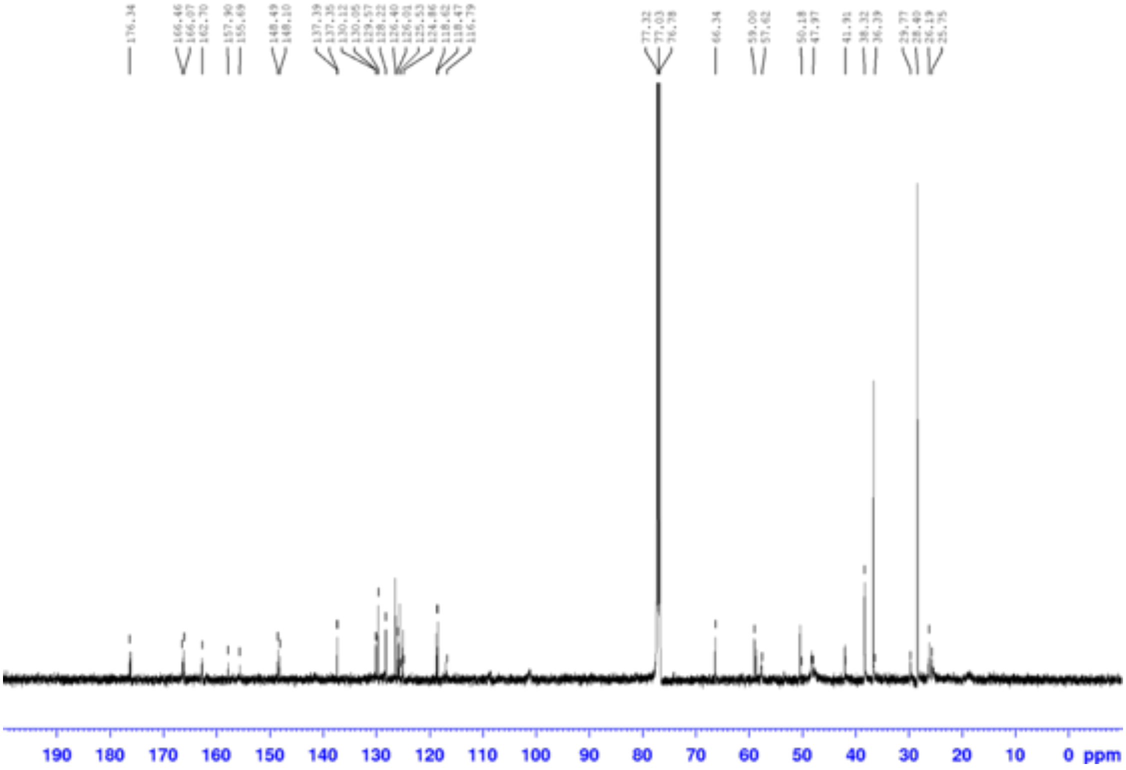

Compound HY3

^{1}H NMR ($CDCl_3$, 500 MHz)

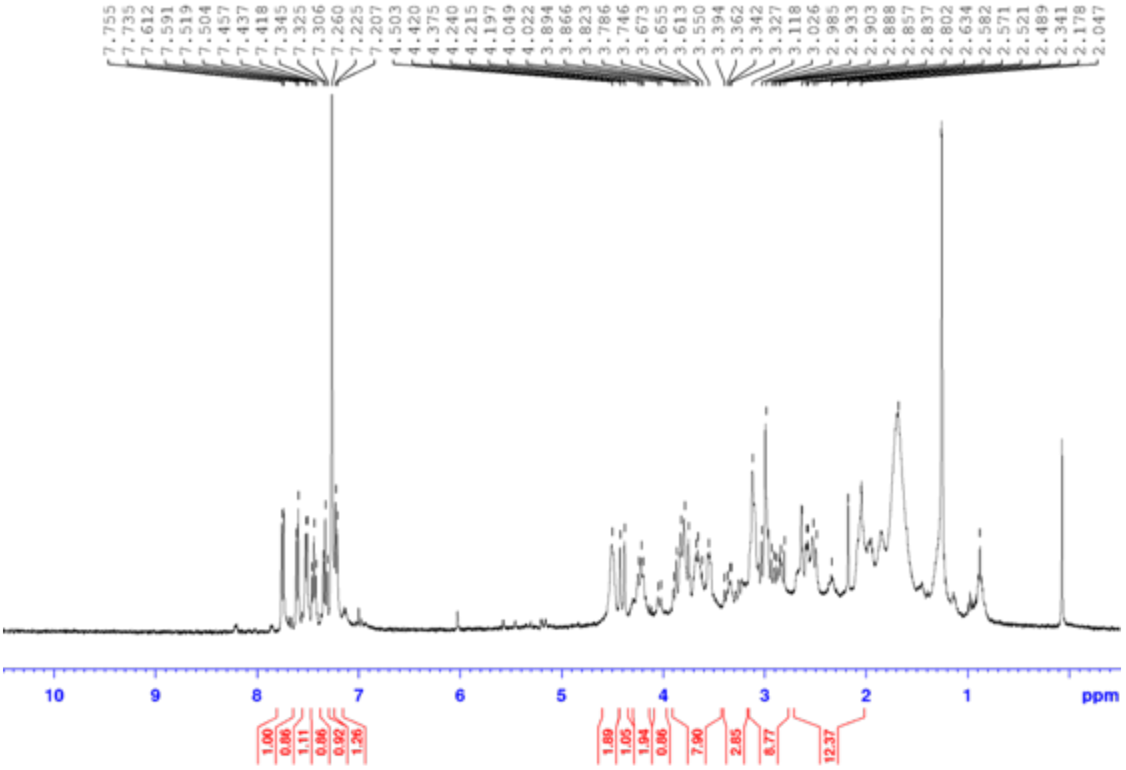

^{13}C NMR ($CDCl_3$, 125 MHz)

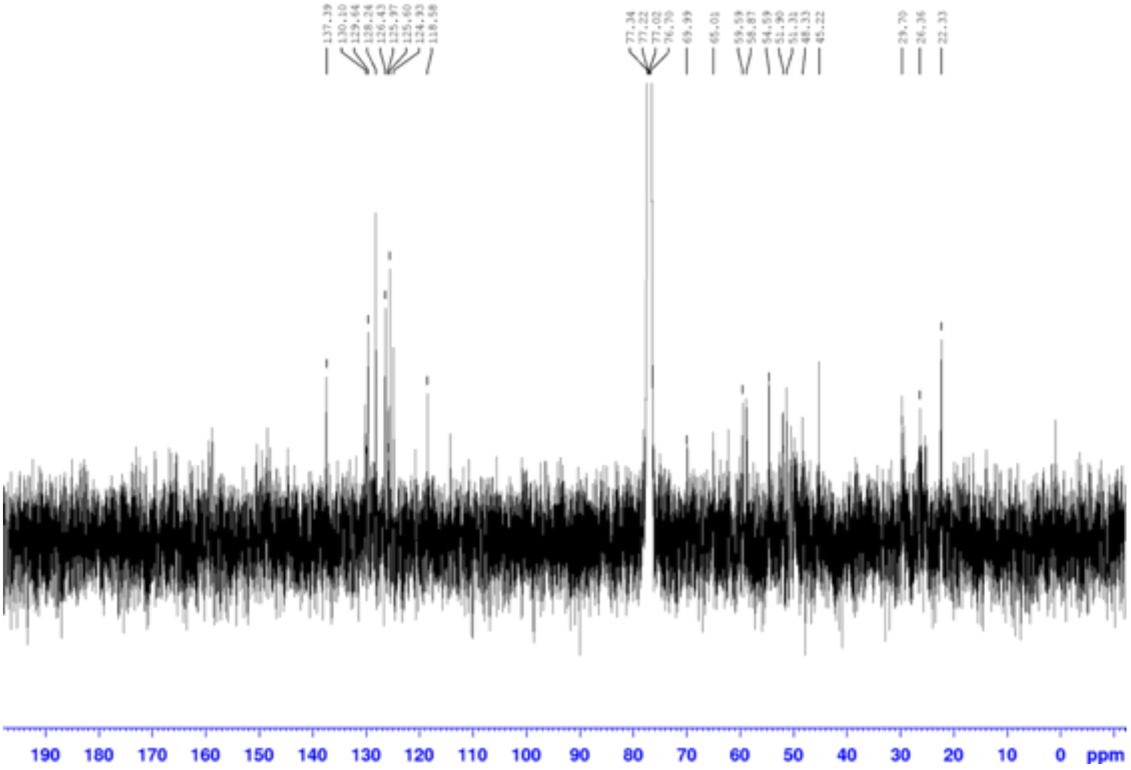

Compound HY4

^{1}H NMR ($CDCl_3$, 500 MHz)

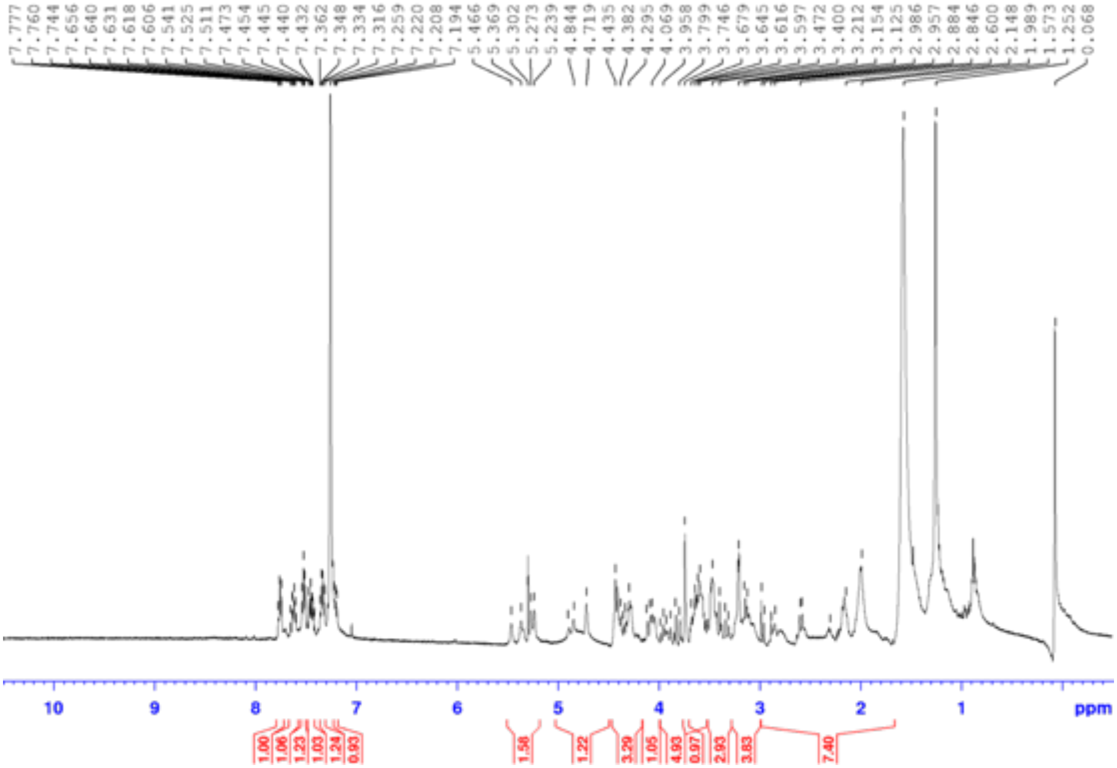

^{13}C NMR ($CDCl_3$, 125 MHz)

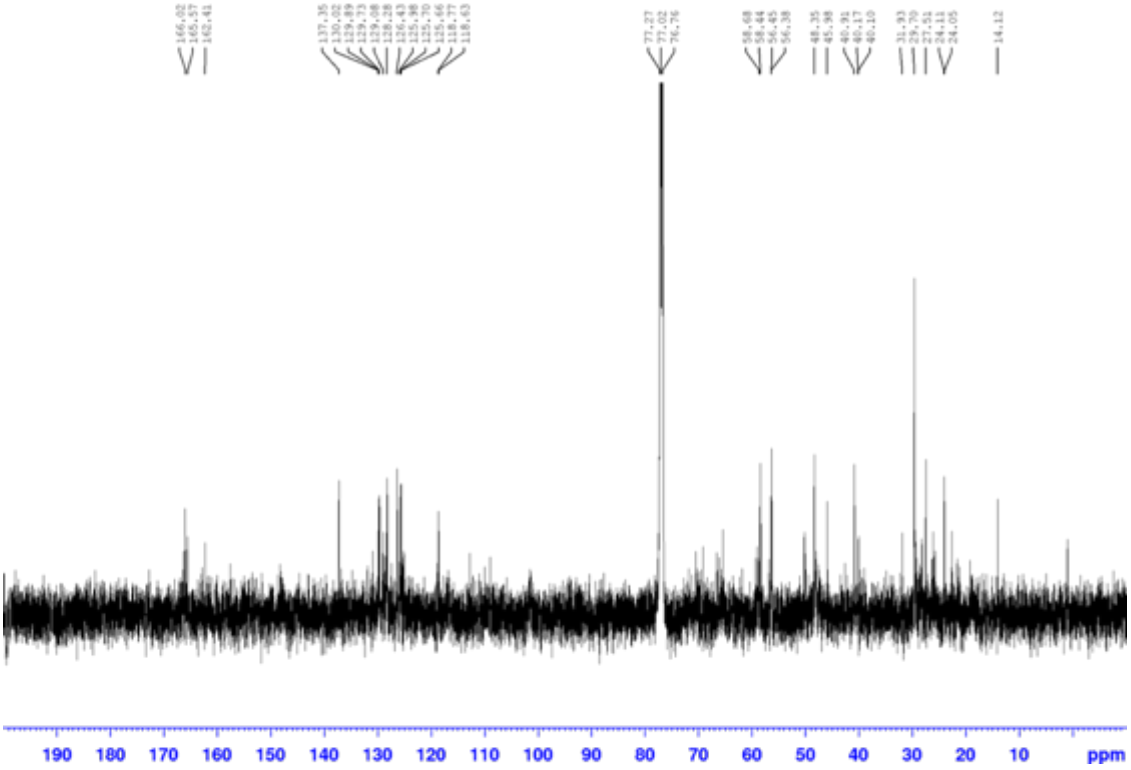

Compound HY5

^{1}H NMR ($CDCl_3$, 500 MHz)

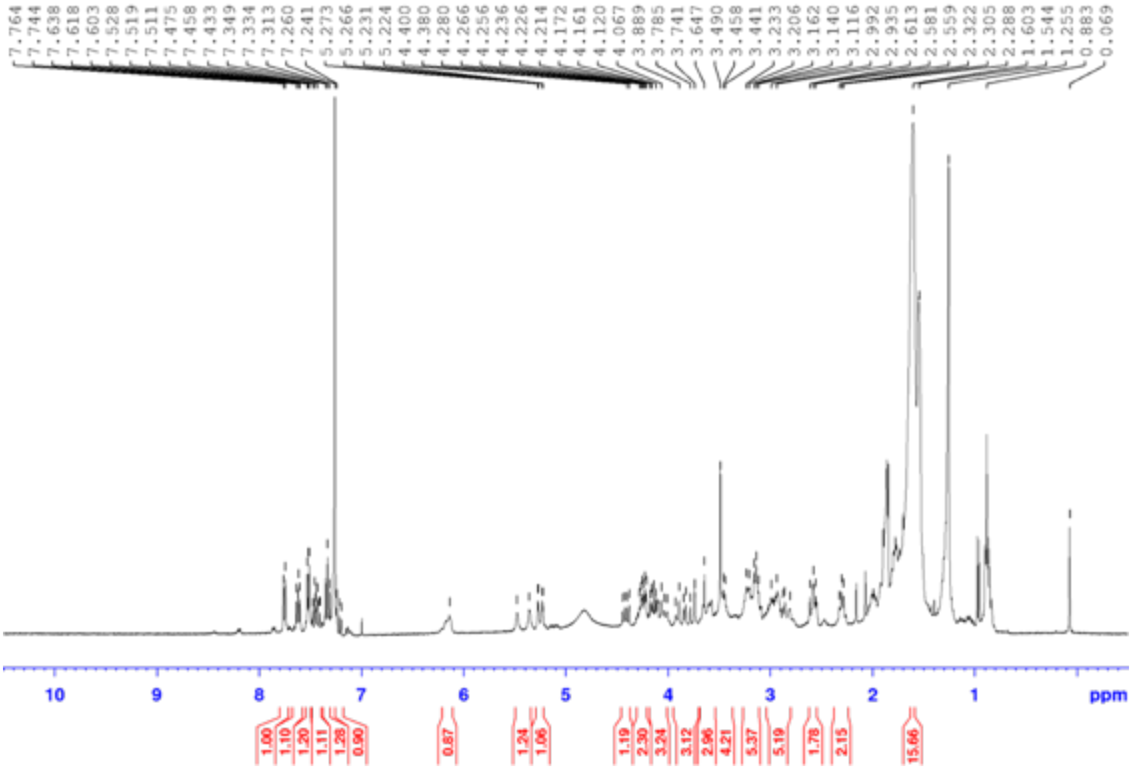

^{13}C NMR ($CDCl_3$, 125 MHz)

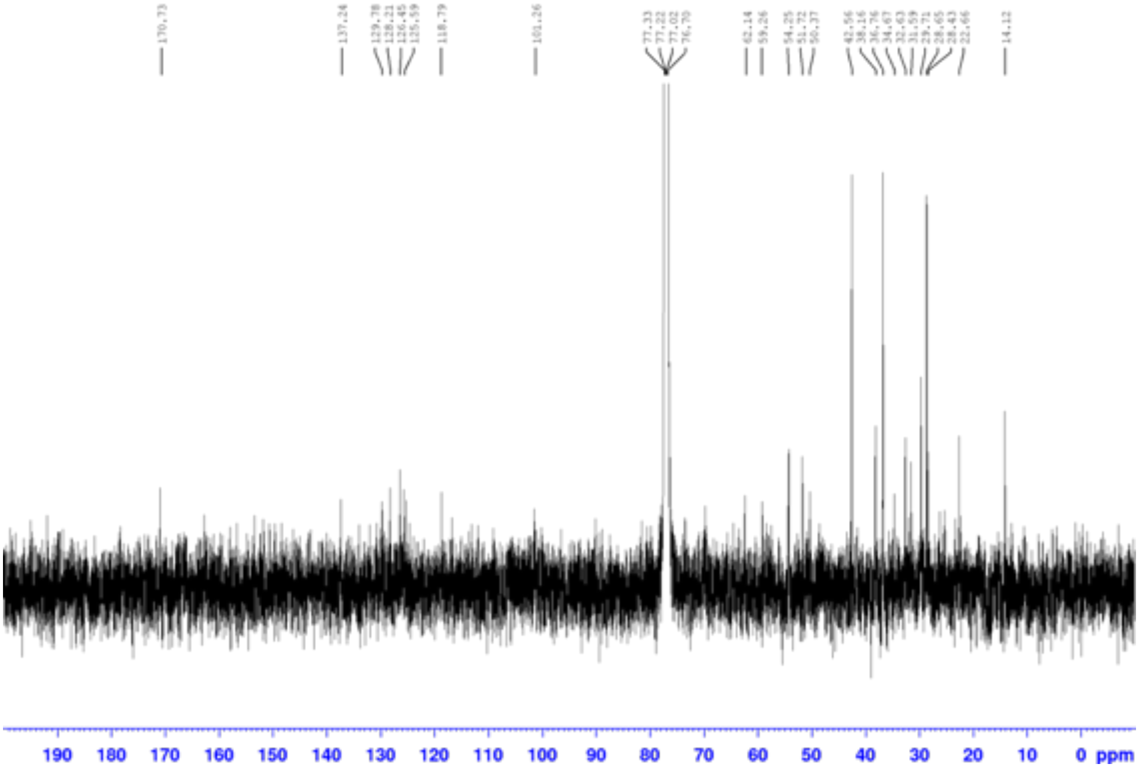

Compound HY6

^1H NMR ($CDCl_3$, 500 MHz)

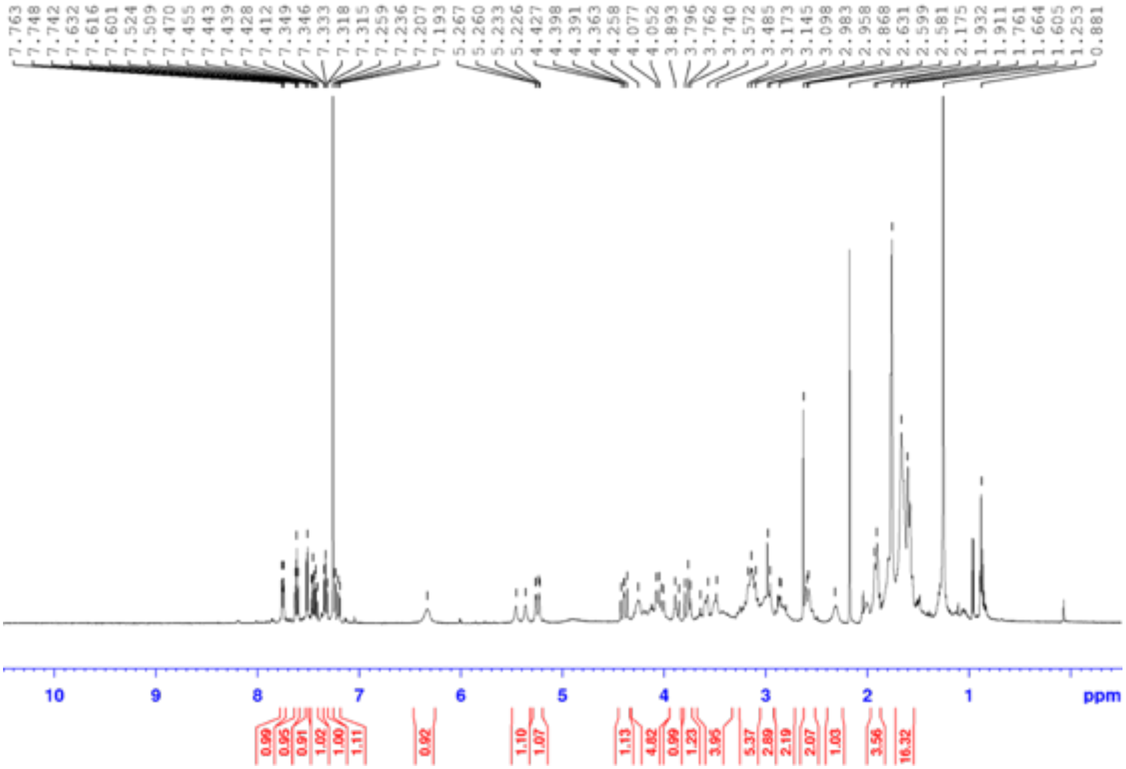

^{13}C NMR ($CDCl_3$, 125 MHz)

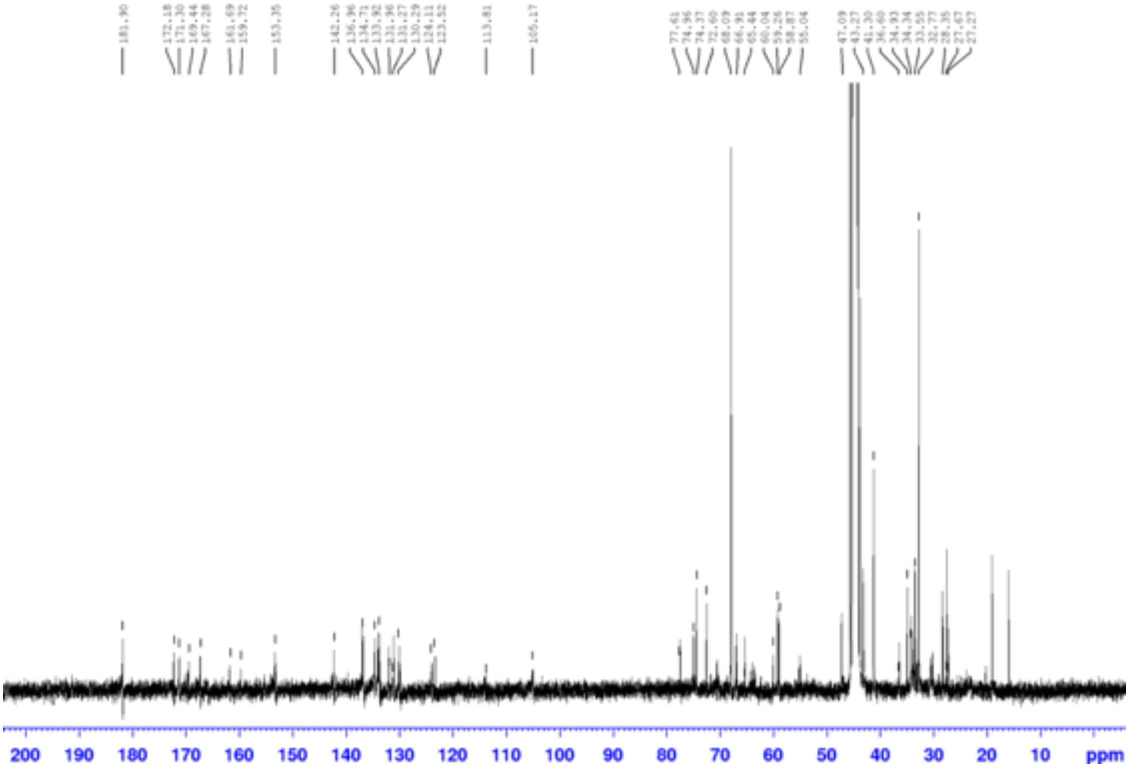

Compound HY7

^{1}H NMR ($CDCl_3$, 400 MHz)

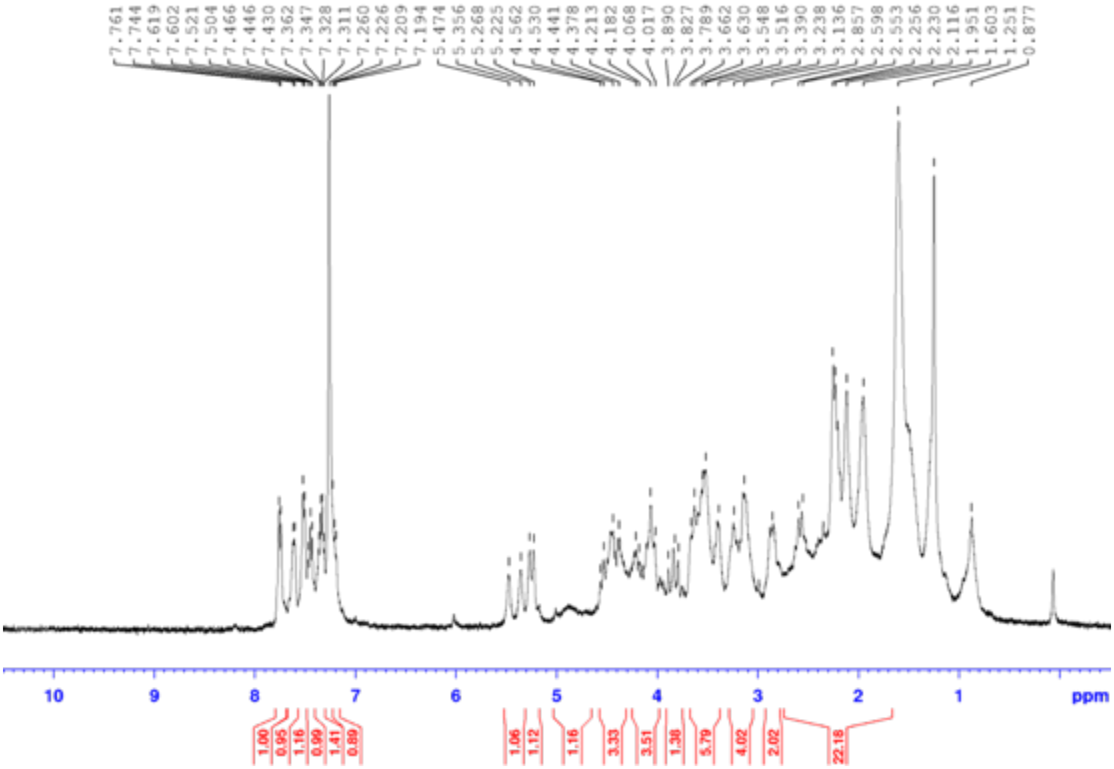

^{13}C NMR ($CDCl_3$, 100 MHz)

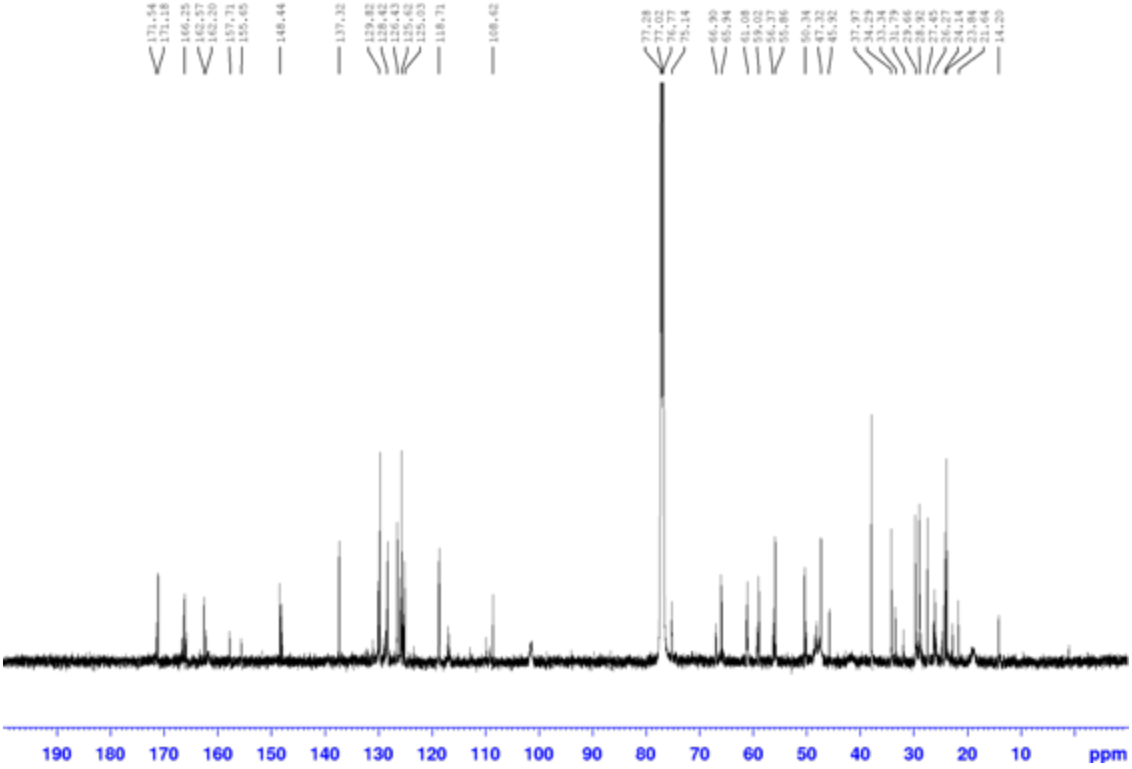

Compound HY8

^{1}H NMR ($CDCl_3$, 500 MHz)

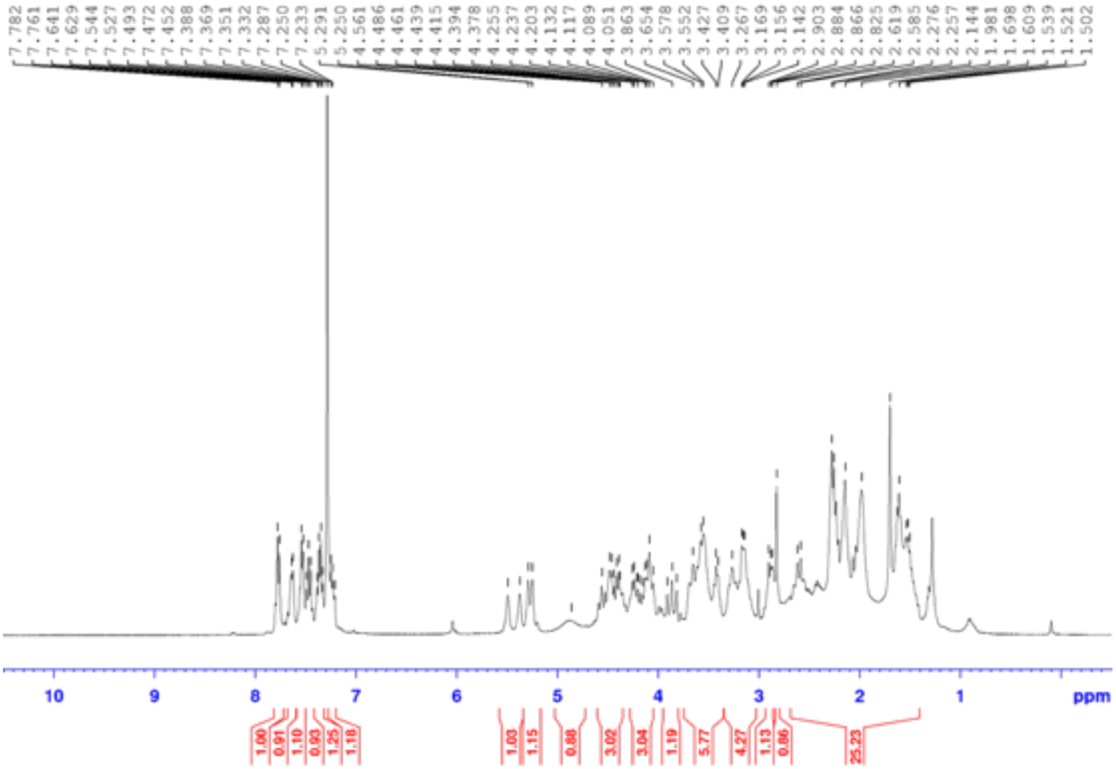

^{13}C NMR ($CDCl_3$, 125 MHz)

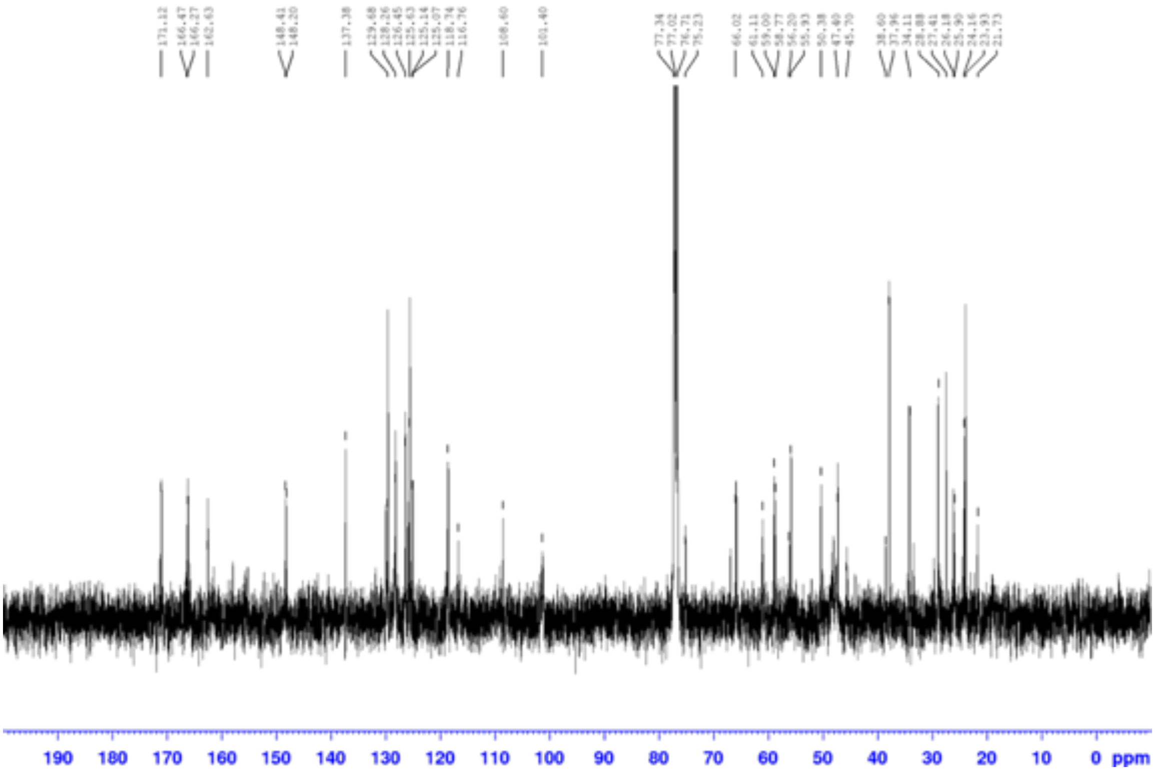

Compound 12

^{1}H NMR ($CDCl_3$, 400 MHz)

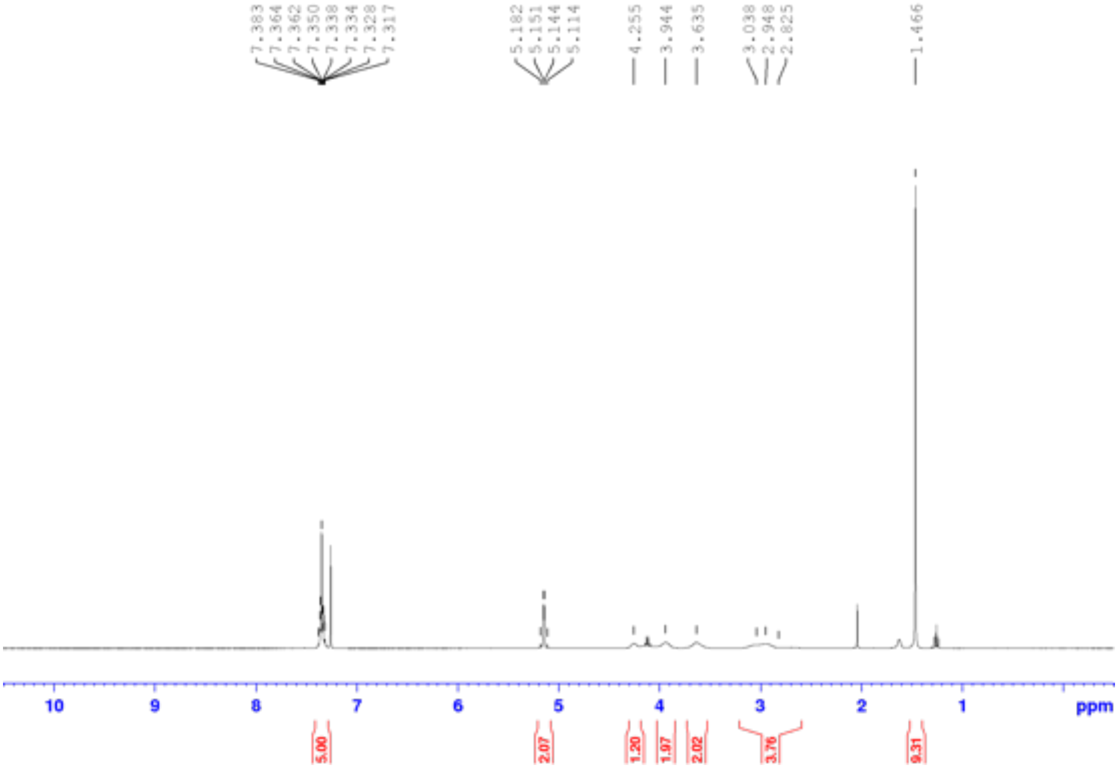

Compound 14

^{1}H NMR ($CDCl_3$, 400 MHz)

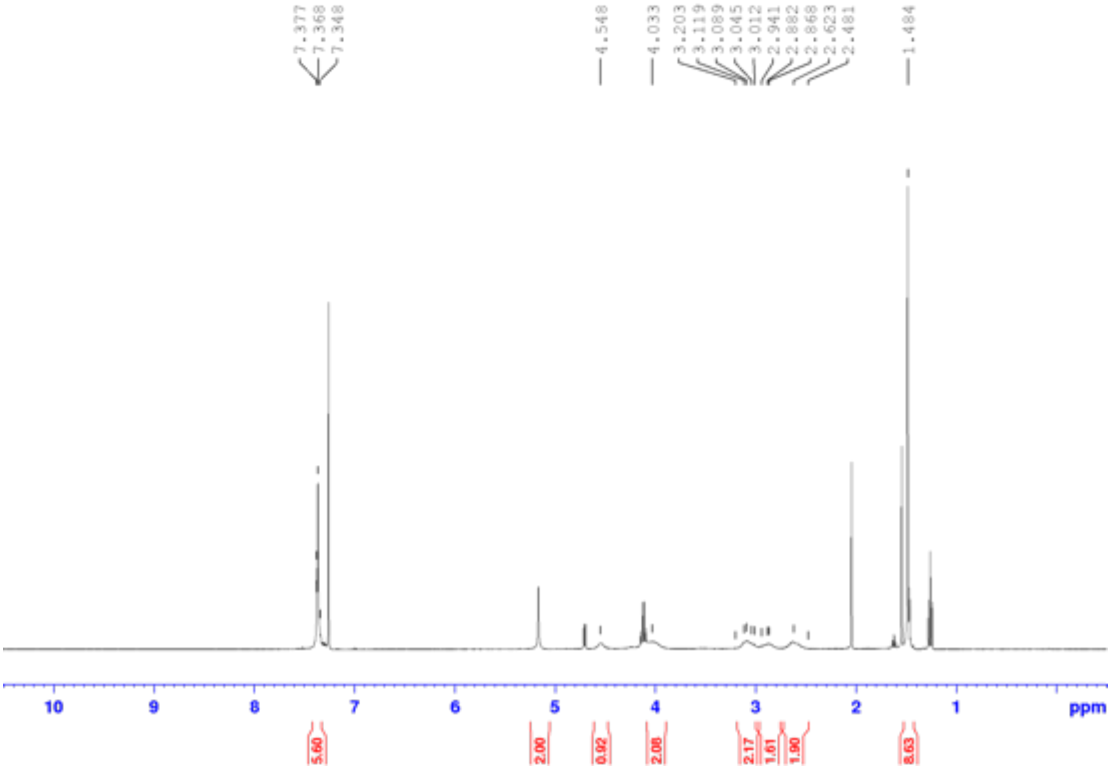

Compound 15

^{1}H NMR ($CDCl_3$, 400 MHz)

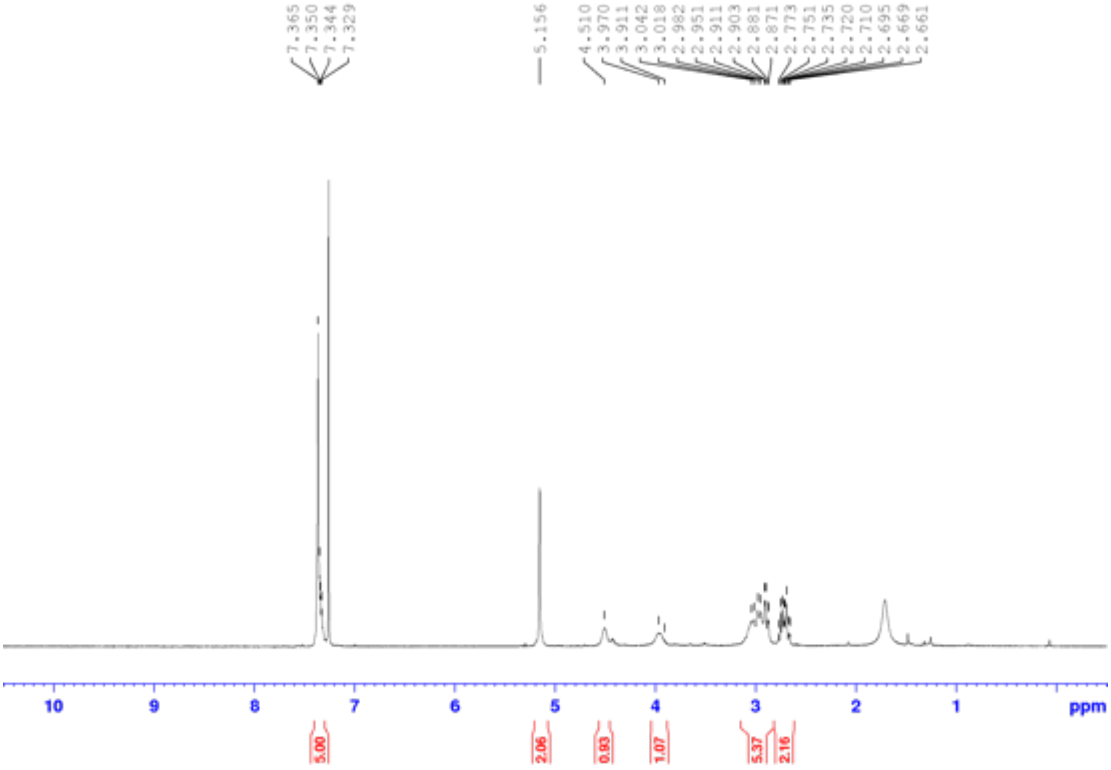

Compound 7-1

^{1}H NMR ($CDCl_3$, 400 MHz)

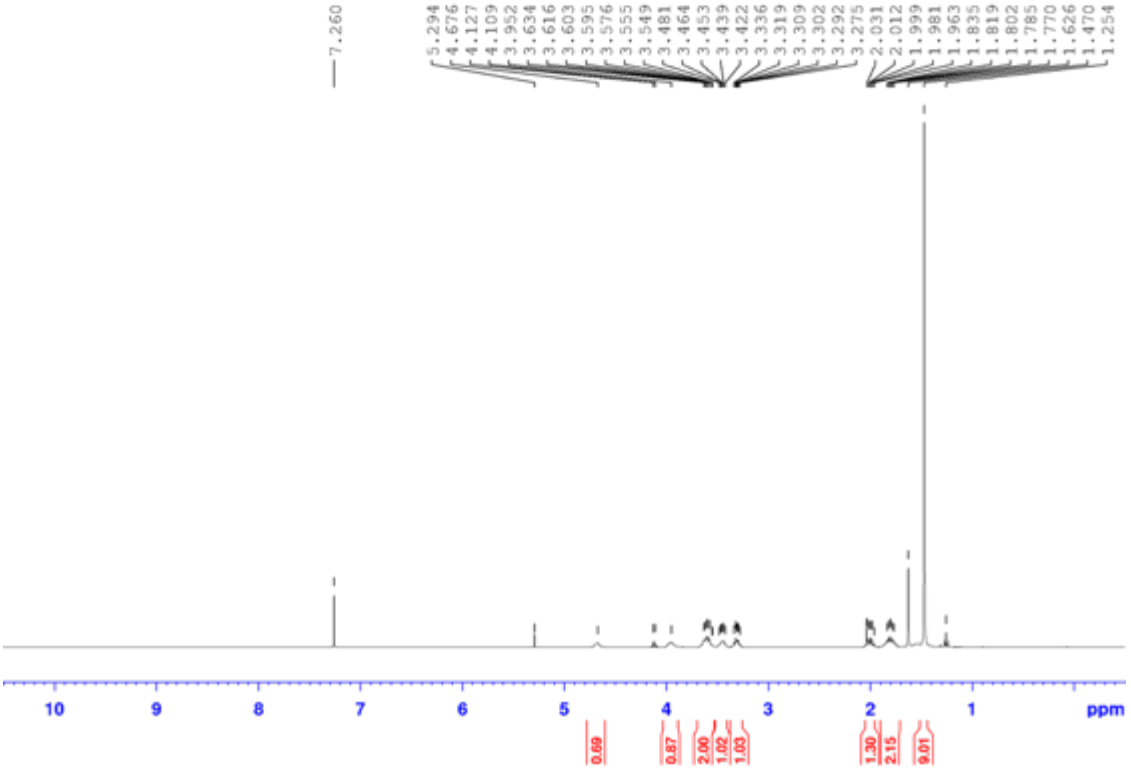

Compound 17

^{1}H NMR ($CDCl_3$, 400 MHz)

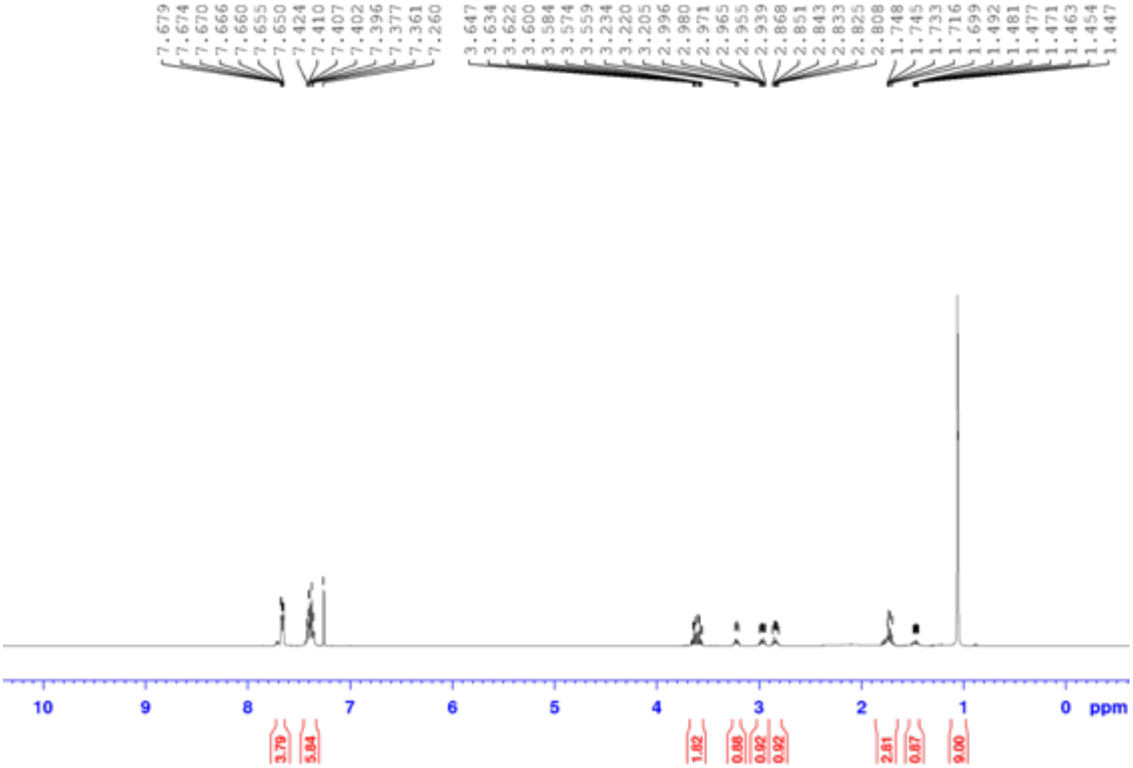

Compound 18

^1H NMR ($CDCl_3$, 400 MHz)

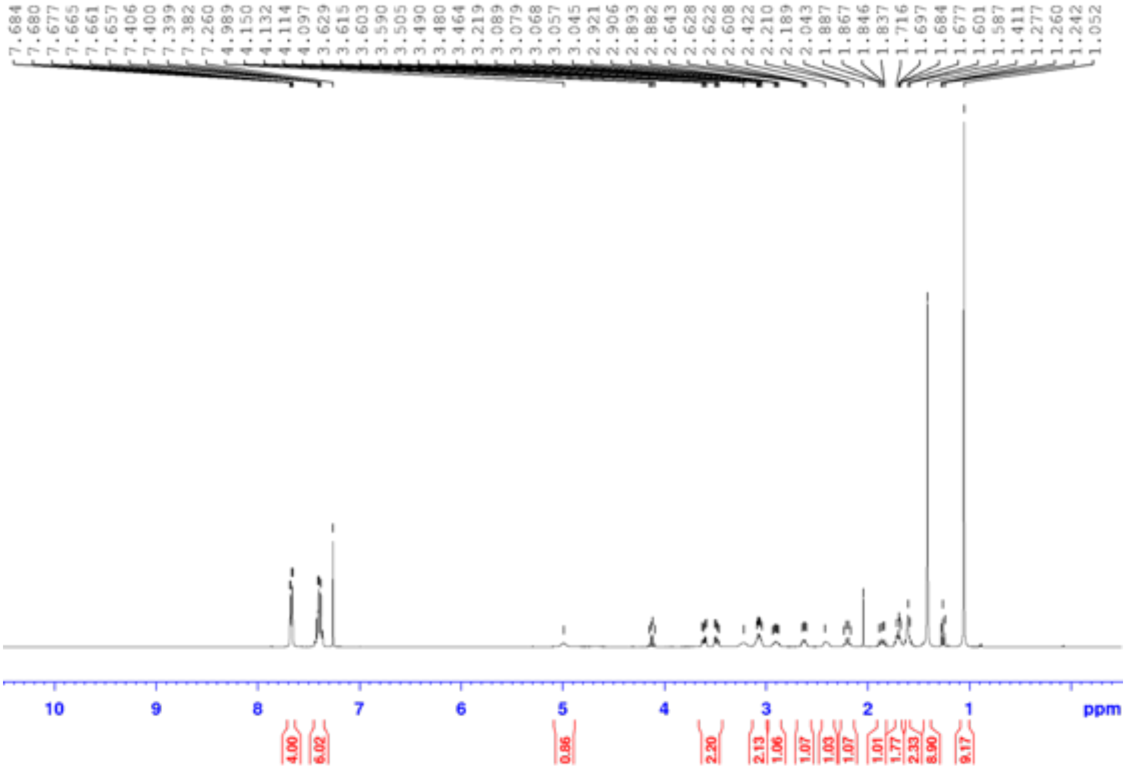

Compound 7-2

^1H NMR ($CDCl_3$, 400 MHz)

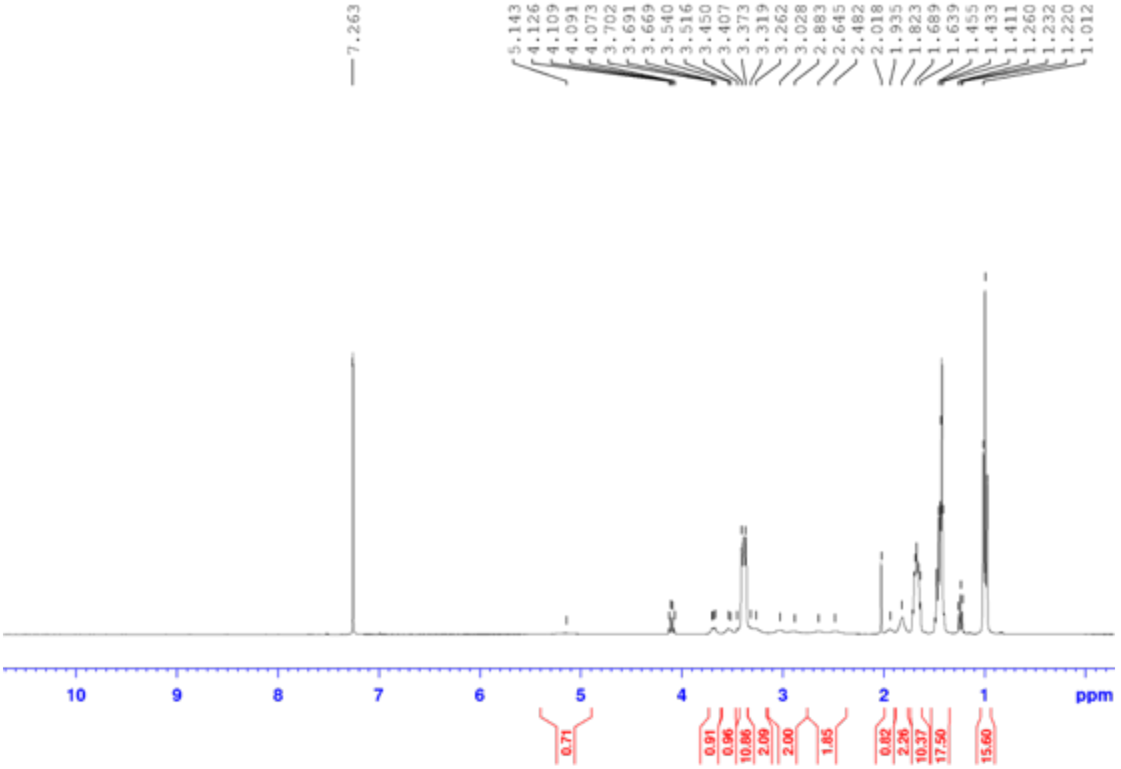

Compound 20

^{1}H NMR ($CDCl_3$, 400 MHz)

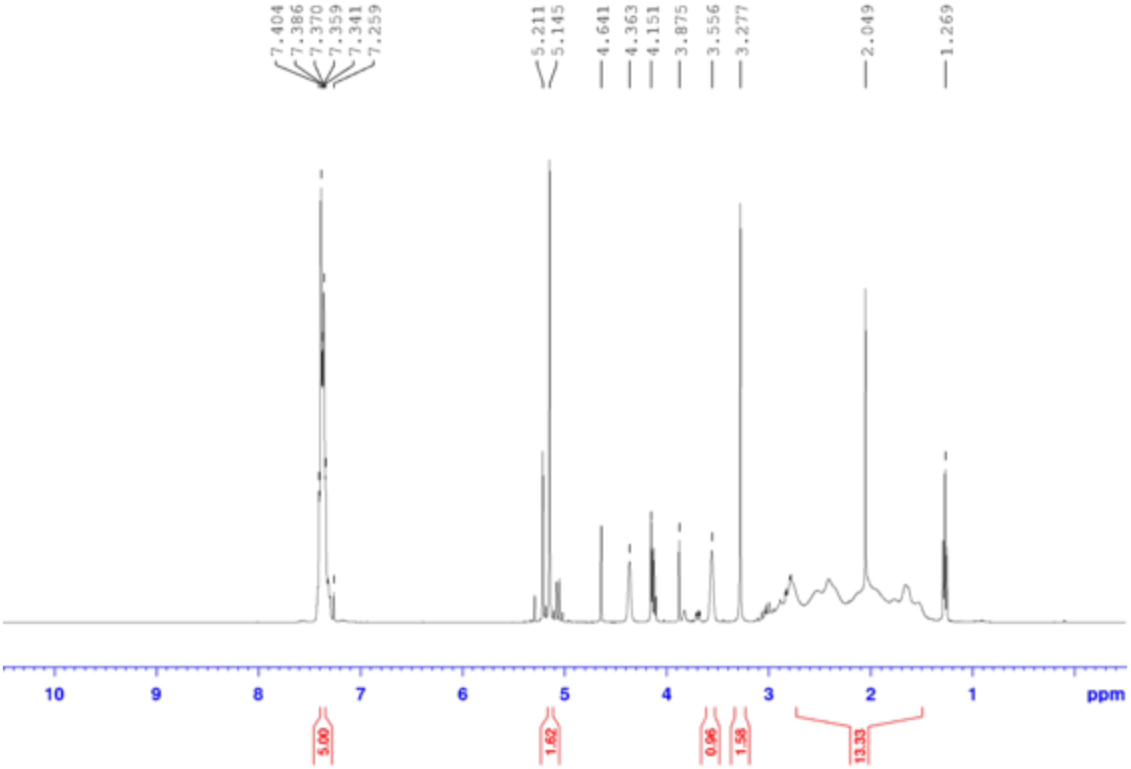

Compound 22

^{1}H NMR ($CDCl_3$, 400 MHz)

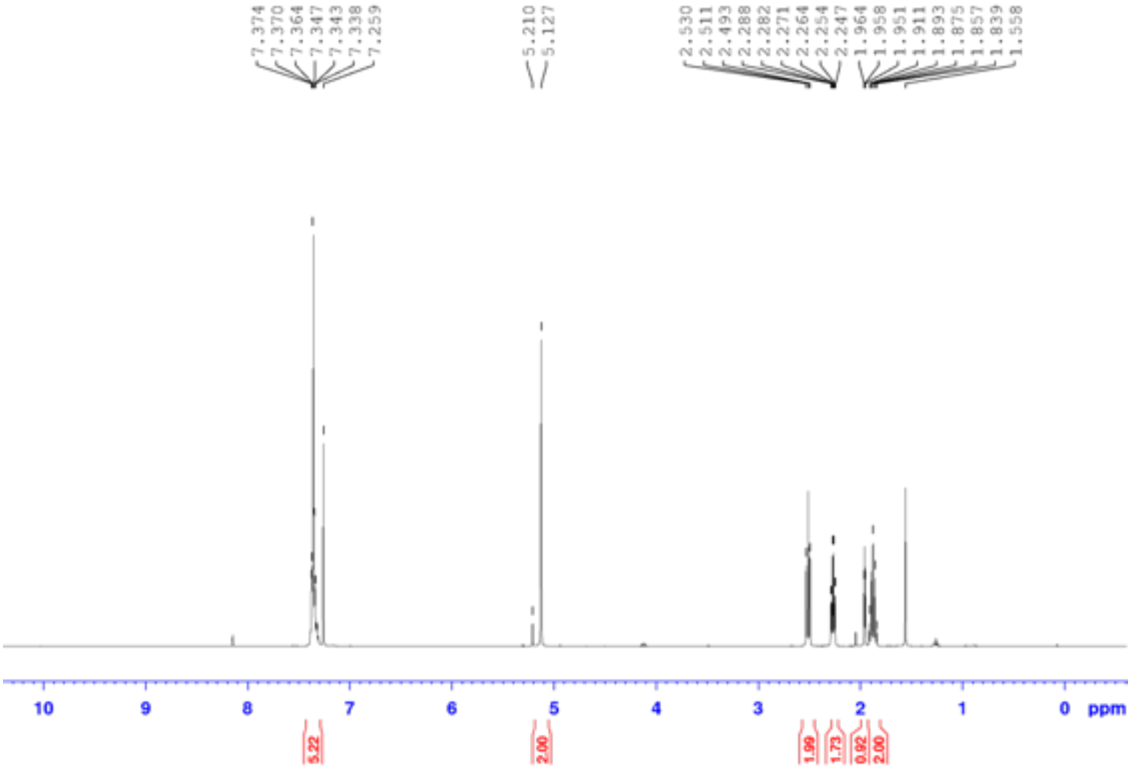

Compound 23

^{1}H NMR ($CDCl_3$, 400 MHz)

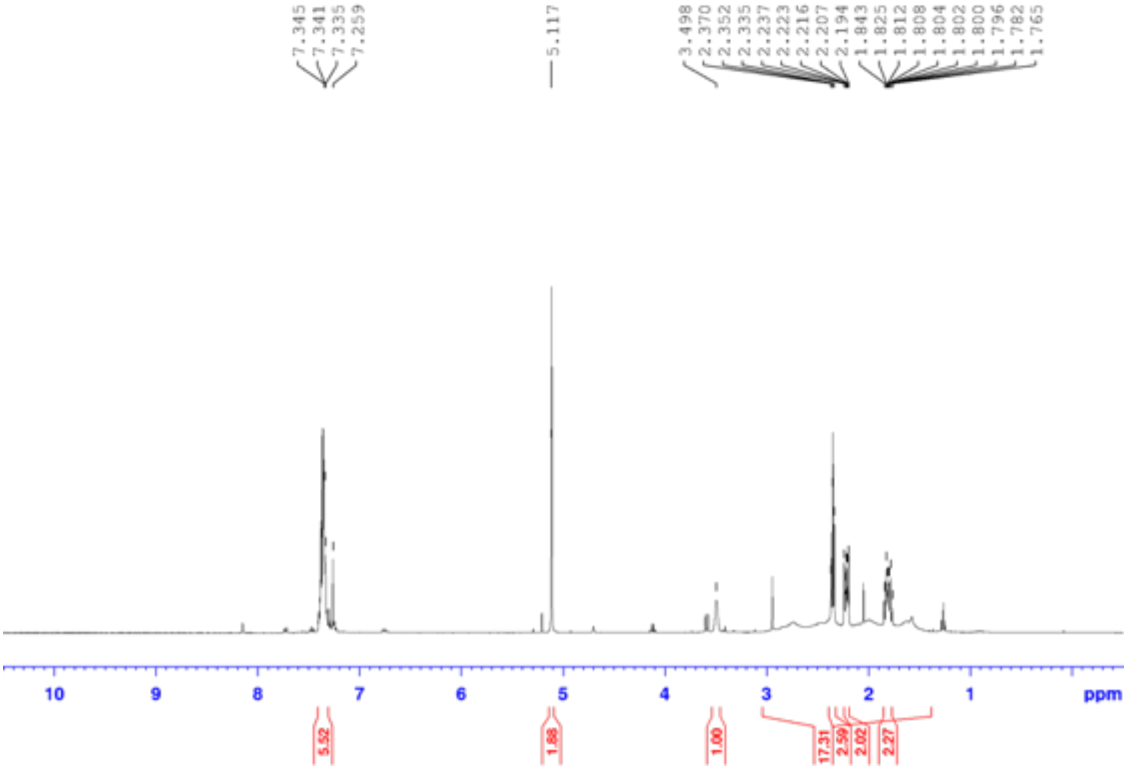

Compound 24

^{1}H NMR (DMSO-d_6, 400 MHz)

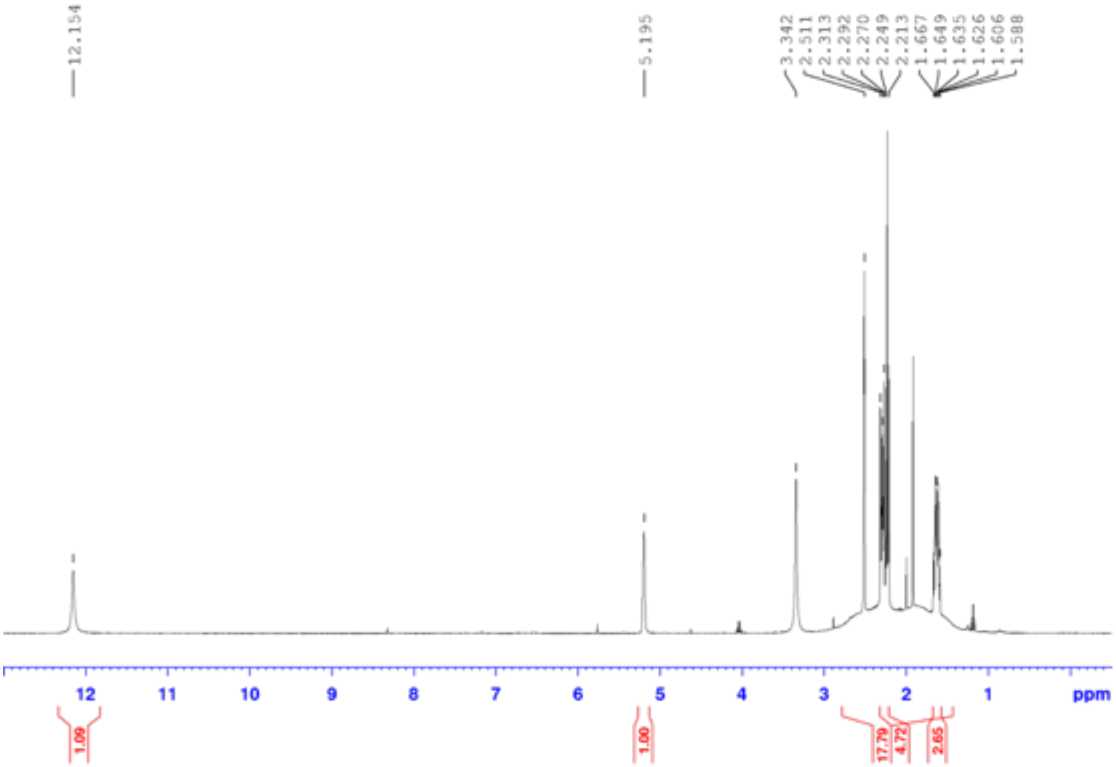

Compound 26

^1H NMR (DMSO-d_6, 400 MHz)

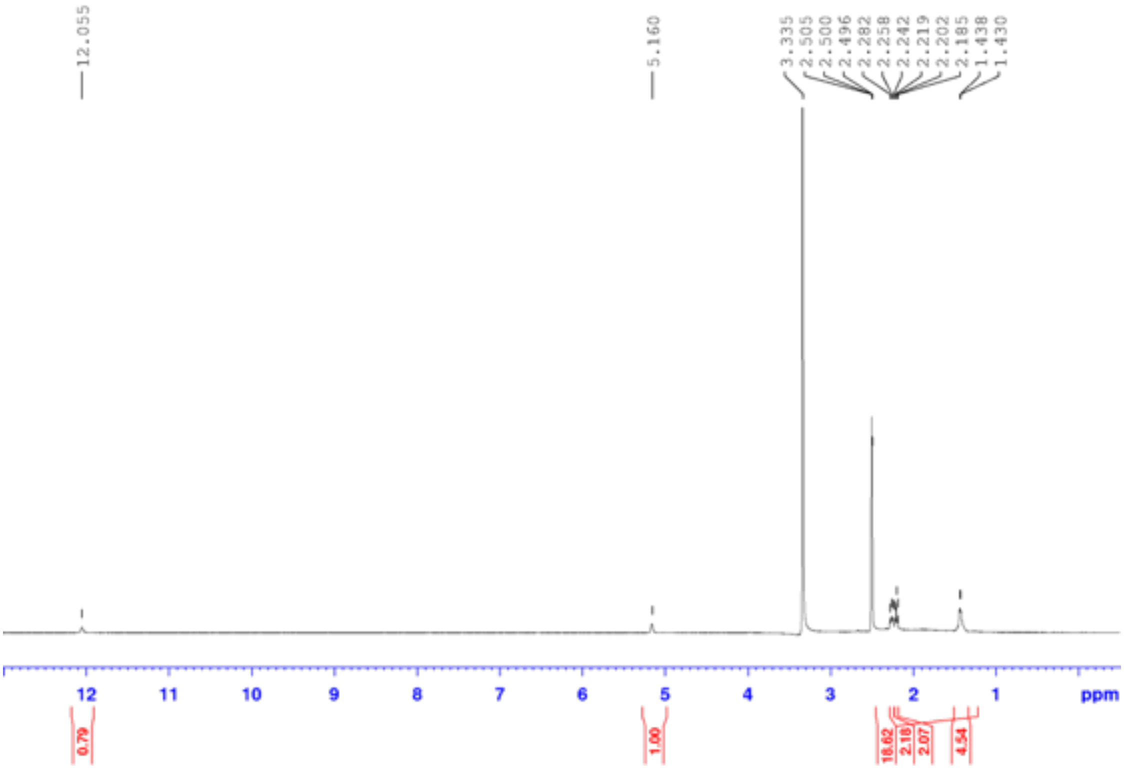

HPLC analysis of tested compound

Compound HY1

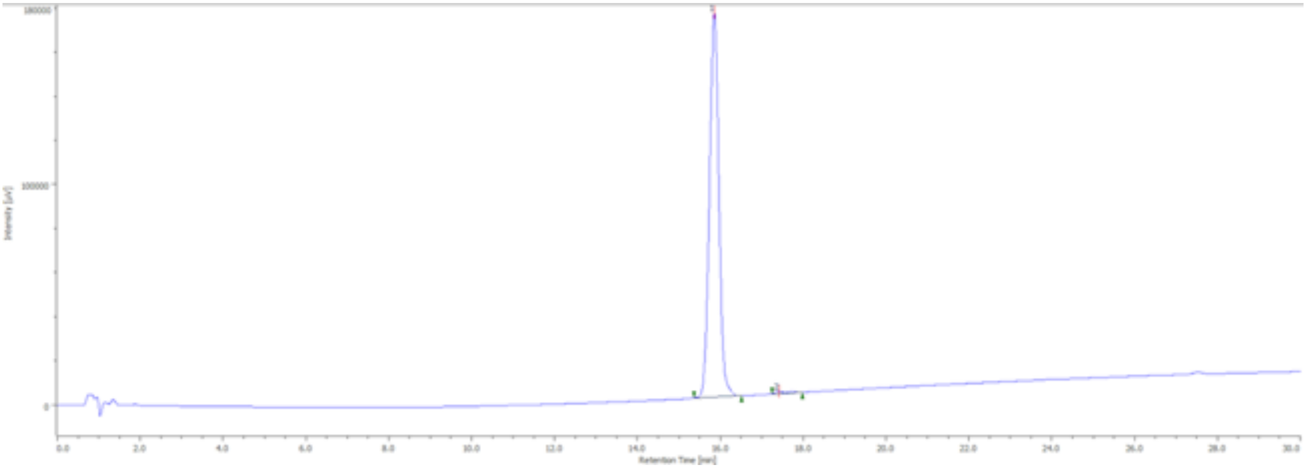

Peak	Area (%)	Retention time (min)
1 (**HY1**)	98.320	15.85
2 (unknown)	1.680	17.41

Compound HY2

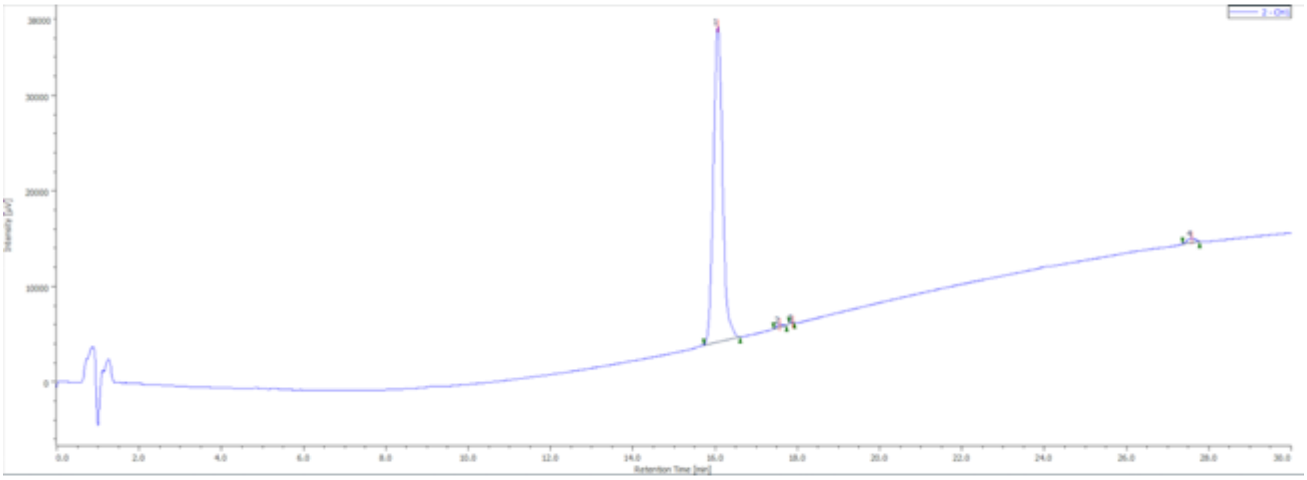

Peak	Area (%)	Retention time (min)
1 (**HY2**)	97.647	16.058
2 (unknown)	0.809	17.567
3 (unknown)	0.196	17.875
4 (unknown)	1.348	27.567

Compound HY3

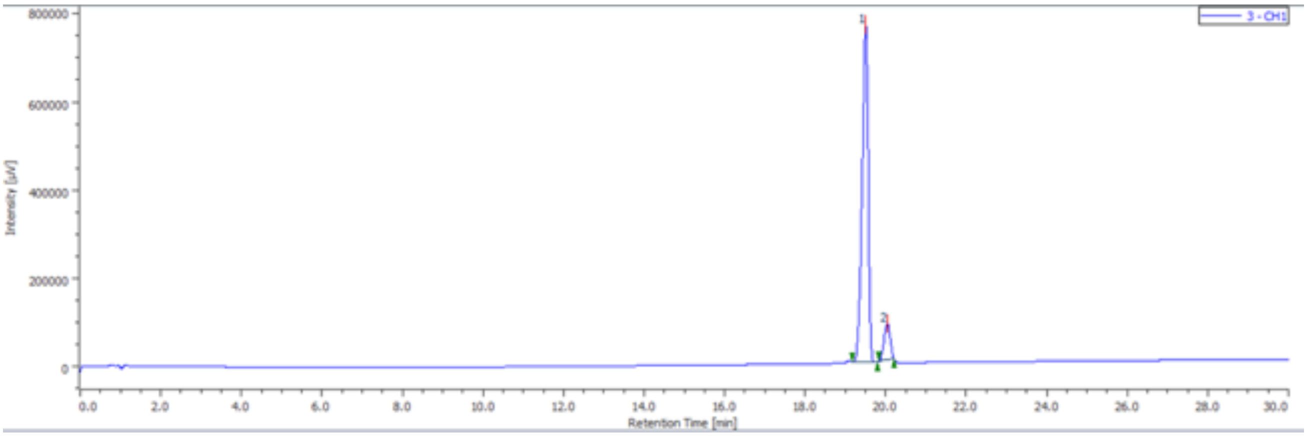

Peak	Area (%)	Retention time (min)
1 (**HY3**)	95.225	19.492
2 (unknown)	4.775	20.025

Compound HY4

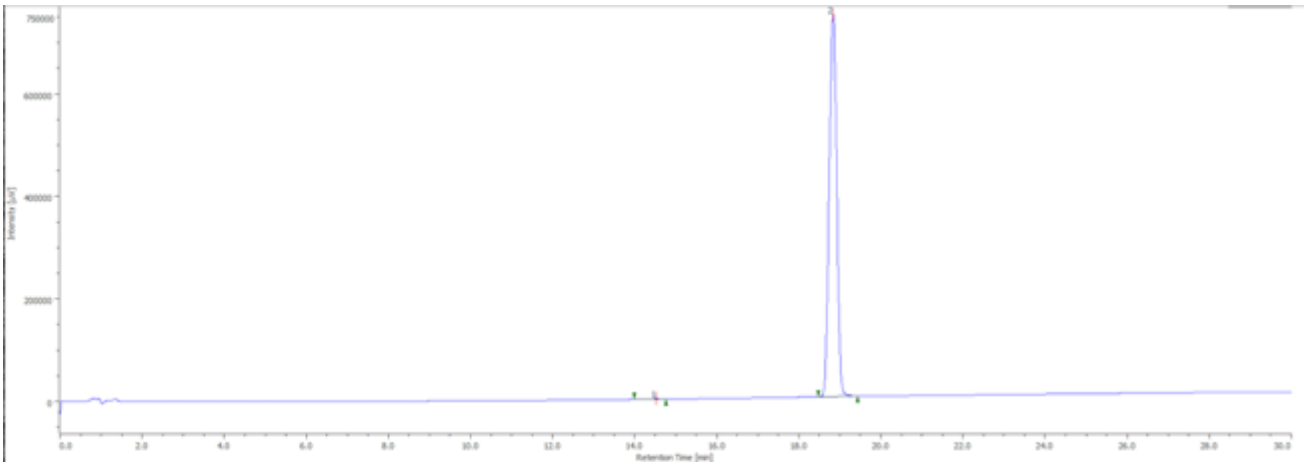

Peak	Area (%)	Retention time (min)
1 (unknown)	0.106	14.517
2 (**HY4**)	99.894	18.825

Compound HY5

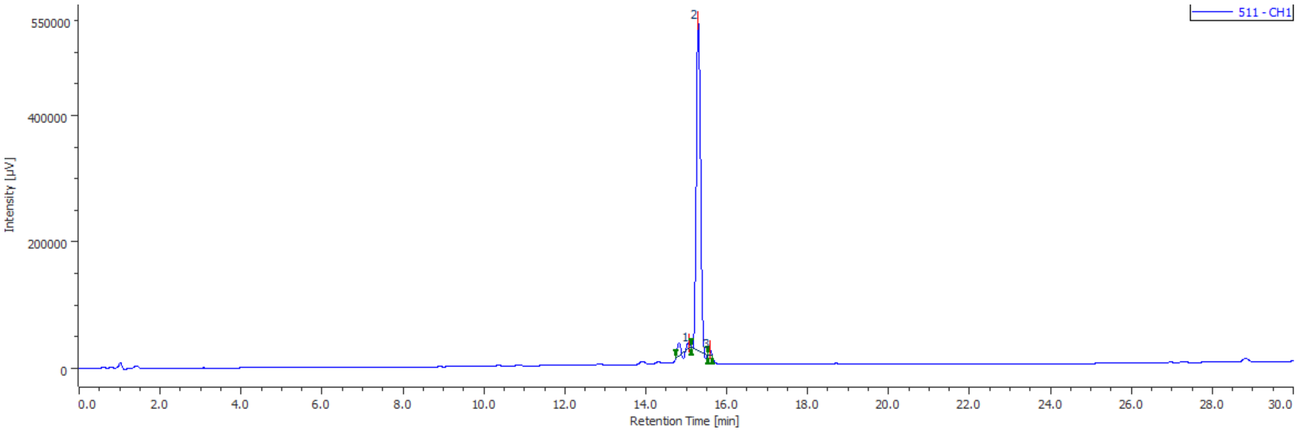

Peak	Area (%)	Retention time (min)
1 (unknown)	3.520	15.042
2 (**HY5**)	96.925	15.282
3 (unknown)	0.947	15.567

Compound HY6

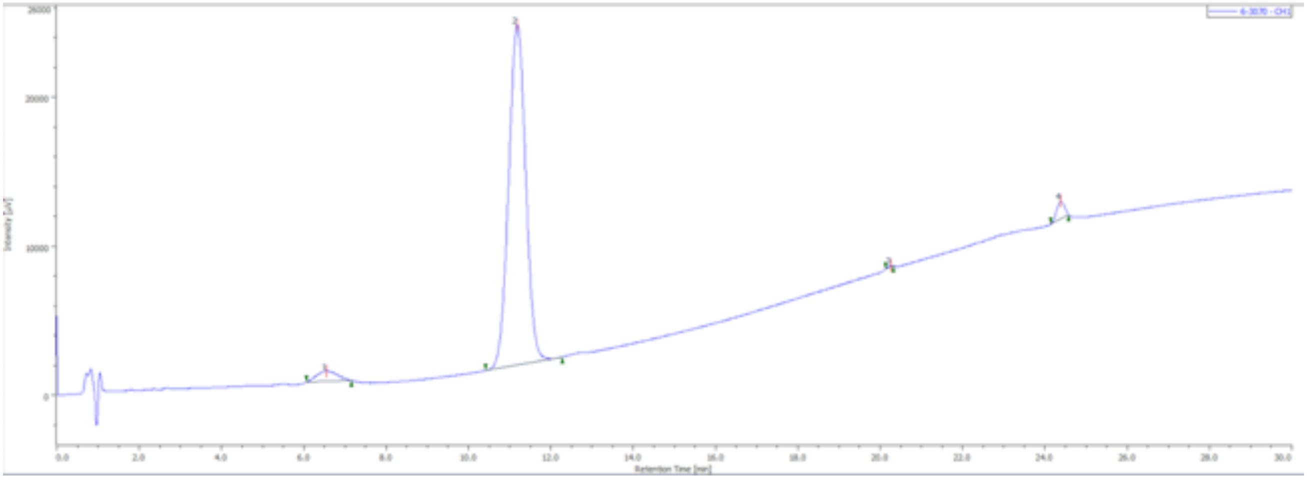

Peak	Area (%)	Retention time (min)
1 (unknown)	2.494	6.550
2 (**HY6**)	95.188	11.175
3 (unknown)	0.110	20.250
4 (unknown)	2.207	24.375

Compound HY7

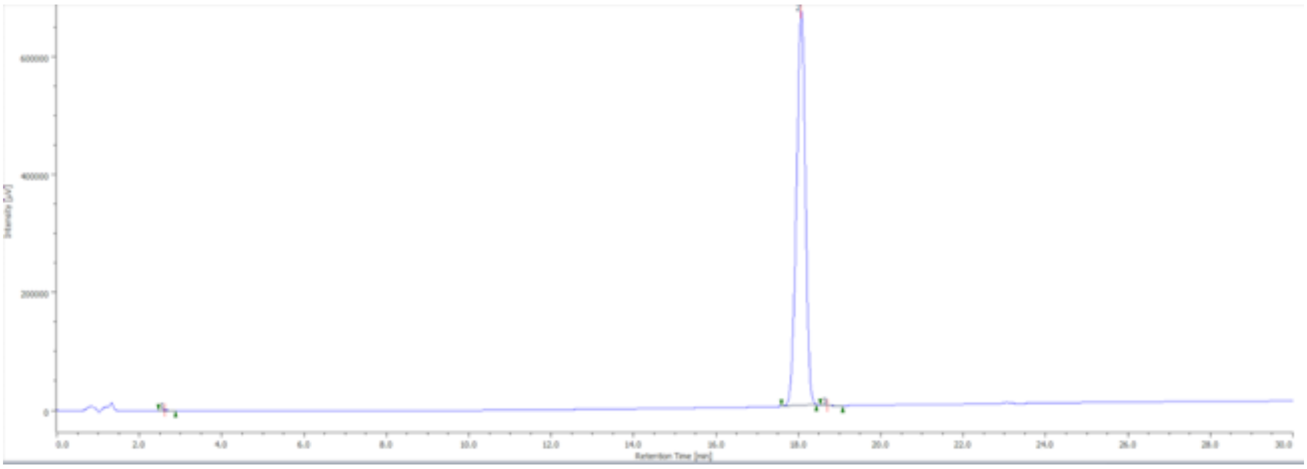

Peak	Area (%)	Retention time (min)
1 (unknown)	0.198	2.625
2 (**HY7**)	97.782	18.058
3 (unknown)	1.119	18.692

Compound HY8

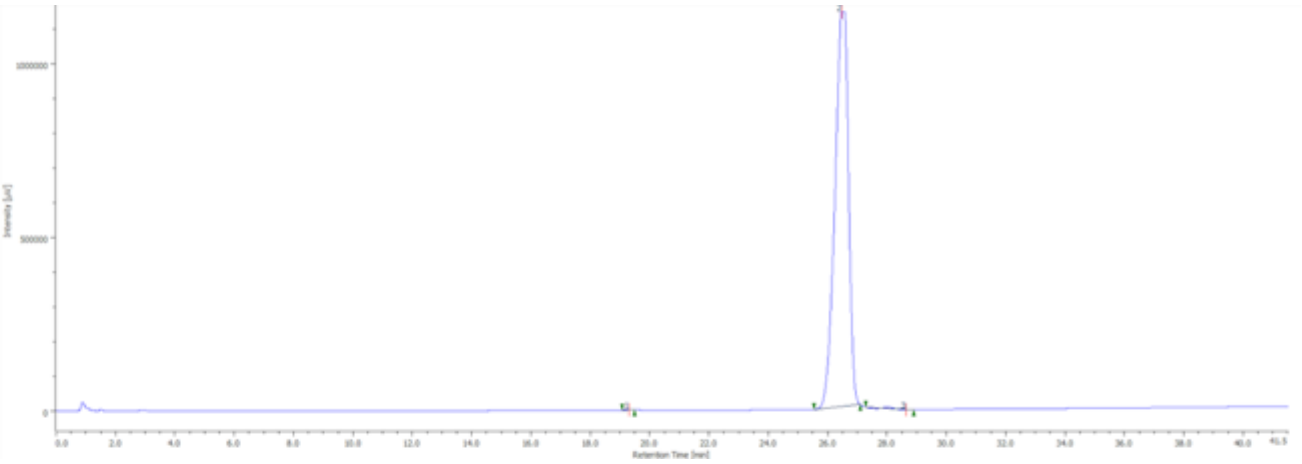

Peak	Area (%)	Retention time (min)
1 (unknown)	1.316	19.300
2 (**HY8**)	98.026	26.467
3 (unknown)	0.658	28.617

4. References

(1) Bond, M. J.; Chu, L.; Nalawansha, D. A.; Li, K.; Crews, C. M. Targeted Degradation of Oncogenic $KRAS^{G12C}$ by VHL-Recruiting PROTACs. *ACS Cent. Sci.* **2020**, *6* (8), 1367–1375. https://doi.org/10.1021/acscentsci.0c00411.

(2) Fell, J. B.; Fischer, J. P.; Baer, B. R.; Blake, J. F.; Bouhana, K.; Briere, D. M.; Brown, K. D.; Burgess, L. E.; Burns, A. C.; Burkard, M. R.; Chiang, H.; Chicarelli, M. J.; Cook, A. W.; Gaudino, J. J.; Hallin, J.; Hanson, L.; Hartley, D. P.; Hicken, E. J.; Hingorani, G. P.; Hinklin, R. J.; Mejia, M. J.; Olson, P.; Otten, J. N.; Rhodes, S. P.; Rodriguez, M. E.; Savechenkov, P.; Smith, D. J.; Sudhakar, N.; Sullivan, F. X.; Tang, T. P.; Vigers, G. P.; Wollenberg, L.; Christensen, J. G.; Marx, M. A. Identification of the Clinical Development Candidate **MRTX849** , a Covalent $KRAS^{G12C}$ Inhibitor for the Treatment of Cancer. *J. Med. Chem.* **2020**, *63* (13), 6679–6693. https://doi.org/10.1021/acs.jmedchem.9b02052.

(3) Yang, R.; Qi, L.; Liu, Y.; Ding, Y.; Kwek, M. S. Y.; Liu, C.-F. Chemical Synthesis of N-Peptidyl 2-Pyrrolidinemethanethiol for Peptide Ligation. *Tetrahedron Lett.* **2013**, *54* (29), 3777–3780. https://doi.org/10.1016/j.tetlet.2013.05.013.

(4) Asawa, Y.; Nishida, K.; Kawai, K.; Domae, K.; Ban, H. S.; Kitazaki, A.; Asami, H.; Kohno, J.-Y.; Okada, S.; Tokuma, H.; Sakano, D.; Kume, S.; Tanaka, M.; Nakamura, H. Carborane as an Alternative Efficient Hydrophobic Tag for Protein Degradation. *Bioconjug. Chem.* **2021**, *32* (11), 2377–2385. https://doi.org/10.1021/acs.bioconjchem.1c00431.

(5) Kimura, S.; Masunaga, S.; Harada, T.; Kawamura, Y.; Ueda, S.; Okuda, K.; Nagasawa, H. Synthesis and Evaluation of Cyclic RGD-Boron Cluster Conjugates to Develop Tumor-Selective Boron Carriers for Boron Neutron Capture Therapy. *Bioorg. Med. Chem.* **2011**, *19* (5), 1721–1728. https://doi.org/10.1016/j.bmc.2011.01.020.

(6) Asawa, Y.; Hatsuzawa, S.; Yoshimori, A.; Yamada, K.; Katoh, A.; Kouji, H.; Nakamura, H. Comprehensive Exploration of Chemical Space Using Trisubstituted Carboranes. *Sci. Rep.* **2021**, *11*, 24101. https://doi.org/10.1038/s41598-021-24101-0.
